# Correction: Independent Transitions between Monsoonal and Arid Biomes Revealed by Systematic Revison of a Complex of Australian Geckos (*Diplodactylus*; Diplodactylidae)

**DOI:** 10.1371/journal.pone.0126682

**Published:** 2015-04-16

**Authors:** The PLOS ONE Staff

## Notice of Republication

This article was republished on April 8, 2015, to correct errors in the PDF layout that affected the scientific understanding of the article. The publisher apologizes for these errors that were introduced during the typesetting process. Please download this article again to view the correct version. The originally published, uncorrected article and the republished, corrected article are provided here for reference.

## Supporting Information

S1 File Originally published, uncorrected article. (PDF)

S2 File Republished corrected article. (PDF)
