# Supplementary figures and images for: Independent Transitions between Monsoonal and Arid Biomes Revealed by Systematic Revison of a Complex of Australian Geckos (Diplodactylus; Diplodactylidae)
Source: PLoS One. 2014 Dec 10;9(12):e111895. doi: 10.1371/journal.pone.0111895 (PMC4262211; doi:10.1371/journal.pone.0111895)

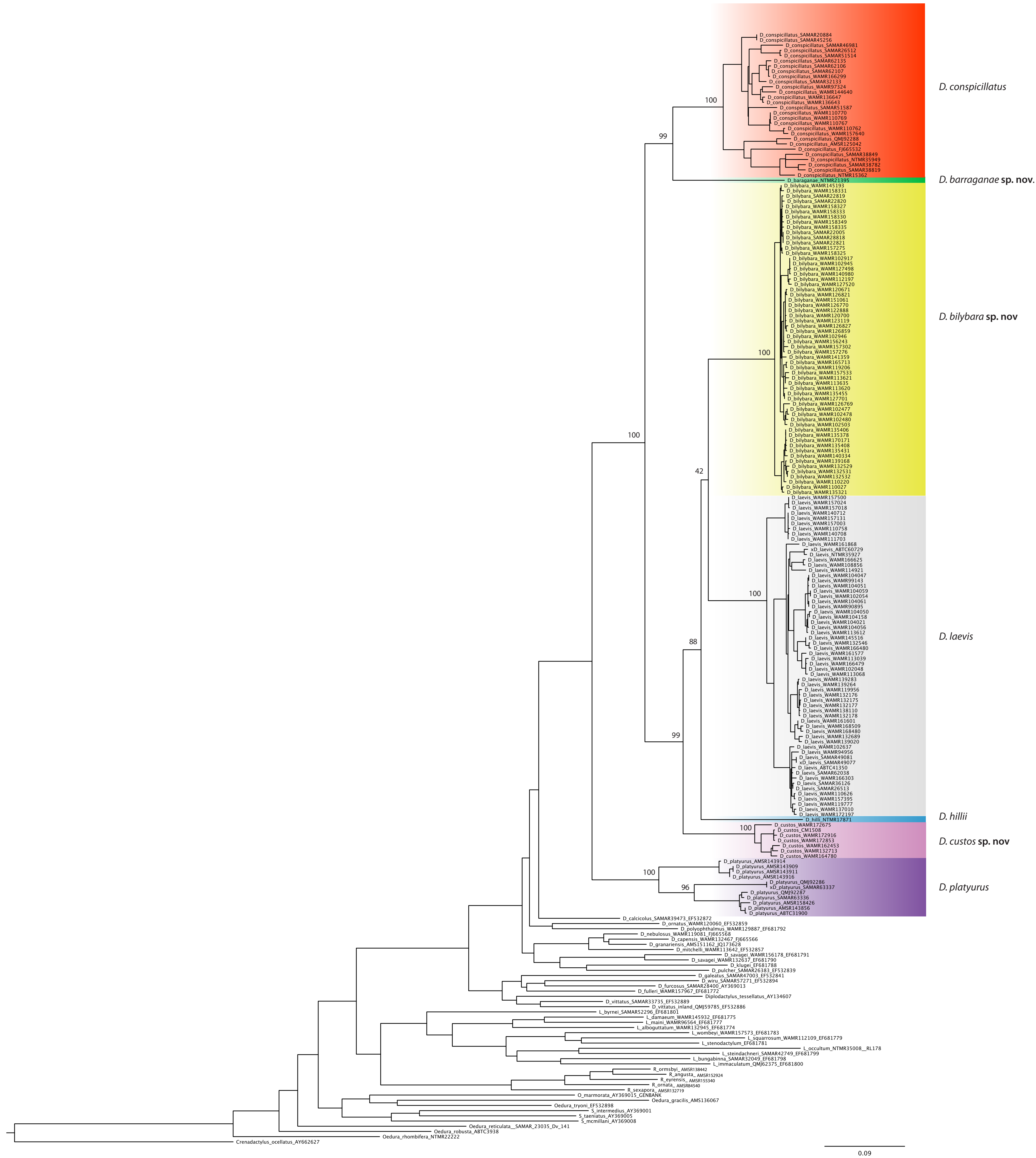

Supplement: S1 Fig — Estimated from mitochondrial ND2 data using RAxML with Maximum Likelihood support Boostrap supports shown for key nodes. (PDF) [file pone.0111895.s001.pdf]
