# Supplementary material for: Independent Transitions between Monsoonal and Arid Biomes Revealed by Systematic Revison of a Complex of Australian Geckos (Diplodactylus; Diplodactylidae)
Source: PLoS One. 2014 Dec 10;9(12):e111895. doi: 10.1371/journal.pone.0111895 (PMC4262211; doi:10.1371/journal.pone.0111895)
Supplement: S1 Table — B. Intra-specific measures of genetic diversity and divergence. C. Diversity and demographic summary statistics. (DOCX) [file pone.0111895.s002.docx]

| **Table S1A. Measures of inter-specific diversity and divergence. Numbers above the diagonal refer to average number of uncorrected**  **nucleotide substitutions per site between populations.** | | | | | | | | | | | | | | | | |
| --- | --- | --- | --- | --- | --- | --- | --- | --- | --- | --- | --- | --- | --- | --- | --- | --- |
| **Numbers under the diagonal refer to average number of nucleotide substitutions per site between populations (Dxy) with Jukes & Cantor.** | | | | | | | | | | | | | | |  |  |
|  |  | |  | | | | | | |  | |  | |  |  |  |
|  | *D. barraganae* | | *D. hilli* | *D. bilybara* | | | *D. conspicillatus* | *D. custos* | *D. laevis* | | *D. platyurus* | |  |  |  |  |
|  |  | |  |  | | |  |  |  | |  | |  |  |  |  |
| *D. barraganae (N=1)* | _ | | 0.161 | 0.155 | | | 0.146 | 0.159 | 0.180 | | 0.199 | |  |  |  |  |
| *D. hilli (N=1)* | 0.181 | | _ | 0.136 | | | 0.177 | 0.150 | 0.148 | | 0.190 | |  |  |  |  |
| *D. bilybara (N=60)* | 0.173 | | 0.153 | _ | | | 0.160 | 0.149 | 0.129 | | 0.193 | |  |  |  |  |
| *D. conspicillatus (N=28)* | 0.162 | | 0.199 | 0.179 | | | _ | 0.168 | 0.173 | | 0.188 | |  |  |  |  |
| *D. custos (N=7)* | 0.177 | | 0.166 | 0.164 | | | 0.188 | _ | 0.144 | | 0.183 | |  |  |  |  |
| *D. laevis (N=62)* | 0.206 | | 0.163 | 0.149 | | | 0.198 | 0.158 | _ | | 0.200 | |  |  |  |  |
| *D. platyurus (N=11)* | 0.230 | | 0.218 | 0.221 | | | 0.217 | 0.208 | 0.235 | | _ | |  |  |  |  |
|  |  | |  |  | | |  |  |  | |  | |  |  |  |  |
|  |  | |  | | | | | | |  | |  | |  |  |  |
|  |  | |  | | | | | | |  | |  | |  |  |  |
|  |  | |  | | | | | | |  | |  | |  |  |  |
|  |  | |  | | | | | | |  | |  | |  |  |  |
| **Table S1B. Intra-specific measures of diversity and divergence.** | | | | | | | | | |  | |  | |  |  |  |
|  | |  | | |  | | | | |  | |  | |  |  |  |
| **Taxon** | | **mtDNA uncorrected P distance** | | | | **mtDNA corrected JC diversity (Pi)** | | | |  | |  | |  |  |  |
|  | |  | | | |  | | | |  | |  | |  |  |  |
| *D. barraganae* (N=1) | | 0 | | | | 0 | | | |  | |  | |  |  |  |
| *D. hillii* (N=1) | | 0 | | | | 0 | | | |  | |  | |  |  |  |
| *D. bilybara* (N=60) | | 0.014 (0-0.029) | | | | 0.013 (0.00-0.027) | | | |  | |  | |  |  |  |
| *D. conspicillatus* (N=28) | | 0.066 (0-0.119) | | | | 0.071 (0.0-0.133) | | | |  | |  | |  |  |  |
| *D. custos* (N=7) | | 0.028 (0-0.041) | | | | 0.027 (0.003-0.043) | | | |  | |  | |  |  |  |
| *D. laevis* (N=62) | | 0.040 (0-0.067) | | | | 0.040 (0.0-0.076) | | | |  | |  | |  |  |  |
| *D. platyurus* (N=11) | | 0.086 (0-0.134) | | | | 0.094 (0.0-0.159) | | | |  | |  | |  |  |  |
|  | |  | | |  | | | | |  | |  | |  |  |  |
|  | |  | | |  | | | | |  | |  | |  |  |  |
|  | |  | | |  | | | | |  | |  | |  |  |  |
|  | |  | | |  | | | | |  | |  | |  |  |  |
|  | |  | | |  | | | | |  | |  | |  |  |  |
| **Table S1C. Diversity and demographic summary statistics. Bold labels with a * indicate p value significance at the 5% level.** | | | | | | | | | | | | | |  |  |  |
|  | |  | | |  | | | | |  | |  | |  |  |  |
|  | | *Fu's S* | | | *Tajoma's D* | | | | | *R2* | |  | |  |  |  |
|  | |  | | |  | | | | |  | |  | |  |  |  |
| *D. barraganae (N=1)* | | na | | | na | | | | | na | |  | |  |  |  |
| *D. hilli (N=1)* | | na | | | na | | | | | na | |  | |  |  |  |
| *D. bilybara (N=60)* | | **-33.041*** | | | **-1.48949*** | | | | | **0.0553*** | |  | |  |  |  |
| *D. conspicillatus (N=28)* | | -2.55 | | | -0.45277 | | | | | 0.1159 | |  | |  |  |  |
| *D. custos (N=7)* | | -0.173 | | | -0.13574 | | | | | 0.1559 | |  | |  |  |  |
| *D. laevis (N=62)* | | **-14.307*** | | | -0.69946 | | | | | 0.0877 | |  | |  |  |  |
| *D. platyurus (N=11)* | | 3.45 | | | 1.1541 | | | | | 0.2188 | |  | |  |  |  |
|  | |  | | |  | | | | |  | |  | |  |  |  |
