# Supplementary material for: Independent Transitions between Monsoonal and Arid Biomes Revealed by Systematic Revison of a Complex of Australian Geckos (Diplodactylus; Diplodactylidae)
Source: PLoS One. 2014 Dec 10;9(12):e111895. doi: 10.1371/journal.pone.0111895 (PMC4262211; doi:10.1371/journal.pone.0111895)
Supplement: S1 Appendix — (DOCX) [file pone.0111895.s003.docx]

**Appendix S1. Additional material examined**

***Diplodactylus conspicillatus***. **—** **Western Australian Museum:** WAM R85641, 8km WNW Point Salvation, (28° 12’ S, 123° 36’ E); WAM R85654, WAM R85663, WAM R85851, 39km E Laverton, (28° 28’ S, 122° 50’ E); WAM R110762, Jimblebar east, (23° 23’ 41" S, 120° 18’ 35" E); WAM R163305, Neale Junction Nature Reserve, (28° 41’18" S, 125° 50’54" E); WAM R163307, Neale Junction Nature Reserve, (28° 34’ 24" S, 125° 47’ 27" E); WAM R163309, Neale Junction Nature Reserve, (28° 17’ 56" S, 126° 06’13" E); WAM R166299, 16.8km ENE Blackstone, (25° 56’ 07" S, 128° 26’ 16" E). **South Australian Museum:** SAMA R20972, Olympic Dam area Roxby Downs (30° 23’ S, 136° 51’ E); SAMA R26512, Granite Downs Stn nr homestead (26° 57’ S, 133° 34’ E); SAMA R38782, 4km E of Tennant Creek, on Peko Rd (19° 40' S, 134° 14' E). **Northern Territory Museum:** NTM R14265, NTM R14274, Atartinga Station (22° 21’ S, 134° 11’ E); NTM R15362, Adjacent to Plains Lawrence Gorge Waterhouse Range (24° 00’ S, 133° 26' E); NTM R16794, Amungee M (16° 31’ S, 134° 19’ E) ; NTM R22129, Toko Ranges, Tobermorey Station (22° 44’ S, 137° 56’ E); NTM R23702, Barkly Tablelands (19° 36’ S, 135° 46’ E); NTM R23702, Barkly Tablelands (19° 36’ S, 135° 46’ E); NTM R25497, Jasper Gorge, Victoria River Downs (16° 01’ 55" S, 130° 45’ 44" E); NTM R25959, Jasper Gorge, Gregory National Park (16° 01’ 53" S, 130° 48’ 16" E); NTM R26945, Maryvale Station, Sturt Plateau (15° 49’ 13" S, 133° 14’ 10" E); NTM R32470, Dalmore Downs Station, No.5 Bore (19° 00’ 27" S, 135° 00’ 35" E). **Australian Museum:** AMS R73042, 13 Km N Dajarra on Boulia-Mt Isa Rd (21° 37’ S, 139° 35’ E). **Queensland Museum:** QM J6520, Mallon, 48km S Cloncurry (21° 05’S, 140° 18’E).

***Diplodactylus laevis***. **—** **Australian Museum:** AMS R11367*,* S.W.NSW (33° 28’ S, 144° 56’ E). **Western Australian Museum:** WAM R70919, 48km 250 deg Goorda Tower (18° 54’ 30”S, 23° 02’ E); WAM R85642, WAM R85647, WAM R169279, 8km WNW Point Salvation (28° 12’ S, 123° 36’ E); WAM R85646, WAM R85650, WAM R85852, 39km E Laverton (28° 28’ S, 122° 50’ E); WAM R154834, Goldsworthy-Shay Gap road (20°27’42"S, 120°20’36"E); WAM R155446, 7–8km WNW Point Salvation (28° 12’ S, 123° 35’ E); WAM R159826, 10km S Mallina Homestead (20°58’ 10"S, 118° 02’ 54"E); WAM R159846, 12.5km S Whim Creek Hotel (20° 56’ 59"S, 117° 50’ 59"E); WAM R159965, 47km NNE Whim Creek Hotel (20º 28’ 49” S, 118º 02’ 25” E); WAM R161444, 29km NNE Marble Bar (20º 56’ 07” S, 119º 51’ 29” E); WAM R166297, 5.6km SE Mount Ant (24° 48’ 02” S, 128° 47’ 50” E); WAM R166301, 18.6km NE Blackstone R166302, 3.2km N Pungkulpirri Waterhole (24° 37’ 43” S, 128° 45’ 20” E); WAM R166304, 4.2km SSE Pungkulpirri Waterhole (24° 41’ 37” S, 128° 45’ 46” E); WAM R163749, 12.5km NNE Goldsworthy (20°14’ 31"S, 119° 34’ 28"E); WAM R166479, 60km E Port Hedland (20° 22’ 11"S, 119° 38’ E); R168480, Coulomb Point (17° 34’ 25” S, 122° 10’ 10” E); WAM R172199, WAM R172201, Great Victoria Desert (28 ° 22’ 21” S, 127° 32’ 19” E). **Queensland Museum:** QM J85433, Cravens Peak, SW Boulia (23° 08’ 43"S, 138° 21’ 03").

***Diplodactylus platyurus*. —** **Queensland Museum:** QM J3315, Mungana, Chillagoe Line (17° 06’ S, 144° 23’ E); QM J36793, Ambathala NRS, 1km S Ra Tank (22° 19’ S, 146° 45’ E); QM J45805, Bluff, 1.8km E (23° 35’ S, 149° 06’ E); QM J47268, Bluff, 1.8km E (22° 19’ S, 146° 45’ E); QM J58905, Georgetown, 20.1 km W (18° 18’ S, 143° 22’ E); QM J59410, Normanton, 16.0km West of, on the Cloncurry Road (23° 31’ S, 148° 10’ E); QM J59978, Emerald Township (24° 07’ 40 S, 143° 11’ 30” E); QM J61172, Jundah.`Noonbah Stn' Rd to`Waterloo Stn' in Bore Paddock (18° 16’ 30” S, 144° 33’ 30” E); QM J61444, Fifteen Mile Ck, Undara Lava Tunnel (23° 40’ S, 148° 06’ E); QM J61452, Camp Fairbairn (23° 40’ S, 148° 06’ E); QM J62477, Camp Fairbairn (22° 09’ 06” S, 148° 04’ 42” E); QM J63086, Blair Athol Coal Mine (22° 42’ S, 147° 33’ E); QM J63087, Blair Athol Coal Mine (22° 42’ S, 147° 33’ E); QM J65100, Porcupine Gorge NP (22° 42’ S, 147° 33’ E); QM J66693–5, QM J69751, QM J69764, QM J69766, QM J70136 Moranbah, 5km S (22° 02’ S, 148° 03’ E); QM J68968, BHP S Walker Ck Coal Mine, 40km W Nebo (21° 44’ 36” S, 148° 24’ 50” E); QM J69438, Saltwater Ck (17° 48’ 30” S, 144° 24’ 30” E); QM J72336, Kalang (22° 32’ 11” S, 147° 01’ 22” E); QM J72600, Chillagoe (17° 08’ 25” S, 144° 31’ 30” E); QM J73018, Aramac area, within 100km radius (22° 58’ 30” S, 145° 14’ 30” E); QM J74549, Epping Forest NP (22° 22’ 05” S, 146° 40’ 44” E); QM J75973, Lochern NP (24° 01’ 48” S, 143° 16’ 12” E); QM J76965, Blair Athol Coal Mine (22° 42’ 31” S, 147° 31’ 06” E); QM J80628, Blackbraes NP, Emu Swamp Road (19° 23’ 30” S, 144° 09’ 09” E); QM J80726, Blackbraes NP, Gorge Creek Road (19° 33’ 51” S, 144° 04’ 21” E); QM J81308, Moranbah (21° 57’ 33.8’ S, 147° 53’ 23” E); QM J81323, Moranbah, 133km N (20° 50’ 53” S, 147° 45’ 57” E); QM J83467, Noonbah Station (24° 07’ S, 143°11’ E). QM J92286, Mingella Rd, near Townsville (19° 53’ S, 146° 38’ E); QM J92287, Winton (22° 28’ 41.5" S, 142° 53’ 31" E). **Australian Museum:** AM R10120, Clermont (22° 50’ S, 147° 38’ E); AM R.16635, Winton (22° 23’ S, 143° 02’ E); AMS R20363 – 64, Normanton (17° 40’ S, 141° 04’ E); AMS R110529, Scott's Tank, Diamantina Lakes, NW of Windorah (23° 58’ S, 141° 32’ E); AMS R110564, Camp 14 Km NE Scott's Tank, Diamantina Lakes, NW of Windorah (23° 45’ S,141° 40’ E); AMS R132997, Wanaaring, 4km W of Wanaaring at Turnoff To Wilcannia (29° 42’ S, 144° 07’ E); AMS R141988, Wanaaring (29° 42’ S, 144° 09’ E); AMS R143856, Stonehenge Area, Within 10km N to S of Stonehenge (24° 22’ S, 143° 19’ E); AMS R143905, 34.1km W Normanton P.O. Via Cloncurry Rd (17° 54’ 38” S, 140° 55’ 06” E); AMS R143907, 16.0km W Normanton P.O. Via Cloncurry Rd (17° 47’ 41” S, 141° 00’ 58” E); AMS R143909, 9.3km W Normanton P.O. Via Cloncurry Rd (141° 02’ 12” E, 17° 44’ 26” S; AMS R143911, 8.2km W Normanton P.O. Via Cloncurry Rd (17° 43’ 52” S, 141° 02’ 21” E); AMS R143914, 16.6km W Georgetown on Croydon Rd (18° 17’ 33” S, 143° 24’ 05” E); AMS R143915, 19.0km W Georgetown on Croydon Rd (18° 17’ 42” S, 143° 22’ 50” E); AMS R143916, 8.4km W Georgetown on Croydon Rd (18° 17’ 22” S, 143° 28’ 26” E); AMS R165659, R.165697, AMS R166837 Nocoleche Nature Reserve, 11km West of Wanaaring - Wilcannia Road (29° 52’ 08” S, 144° 00’ 34” E); AMS R165673, Lake Peery National Park (30° 43’ 28” S, 143° 29’ 15” E). **South Australian Museum** –SAMA R2032, Cairns (16° 55’ S, 145° 46’ E); SAMA R3822, Woodstock via Townsville (19° 36’ S, 146° 50’ E).

***Diplodactylus custos*** **sp. nov.—** **Western Australian Museum:** WAM R103463, Bungle Bungle National Park (17° 24’ S, 128°45’E); WAM R152717, Purnululu National Park (17° 23’21”S, 128°15’17” E)
