# Supplementary material for: Correction: Independent Transitions between Monsoonal and Arid Biomes Revealed by Systematic Revison of a Complex of Australian Geckos (Diplodactylus; Diplodactylidae)
Source: PLoS One. 2015 Apr 16;10(4):e0126682. doi: 10.1371/journal.pone.0126682 (PMC4399945; doi:10.1371/journal.pone.0126682)
Supplement: S2 File — (PDF) [file pone.0126682.s002.pdf]

RESEARCH ARTICLE

Independent Transitions between Monsoonal and Arid Biomes Revealed by Systematic Revison of a Complex of Australian Geckos (*Diplodactylus*; Diplodactylidae)

Paul M. Oliver[1,2,3]*, **Patrick J. Couper**[4], **Mitzy Pepper**[3]

1 Department of Zoology, University of Melbourne, Melbourne, Victoria, Australia, **2** Department of Sciences, Museum Victoria, Melbourne, Victoria, Australia, **3** Division of Evolution, Ecology & Genetics, Research School of Biology, The Australian National University, Canberra, Australian Capital Territory, Australia, **4** Queensland Museum, Brisbane, Queensland, Australia

* Paul.oliver@anu.edu.au

OPEN ACCESS

Citation: Oliver PM, Couper PJ, Pepper M (2014) Independent Transitions between Monsoonal and Arid Biomes Revealed by Systematic Revison of a Complex of Australian Geckos (*Diplodactylus*; Diplodactylidae). PLoS ONE 9(12): e111895. doi:10.1371/journal.pone.0111895

Academic Editor: Matthias Stöck, Leibniz-Institute of Freshwater Ecology and Inland Fisheries, GERMANY

Received: May 22, 2014

Accepted: September 5, 2014

Published: December 10, 2014

Copyright: © 2014 Oliver et al. This is an open access article distributed under the terms of the Creative Commons Attribution License, which permits unrestricted use, distribution, and reproduction in any medium, provided the original author and source are credited.

Data Availability Statement: All relevant data are within the paper and its Supporting Information files. Sequence data has been uploaded to GenBank and the accession numbers can be found in Table 1.

Funding: This work was supported by Mckenzie Postdoctoral Fellowship at the University of Melbourne to Paul Oliver, an Australian Research Council linage Grant to Paul Oliver, Mike Lee and Paul Doughty, an Australian Biological Resources Survey Grant to Mark Hutchinson, Mark Adams, Paul Oliver and Paul Doughty, and the Queensland Museum. Mitzy Pepper's contributions were

Abstract

How the widespread expansion and intensification of aridity through the Neogene has shaped the Austral biota is a major question in Antipodean biogeography. Lineages distributed across wide aridity gradients provide opportunities to examine the timing, frequency, and direction of transitions between arid and mesic regions. Here, we use molecular genetics and morphological data to investigate the systematics and biogeography of a nominal Australian gecko species (*Diplodactylus conspicillatus sensu lato*) with a wide distribution spanning most of the Australian Arid Zone (AAZ) and Monsoonal Tropics (AMT). Our data support a minimum of seven genetically distinct and morphologically diagnosable taxa; we thus redefine the type species, ressurrect three names from synonymy, and describe three new species. Our inferred phylogeny suggests the history and diversification of lineages in the AAZ and AMT are intimately linked, with evidence of multiple independent interchanges since the late Miocene. However, despite this shared history, related lineages in these two regions also show evidence of broadly contrasting intra-regional responses to aridification; vicarance and speciation in older and increasingly attenuated mesic regions, versus a more dynamic history including independent colonisations and recent range expansions in the younger AAZ.

Introduction

The extent and intensity of arid conditions in the Southern Hemisphere has increased through the late Neogene, and expansive deserts are now a prominent feature of most southern continents (Africa, Australia and South America) [1–4]. These generally young arid zones are characterised by low, unpredictable rainfall and strong seasonal variation in temperature, and this major climatic shift has had profound biological implications; some lineages have adapted to

supported by an Australian Research Council grant to J. Scott Keogh. The funders had no role in study design, data collection and analysis, decision to publish, or preparation of the manuscript.

Competing Interests: The authors have declared that no competing interests exist.

aridity, while many others have retreated into shrinking mesic refugia or simply gone extinct [5–10].

In the face of expanding and intensifying aridification, comparatively mesic environments adjacent to arid areas serve a number of important functions. Over longer timescales, peripheral semi-arid or seasonally arid areas may provide zones in which lineages can accumulate preadapations that mediate subsequent successful colonisation of more arid zones [11,12]. Over shorter timescales (for instance the Pleistocene glacial oscillations over the last 2.5 million years) peripheral habitats may also function as refugia during peaks of aridity, and subsequently a source of populations able to recolonize the arid zone when conditions ameliorate [13,14]. Radiations which now comprise multiple taxa widely distributed across arid and mesic areas provide powerful oppurtunities to compare lineage diversity across regions, and test the ideas about when, from where, and how, lineages colonised (or recolonised) nascent arid biomes [6,12,14–16].

The Australian Arid Zone (AAZ) and Australian Monsoonal Tropics (AMT) are two of the largest Australian biomes, and share a long border than spans most of northern Australia [9,17]. Many lineages occur across both regions, suggesting there has been significant evolutionary interchange, however only a small number of studies have examined the diversification of taxa spanning these two areas [14,18]. This work has generally supported the hypothesis that lineages in the AMT are characterised by higher taxonomic or genetic diversity and more narrow distributions (indicative of persistence and localised diversification), while the AAZ is inhabited by a smaller number of derived and often relatively widespread lineages (indicative of colonisation and range expansion) [14,16,18]. However the total number of studies remains few, and broader insight into how often, and when lineages diversified across these two biomes requires examination of additional lineages.

Squamates (lizards and snakes) are arguably the most successful vertebrate group in Australia [19,20] and are playing a key role in an emerging research program to understand how aridification has shaped patterns of evolution [12,21]. However, while the problem of cryptic diversity within Australian squamates was flagged over two decades ago [22], ongoing research and new methods continue to reveal high levels of unrecognised evolutionary diversity (e.g. [23,24]). Therefore it is not surprising that systematic work over the last two decades has also revealed numerous instances where overly conservative taxonomy has obscured macroevolutionary patterns such as long-term persistence, micro-endemism, inter-regional dispersal events, and morphological stasis (e.g. [6–8,14,15,25–27]).

Diplodactylus conspicillatus Lucas & Frost 1897 [28] is a small (svl <65mm), terrestrial Australian gecko with a distinctive short plump tail (commonly referred to as the Fat-tailed Gecko). As currently defined this species has one of the widest distributions of any Australian lizard, and occurs across most of the AAZ and AMT in a diverse range of habitats, including desert, low open shrubland, low shrubland, low open woodland, low woodland, woodland and tall shrubland, and on a wide range of substrates [29]. A published overview of phylogenetic diversity in the genus *Diplodactylus* based on mitochondrial DNA, allozymes and karyotypes revealed nine divergent clades within the *D. conspicillatus* complex [30]. Although many of these were represented by few samples, each was recognised as a 'candidate species', pending further morphological and molecular work. Pepper *et al.* [24] subsequently presented a more comprehensive sampling of *D. conspicillatus sensu lato* in Western Australia, and also found evidence of deep divergences, including two near parapatric lineages in the Pilbara region.

Here we use an expanded mitochondrial dataset along with detailed morphological examination to assess how lineages of the *D. conspicillatus* complex have diversified across the AAZ and AMT. Specifically we a) contrast genetic diversity in the two regions, and b) use simple ancestral state reconstruction to assess the frequency and trajectory of shifts between biomes.

Based on these data we also present a revised taxonomy, formally recognizing seven of the lineages identified by Oliver et al. [29] as species (redefined *Diplodactylus conspicillatus sensu stricto*, resurrected *Diplodactylus hillii*, *D. laevis and D. platyurus* and three newly described species) and thereby add six further species to the diverse Australian lizard fauna.

Methods

Material examined

This study utilised specimens and tissues held in the Australian Museum (AMS), National Museum of Victoria (NMV), Northern Territory Museum and Art Gallery (NTM), Queensland Museum (QM), South Australian Museum (SAMA) and Western Australian Museum (WAM). Where possible, specimens included in genetic analyses were also included in morphological analyses. Tissue samples from nominated holotypes for all three newly described taxa were included in assessments of genetic diversity.

Morphological characterisation of the types of *D. conspicillatus* and its synonyms (as listed in Cogger *et al*., 1983), *D. hillii*, *Gymnodactylus laevis* and *D. platyurus*, was made by direct examination (*D. conspicillatus* [NMV] and *D. hillii* [QM]) or using photographic images generously provided by Dr H.G. Cogger (*Gymnodactylus laevis* [Naturmuseum Senckenberg, Frankfurt] and *D. platyurus* [British Museum of Natural History, London]).

Genetic data

Genetic analyses included mitochondrial data from 169 specimens of *Diplodactylus conspicillatus sensu lato* (see Table 1 for specimen and locality information). DNA extraction and sequencing protocols for most samples are detailed elsewhere [25,31]. DNA from new samples was extracted using a Qiagen high throughput extraction robot at Museum Victoria. A $\sim$1200 base pair (bp) region of the *ND2* gene and surrounding tRNAs was amplified using one of the following two combinations of primers: 1) AAG CTT TCG GGG CCC ATA CC (L4437) [32] and CTA AAA TRT TRC GGG ATC GAG GCC (Asn-tRNA) [33]; or 2) GCC CAT ACC CCG AAA ATS TTG and TTA GGGTRG TTA TTT GHG AYA TKC G [25]. PCR products were amplified for 40 cycles at an annealing temperature of 55°C. Unpurified PCR products were sent to a genetic services company (Macrogen, Korea) and sequenced in both directions using Sanger sequencing technologies.

New sequences generated in this study were aligned with data presented by Oliver et al. [30] and Pepper et al. [24]. Alignment of sequences was first performed automatically using the software MUSCLE [34], then refined by eye in Se-Al [35]. We translated nucleotide data into amino acid sequences and checked the alignment for internal stop codons and frame-shift mutations. Our final edited alignment included up to 1054 characters. We used the unlinked branch lengths and BIC settings in PartitionFinder [36] to determine the best partitioning strategy and model of nucleotide substitution (GTR+I+G, with all codon positions considered together in a single partition).

Phylogenetic analyses

Phylogenetic relationships were estimated using standard Maximum Likelihood (RAxML v7.2.8) [37] and Bayesian techniques (BEAST v1.8.0) [38]. All unique samples were included in initial analyses (S1 Fig.), however for subsequent phylogenetic analyses we focused on a reduced subset of data from which a number of identical or near identical sequences for the two most extensively sampled major clades were removed. Maximum Likelihood analyses were run using the default settings for RAxML on the CIPRES portal; the GTR+G model of sequence

Table 1. Museum Voucher and locality details of all specimens included in phylogenetic analyses.

Museum Number	Species	Locality	Latitude (dec.)	Longitude (dec.)	Genbank #
WAMR157640	*conspicillatus*	Newman, WA	-23.3097	119.7569	KM267082
SAMAR20884	*conspicillatus*	Olympic Dam area, Roxby Downs, SA	-30.3833	136.8833	FJ665543
SAMAR45256	*conspicillatus*	Salt Ck Cross E L Gairdner, SA	-31.5500	136.3500	FJ665541
SAMAR51587	*conspicillatus*	Amata, SA	-26.2828	131.4867	FJ665542
WAMR110770	*conspicillatus*	Jimblebar East, WA	-23.4406	120.3333	JX946871
WAMR110769	*conspicillatus*	Jimblebar East, WA	-23.3656	120.3211	JX946870
WAMR110762	*conspicillatus*	Jimblebar East, WA	-23.3947	120.3097	JX946873
WAMR110767	*conspicillatus*	Jimblebar East, WA	-23.3961	120.3100	JX946874
SAMAR46981	*conspicillatus*	Mosquito Camp Dam, SA	-26.1578	134.5136	FJ665547
SAMAR26512	*conspicillatus*	Granite Downs Station, WA	-26.9500	133.5667	FJ665545
SAMAR26513	*conspicillatus*	Granite Downs Station, WA	-26.9500	133.5667	FJ665544
SAMAR51514	*conspicillatus*	3.3k SW Indulkana, SA	-26.9800	133.2700	FJ665546
WAMR136643	*conspicillatus*	Lake Mason Station, WA	-27.6975	119.2800	KM267080
WAMR136647	*conspicillatus*	Lake Mason Station, WA	-27.7141	119.5311	KM267081
WAMR97324	*conspicillatus*	Mount Windarra, WA	-28.4583	122.2417	JX946799
WAMR144640	*conspicillatus*	Kalgoorlie, WA	-30.2014	120.9742	JX946847
SAMAR42574	*conspicillatus*	168 km NE of Emu, SA	-28.2333	133.3333	FJ665539
SAMAR32133	*conspicillatus*	Maralinga, SA	-30.2503	131.5458	FJ665538
SAMAR62135	*conspicillatus*	18.4k NE Blackstone, WA	-25.8917	128.4269	FJ665520
SAMAR62106	*conspicillatus*	Morgan Range, 16.8k ENE Blackstone, WA	-25.9353	128.4378	FJ665523
SAMAR62107	*conspicillatus*	Morgan Range, 16.8k ENE Blackstone, WA	-25.9353	128.4378	FJ665522
WAMR166299	*conspicillatus*	Morgan Range, 16.8k ENE Blackstone, WA	-25.9353	128.4378	FJ665540
QMJ92288	*conspicillatus*	Mt Isa, QLD	-21.1300	139.2500	FJ665534
AMSR125042	*conspicillatus*	Cunnamulla, QLD	-28.0667	145.6833	FJ665533
NTMR15362	*conspicillatus*	Lawrence Gorge Waterhouse Range, NT	-24.0200	133.4000	FJ665535
SAMAR38849	*conspicillatus*	Namatjira/Larapinta Drive Junction, SA	-26.7667	133.4500	FJ665536
NTMR35949	*conspicillatus*	120km East of Argadargada, NT	-21.2928	137.4100	KM267074
SAMAR38782	*conspicillatus*	Tennant Creek, NT	-19.6667	134.2333	FJ665537
SAMARR38819	*conspicillatus*	Three Ways, NT	-19.4333	134.2167	EF681786
NTMR24076	*conspicillatus*	Arafura Swamp Arnhem Land, NT	-12.5300	134.9000	FJ665532
WAMR110769	*conspicillatus*	Jimblebar East, WA	-23.3656	120.3211	KM267078
WAMR110770	*conspicillatus*	Jimblebar East, WA	-23.4406	120.3333	KM267079
WAMR110762	*conspicillatus*	Jimblebar East, WA	-23.3947	120.3097	KM267076
WAMR110767	*conspicillatus*	Jimblebar East, WA	-23.3961	120.3100	KM267077
WAMR97324	*conspicillatus*	Mount Windarra, WA	-28.4583	122.2417	KM267075
WAMR144640	*conspicillatus*	Kalgoorlie, WA	-30.2014	120.9742	KM267083
NTMR21395	*barraganae*	Mussellbrook, QLD	-18.6083	137.9883	FJ665515
NTMR17871	*hillii*	Corroboree Taven, NT	-12.7500	131.4833	EF681785
WAMR162453	*custos*	West Kununurra, WA	-15.7700	128.6700	JX946794
WAMR172853	*custos*	Ellenbrae Station, WA	-15.9839	127.0539	KM267051
WAMR164780	*custos*	The Grotto, WA	-15.7178	128.2597	KM267049
WAMR132713	*custos*	Wyndham, WA	-15.7119	128.2656	JX946822
WAMR172916	*custos*	Doongan Station, WA	15.2290	125.2084	KM267052
WAMR172675	*custos*	Irvine Island, WA	16.3353	124.0528	KM267050
CM1800	*custos*	Adcock Gorge, WA	-16.9267	125.7795	KM267048
AMSR143914?	*platyurus*	16.6km W Georgetown on Croydon Rd, QLD	-18.2925	143.4014	FJ665514

(*Continued*)

Table 1. (*Continued*)

Museum Number	Species	Locality	Latitude (dec.)	Longitude (dec.)	Genbank #
AMSR143909	*platyurus*	9.3km W Normanton P.O via Cloncurry Rd, QLD	-17.7300	141.0300	FJ665512
AMSR143911	*platyurus*	8.2km W Normanton P.O via Cloncurry Rd, QLD	-17.7300	141.0300	FJ665511
AMSR143916	*platyurus*	8.4km W of Georgetown on Croydon Rd, QLD	-18.2800	143.4700	FJ665513
QMJ92887	*platyurus*	Winton, QLD	-22.4700	142.9200	FJ665530
SAMAR63336	*platyurus*	Winton, QLD	-22.4500	142.9500	FJ665531
ABTC31900	*platyurus*	Noonbah Station, QLD	-24.1200	143.1800	FJ665528
AMSR143856	*platyurus*	Stonehenge area, QLD	-24.3700	143.3200	FJ665527
AMSR158426	*platyurus*	Sturt National Park, NSW	-29.3700	142.0300	FJ66552
QMJ92287	*platyurus*	Mingella, QLD	-19.8700	146.6300	FJ665526
SAMAR63337	*platyurus*	Mingella, QLD	-19.8700	146.6300	FJ665525
WAMR135321	*bilybara*	Cape Lambert, WA	-20.7544	117.0811	JX946823
WAMR140334	*bilybara*	Millstream-Chichester National Park, WA	-21.4619	117.1625	JX946838
WAMR132531	*bilybara*	Burrup Peninsula, WA	-20.6767	116.7522	JX946814
WAMR132529	*bilybara*	Burrup Peninsula, WA	-20.6803	116.7436	JX946813
WAMR112197	*bilybara*	Onslow Area, WA	-21.6758	115.1458	KM267032
WAMR102917	*bilybara*	Cane River, WA	-22.1992	115.5486	JX946835
WAMR174500	*bilybara*	Barradale, WA	-22.9167	114.7667	FJ665517
WAMR158333	*bilybara*	Giralia Homestead, WA	-22.6939	114.3911	JX946861
WAMR157275	*bilybara*	Yanrey Station, WA	-22.2675	114.5228	JX946865
WAMR157302	*bilybara*	Yanrey Station, WA	-22.1578	114.5286	JX946867
WAMR126821	*bilybara*	Bush Bay, WA	-25.1258	113.8228	KM267035
WAMR120671	*bilybara*	Mardathuna Homestead, WA	-24.4069	114.4731	JX946795
WAMR127701	*bilybara*	Mount Tom Price Mine, WA	-22.8111	117.7675	JX946796
WAMR157533	*bilybara*	Robe River, WA	-21.7478	116.0753	JX946856
WAMR119206	*bilybara*	Carey Downs Homestead, WA	-25.5333	115.4667	JX946787
WAMR165713	*bilybara*	Jack Hills, WA	-26.0567	117.2161	JX946876
WAMR141359	*bilybara*	Cape Preston Area, WA	-21.0164	116.1956	JX946843
WAMR145193	*bilybara*	Learmonth Airstrip, WA	-22.2431	114.0347	JX946848
WAMR102503	*bilybara*	Barlee Range Nature Reserve, WA	-23.1017	116.0078	JX946784
WAMR126769	*bilybara*	Mardathuna Homestead, WA	-24.5114	114.6367	JX946773
WAMR126770	*bilybara*	Boolathana Homestead, WA	-24.4131	113.6631	JX946774
WAMR126821	*bilybara*	Bush Bay, WA	-25.1258	113.8228	JX946775
WAMR126827	*bilybara*	Boolathana Homestead, WA	-24.4131	113.7067	JX946776
WAMR158331	*bilybara*	Giralia Homestead, WA	-22.6939	114.3911	JX946860
WAMR158333	*bilybara*	Giralia Homestead, WA	-22.6939	114.3911	KM267045
WAMR158335	*bilybara*	Giralia Homestead, WA	-22.6939	114.3911	JX946862
WAMR158349	*bilybara*	Giralia Station, WA	-22.7844	114.2792	JX946863
WAMR157275	*bilybara*	Yanrey Station, WA	-22.2675	114.5228	KM267042
WAMR157276	*bilybara*	Yanrey Station, WA	-22.2997	114.5931	JX946866
WAMR157302	*bilybara*	Yanrey Station, WA	-22.1578	114.5286	KM267043
WAMR126859	*bilybara*	Bush Bay, WA	-25.1258	113.8228	JX946777
WAMR156243	*bilybara*	Onslow Area, WA	-21.6886	115.0925	JX946872
WAMR151061	*bilybara*	Carnarvon, WA	-24.8833	113.7667	JX946875
WAMR165713	*bilybara*	Jack Hills, WA	-26.0567	117.2161	KM267046
WAMR102477	*bilybara*	Barlee Range Nature Reserve, WA	-23.0447	115.7872	JX946781
WAMR102478	*bilybara*	Barlee Range Nature Reserve, WA	-23.0447	115.7872	JX946782
WAMR102480	*bilybara*	Barlee Range Nature Reserve, WA	-23.0447	115.7872	JX946783

(*Continued*)

Table 1. (*Continued*)

Museum Number	Species	Locality	Latitude (dec.)	Longitude (dec.)	Genbank #
WAMR102503	*bilybara*	Barlee Range Nature Reserve, WA	-23.1017	116.0078	KM267030
WAMR123119	*bilybara*	Bush Bay, WA	-25.1258	113.8228	JX946785
WAMR122888	*bilybara*	Mardathuna Homestead, WA	-24.4069	114.4731	JX946786
WAMR119206	*bilybara*	Carey Downs Homestead, WA	-25.5333	115.4667	KM267033
WAMR127498	*bilybara*	Onslow, WA	-21.7333	115.0833	JX946788
WAMR127520	*bilybara*	Onslow, WA	-21.7333	115.0833	JX946789
WAMR120700	*bilybara*	Boolathana Homestead, WA	-24.4131	113.7067	JX946791
WAMR120671	*bilybara*	Mardathuna Homestead, WA	-24.4069	114.4731	KM267034
WAMR127701	*bilybara*	Mount Tom Price Mine, WA	-22.8111	117.7675	KM267036
WAMR132529	*bilybara*	Burrup Peninsula, WA	-20.6803	116.7436	KM267037
WAMR132531	*bilybara*	Burrup Peninsula, WA	-20.6767	116.7522	KM267038
WAMR132532	*bilybara*	Burrup Peninsula, WA	-20.6767	116.7522	JX946815
WAMR135321	*bilybara*	Cape Lambert, WA	-20.7544	117.0811	KM267039
WAMR135378	*bilybara*	Mt Brockman, WA	-22.4197	117.4300	JX946824
WAMR135406	*bilybara*	Mt Brockman, WA	-22.3500	117.3500	JX946825
WAMR135408	*bilybara*	Mt Brockman, WA	-22.4197	117.4300	JX946826
WAMR135431	*bilybara*	Mt Brockman, WA	-22.4197	117.4300	JX946827
WAMR135455	*bilybara*	Mount Brockman Area, WA	-22.4667	117.3000	JX946828
WAMR113620	*bilybara*	Nanutarra, WA	-22.4167	115.6167	JX946830
WAMR113621	*bilybara*	Nanutarra, WA	-22.4167	115.6167	JX946831
WAMR113635	*bilybara*	Nanutarra, WA	-22.4167	115.6167	JX946832
WAMR102917	*bilybara*	Cane River, WA	-22.1992	115.5486	KM267031
WAMR102945	*bilybara*	Cane River, WA	-22.1992	115.5486	JX946836
WAMR102946	*bilybara*	Cane River, WA	-22.1992	115.5486	JX946837
WAMR140334	*bilybara*	Millstream-Chichester National Park, WA	-21.4619	117.1625	JX946838
WAMR140980	*bilybara*	Urala Station, WA	-21.7767	114.8719	JX946841
WAMR141359	*bilybara*	Cape Preston Area, WA	-21.0164	116.1956	KM267041
WAMR139168	*bilybara*	Burrup Peninsula, WA	-20.6500	116.7667	JX946844
WAMR145193	*bilybara*	Learmonth Airstrip, WA	-22.2431	114.0347	KM267047
WAMR112197	*bilybara*	Onslow Area, WA	-21.6758	115.1458	JX946850
WAMR157533	*bilybara*	Robe River, WA	-21.7478	116.0753	KM267044
WAMR158325	*bilybara*	Giralia Homestead, WA	-22.6939	114.3911	JX946857
WAMR158327	*bilybara*	Giralia Homestead, WA	-22.6939	114.3911	JX946858
WAMR158330	*bilybara*	Giralia Homestead, WA	-22.6939	114.3911	JX946859
SAMAR49077	*laevis*	1.7k NE Candradecka Dam, SA	-27.2000	140.8700	FJ665550
SAMAR49081	*laevis*	1.7k NE Candradecka Dam, SA	-27.2000	140.8700	FJ665549
SAMAR29936	*laevis*	Curtin Springs, NT	-25.5200	131.8200	FJ665544
WAMR166303	*laevis*	3.2k N Pungkulpirri Waterhole, Walter James Range, WA	-24.6286	128.7556	FJ665551
SAMAR36126	*laevis*	Yulara, NT	-25.2300	131.0200	FJ665553
WAMR111703	*laevis*	Wheelara Hill, WA	-23.3644	120.3450	KM267059
WAMR161577	*laevis*	Port Hedland, WA	-20.3464	118.89301	KM267069
WAMR161601	*laevis*	Goldsworthy, WA	-20.2419	119.5740	KM267070
WAMR157018	*laevis*	Fortescue Marsh, WA	-22.4592	119.0364	KM267067
WAMR157131	*laevis*	Roy Hill, WA	-22.6542	120.4097	JX946864
WAMR168480	*laevis*	Coulomb Point, WA	-17.5736	122.1694	KM267072
WAMR132176	*laevis*	Nifty Mine, WA	-21.6667	121.5833	KM267062
WAMR139264	*laevis*	Meentheena, WA	-21.2869	120.4594	JX946845

(*Continued*)

Table 1. (*Continued*)

Museum Number	Species	Locality	Latitude (dec.)	Longitude (dec.)	Genbank #
WAMR145516	*laevis*	Port Hedland, WA	-21.0600	118.7500	JX946849
WAMR102048	*laevis*	Strelley Homestead, WA	-20.3667	119.0167	JX946801
WAMR104056	*laevis*	Woodstock, WA	-21.6706	119.0417	JX946807
WAMR102054	*laevis*	Mundabullangana, WA	-20.7500	118.2500	JX946802
ABTC60729	*laevis*	30km SW Sangster's Bore, NT	-21.0200	130.1300	FJ665554
WAMR114921	*laevis*	Capricorn Roadhouse, WA	-23.7167	119.7167	JX946779
WAMR166625	*laevis*	Mons Cupri Mine, WA	-20.8664	117.8219	JX946877
WAMR161868	*laevis*	Marble Bar, WA	-21.4264	119.5530	KM267071
WAMR137010	*laevis*	Wanjarri Nature Reserve, WA	-27.3500	120.7667	JX946833
WAMR119777	*laevis*	Tehan Rockface, WA	-27.0333	124.7833	JX946790
WAMR110626	*laevis*	Tanami Desert, WA	-19.8944	128.8339	JX946868
WAMR94956	*laevis*	Well 39, Canning Stock Route, WA	-21.7833	125.6500	JX946798
WAMR102637	*laevis*	Little Sandy Desert, WA	-24.0753	120.3606	JX946780
ABTC41350	*laevis*	134km ENE Laverton, WA	-28.1700	123.6700	FJ665547
WAMR157131	*laevis*	Roy Hill, WA	-22.6542	120.4097	KM267068
WAMR110626	*laevis*	Tanami Desert, WA	-19.8944	128.8339	KM267058
WAMR110758	*laevis*	Jimblebar East, WA	-23.3656	120.3303	JX946869
WAMR166625	*laevis*	Mons Cupri Mine, WA	-20.8664	117.8219	KM267073
WAMR166480	*laevis*	Port Hedland, WA	-20.3697	119.6333	JX946879
WAMR113039	*laevis*	Lesley Salt Works, WA	-20.2833	118.8944	JX946778
WAMR114921	*laevis*	Capricorn Roadhouse, WA	-23.7167	119.7167	KM267060
WAMR102637	*laevis*	Little Sandy Desert, WA	-24.0753	120.3606	KM267056
WAMR119777	*laevis*	Tehan Rockface, WA	-27.0333	124.7833	KM267061
WAMR119956	*laevis*	Woodie Woodie Mine, WA	-21.6667	121.5833	JX946792
WAMR157395	*laevis*	Tanami Desert, WA	-19.6647	128.8858	JX946793
WAMR90895	*laevis*	Woodstock, WA	-21.6117	118.9556	JX946797
WAMR94956	*laevis*	Well 39, Canning Stock Route, WA	-21.7833	125.6500	KM267053
WAMR99143	*laevis*	Woodstock Station, WA	-21.6097	118.9622	JX946800
WAMR102048	*laevis*	Strelley Homestead, WA	-20.3667	119.0167	KM267054
WAMR102054	*laevis*	Mundabullangana, WA	-20.7500	118.2500	KM267055
WAMR104021	*laevis*	Woodstock, WA	-21.6117	118.9556	JX946803
WAMR104047	*laevis*	Woodstock, WA	-21.6117	118.9556	JX946804
WAMR104050	*laevis*	Woodstock, WA	-21.6092	118.9742	JX946805
WAMR104051	*laevis*	Woodstock, WA	-21.6092	118.9742	JX946806
WAMR104056	*laevis*	Woodstock, WA	-21.6706	119.0417	KM267057
WAMR104061	*laevis*	Woodstock, WA	-21.6092	118.9742	JX946808
WAMR104158	*laevis*	Woodstock, WA	-21.6092	118.9742	JX946809
WAMR108856	*laevis*	Mount Spinifex, WA	-20.7833	118.1167	JX946810
WAMR113068	*laevis*	Lesley Salt Works, WA	-20.3194	118.9000	JX946811
WAMR104059	*laevis*	Woodstock, WA	-21.6094	118.9619	JX946812
WAMR132546	*laevis*	Degrey River Station, WA	-20.2803	119.2019	JX946816
WAMR132175	*laevis*	Nifty Mine, WA	-21.6667	121.5833	JX946817
WAMR132176	*laevis*	Nifty Mine, WA	-21.6667	121.5833	JX946818
WAMR132177	*laevis*	Nifty Mine, WA	-21.6667	121.5833	JX946819
WAMR132178	*laevis*	Nifty Mine, WA	-21.6667	121.5833	JX946820
WAMR132689	*laevis*	Shay Gap, WA	-20.5778	120.3331	JX946821
WAMR113612	*laevis*	Newman, WA	-23.0175	119.8906	JX946829

(*Continued*)

Table 1. (*Continued*)

Museum Number	Species	Locality	Latitude (dec.)	Longitude (dec.)	Genbank #
WAMR137010	*laevis*	Wanjarri Nature Reserve, WA	-27.3500	120.7667	KM267064
WAMR138110	*laevis*	Nifty Copper Mine, WA	-21.6667	121.5833	JX946834
WAMR140708	*laevis*	Hope Downs, WA	-22.7328	119.4086	JX946839
WAMR140712	*laevis*	Hope Downs, WA	-22.8369	119.3758	JX946840
WAMR139020	*laevis*	Mandora, WA	-19.8122	121.4736	JX946842
WAMR139264	*laevis*	Meentheena, WA	-21.2869	120.4594	KM267065
WAMR139283	*laevis*	Meentheena, WA	-21.2900	120.4664	JX946846
WAMR145516	*laevis*	Port Hedland, WA	-21.0600	118.7500	KM267066
WAMR153889	*laevis*	WeeliWolli Creeck, WA	-22.8208	119.2869	JX946851
WAMR157003	*laevis*	Fortescue Marsh, WA	-22.4144	119.0067	JX946852
WAMR157018	*laevis*	Fortescue Marsh, WA	-22.4592	119.0364	JX946853
WAMR157024	*laevis*	Fortescue Marsh, WA	-22.4592	119.0364	JX946854
WAMR157500	*laevis*	Fortescue Marsh, WA	-22.4592	119.0364	JX946855

doi:10.1371/journal.pone.0111895.t001

evolution (as preferred by Stamatakis, [37]), and ceasing bootstrapping when MRE-bootstrapping criteria had been reached.

Bayesian analyses in BEAST used models and partitions as suggested by Partitionfinder, the Yule speciation prior (appropriate for analyses including relatively divergent lineages) and a relaxed log-normal clock and with model and partitions applied as above. After initial experimentation with settings and sampling, the final MCMC chains were run for 50 million generations, sampling every 50,000 steps. We estimated a timeframe of divergence using a 3% mean rate of pairwise sequence divergence (with a range between 1–4%) per one million years (see Oliver et al. [16] for justification). Tracer v1.5 [39] was used to confirm stability of parameter estimates and adequate mixing of the MCMC chains, and determine appropriate burn-in and acceptable effective sample sizes ($>$ 200). Maximum clade credibility trees, after exclusion of the first ten million generations (20%), were summarized with TreeAnnotator v1.7.2 [38].

Biome evolution

Ancestral state analyses were coestimated with topology and divergence dates in BEAST. To assess the number and nature of transitions between biomes, each node was coded as to whether the corresponding specimen was from within (1) or outside (0) the AAZ (defined here by a moisture index [mean annual rainfall divided by evaporation] of less than 0.4 [9]; Fig. 1, dotted line). The biome state of all outgroups was coded as ambiguous because: a) basal relationships within *Diplodactylus* were unresolved, and b) some of these taxa occur in the temperate biome, while this study is focused on transitions between the AMT and AAZ. The pattern of biome evolution was estimated using a simple substitution model for binary data which assumes equal probabilities for transitions between all states [40].

Population genetics

To infer past demographic fluctuations in response to the climate cycles of the Pleistocene, we calculated nucleotide diversity and tested for population size change using the basic population genetic measurements of Tajima's *D* [41], Fu's *Fs* [42] and R_2 [43] as implemented in DnaSP v. 5.0 [44]. Estimates and significance were calculated with 1000 coalescent simulations against the null hypothesis of a constant population size model, for each population corresponding to

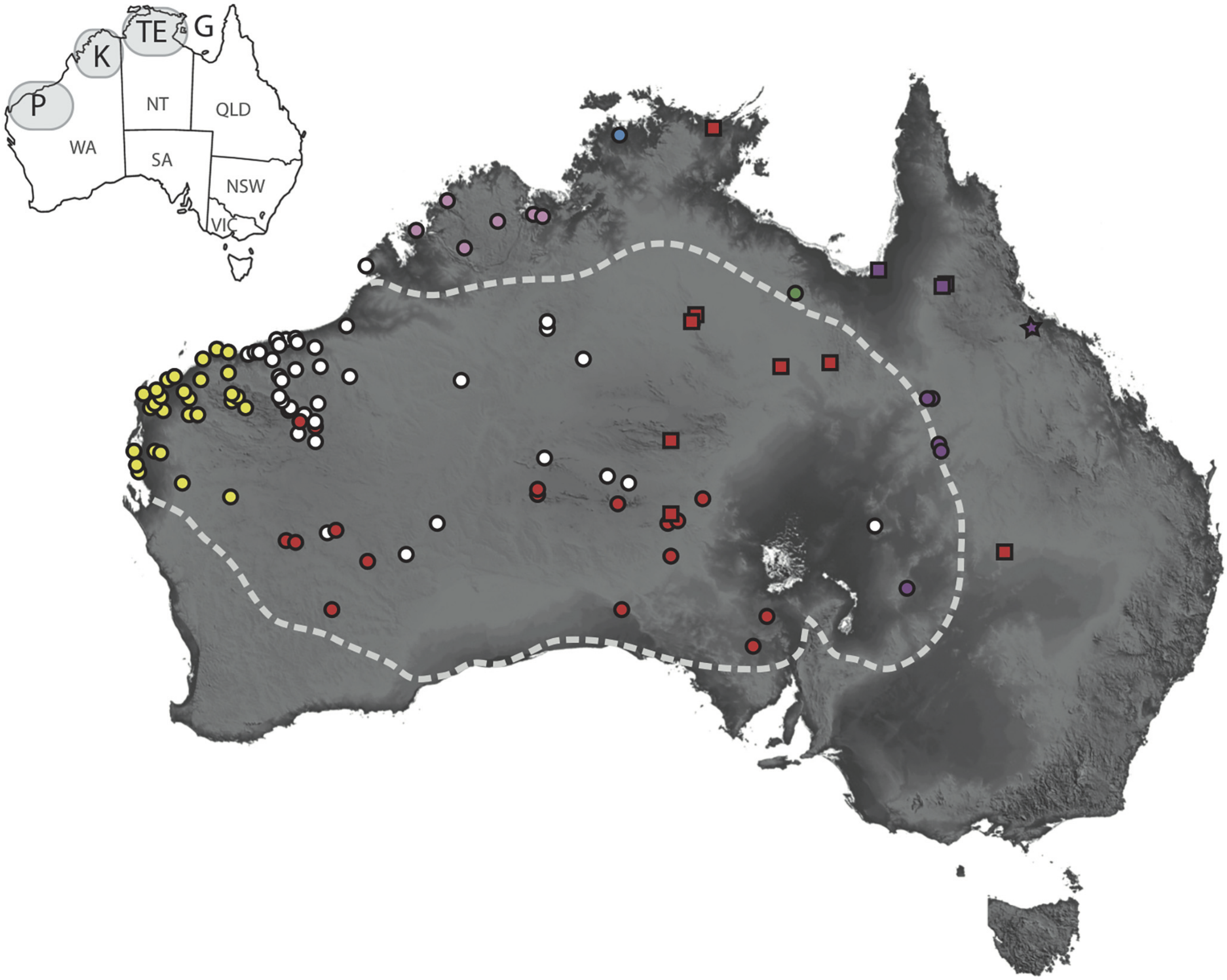

Fig 1. Distribution of genetically sampled individuals for major genetic lineages in the *D. conspicillatus* complex. Pink = *D. custos* **sp. nov**., Blue = *D. hillii*, Green = *D. barraganae* **sp. nov**., Purple = *D. platyurus* (circle = lineage F, star = lineage G, squares = lineage H), Red = *D. conspicillatus* (squares = northern lineage, circles = southern lineage), White = *D. laevis*, Yellow = *D. bilybara* **sp. nov**. The dashed line corresponds to the transition between regions with a moisture index (mean annual rainfall divided by evaporation) less than 0.4 (arid) to greater than 0.4 (semiarid to mesic) and is widely used as an approximate boundary of the Australian Arid Zone. Inset map on the top left corner indicates putative regions of endemism mentioned in the text; P = Pilbara, K = Kimberley, TE = "Top End", G = Gulf country.

doi:10.1371/journal.pone.0111895.g001

the phylogenetically distinct groups based on the mtDNA gene tree. As these historical inferences were based on data from the mitochondrial genome, they should be regarded as a preliminary framework requiring corroboration with appropriate nuclear loci. For major clades intra and inter-specific genetic distances were estimated using Arlequin v. 3.11 [45].

Morphometrics

All measurements (except SVL) and bilateral counts were recorded from the left side. The following measurements were taken using Mitutoyo electronic callipers: snout to vent length (SVL), tip of snout to anterior margin of cloaca with body straightened; tail length (T), from posterior margin of cloaca to tip of tail; tail width (TW), widest point across original tail; head

length (HL), mid anterior margin of ear to tip of snout; head width (HW), widest point of head, usually corresponding with, or slightly posterior to, position of ear opening; head depth (HD), lower jaw to top of head at mid orbit; snout length (S), tip of snout to anterior margin of orbit; eye to ear (EE), posterior margin of orbit to mid anterior margin of ear; length of forelimb (L1) and hindlimb (L2), from insertion to tip of longest digit (claw included), with limb stretched straight perpendicular to body; and (AG) axilla to inguinal region with body straightened.

The following scale counts and characters were recorded: subdigital scales from tip of digit (4th finger, 4th toe) to basal junction of 3rd and 4th digits (series includes enlarged distal pair); supralabial and infralabial scale rows (beginning immediately posterior to rostral and mental scales, and terminating where there is a noticeable reduction in size, or the labial scales begin to pull away from the lip line [approximately level with mid orbit]); number of small scales contacting the posterior edges of the rostral and mental scales; the number of scales in a longitudinal series along the length of the original tail (along vertebral line); the number of scales across the original tail (transverse count taken across the large scale row closest to widest point of tail); the size of the back, nape and head scales (relative to flank and lateral neck scalation); the presence or absence of a small medially projecting process on the posterior edge of the mental; and finally, the size of the 1st supralabial in relation to the rest of the supralabial row. Specimens included in the morphometric assessment are listed within the species accounts. Additional material examined is listed in S1 Appendix.

Nomenclatural acts

The electronic edition of this article conforms to the requirements of the amended International Code of Zoological Nomenclature, and hence the new names contained herein are available under that Code from the electronic edition of this article. This published work and the nomenclatural acts it contains have been registered in ZooBank, the online registration system for the ICZN. The ZooBank LSIDs (Life Science Identifiers) can be resolved and the associated information viewed through any standard web browser by appending the LSID to the prefix "http://zoobank.org/". The LSID for this publication is: urn:lsid:zoobank.org:pub: C410144B-EC99-4AA4-8780-A2858356CF32. The electronic edition of this work was published in a journal with an ISSN, and has been archived and is available from the following digital repositories: PubMed Central, LOCKSS.

Results

Phylogenetic relationships

Monophyly of *D. conspicillatus sensu lato* was strongly supported in all analyses (Fig. 2A–B). Within this clade we identified the nine major lineages corresponding to the candidate species identified by Oliver et al. [30], specifically; *D. conspicillatus sensu stricto*—widespread in the arid zone and extending into the AMT; lineage A—Gulf region, north Queensland; lineage B—western Pilbara and Carnarvon region, Western Australia; lineage C—widespread arid zone; lineage D—western Top End, Northern Territory; lineage E—Kimberley, Western Australia; lineage F—Channel Country, western and central Queensland and far north-west New South Wales; lineage G—around Townsville, Queensland; and lineage H—gulf country, north Queensland (Fig. 1). Monophyly of all major clades is strongly supported, and mean uncorrected genetic divergence between lineages is relatively high (11.3–22.5%) (S1A Table). Lineages B, C, D and E form a clade that is well supported as sister to another clade comprising *D. conspicillatus sensu stricto* and lineage A. Collectively these clades (*D. conspicillatus sensu stricto* and

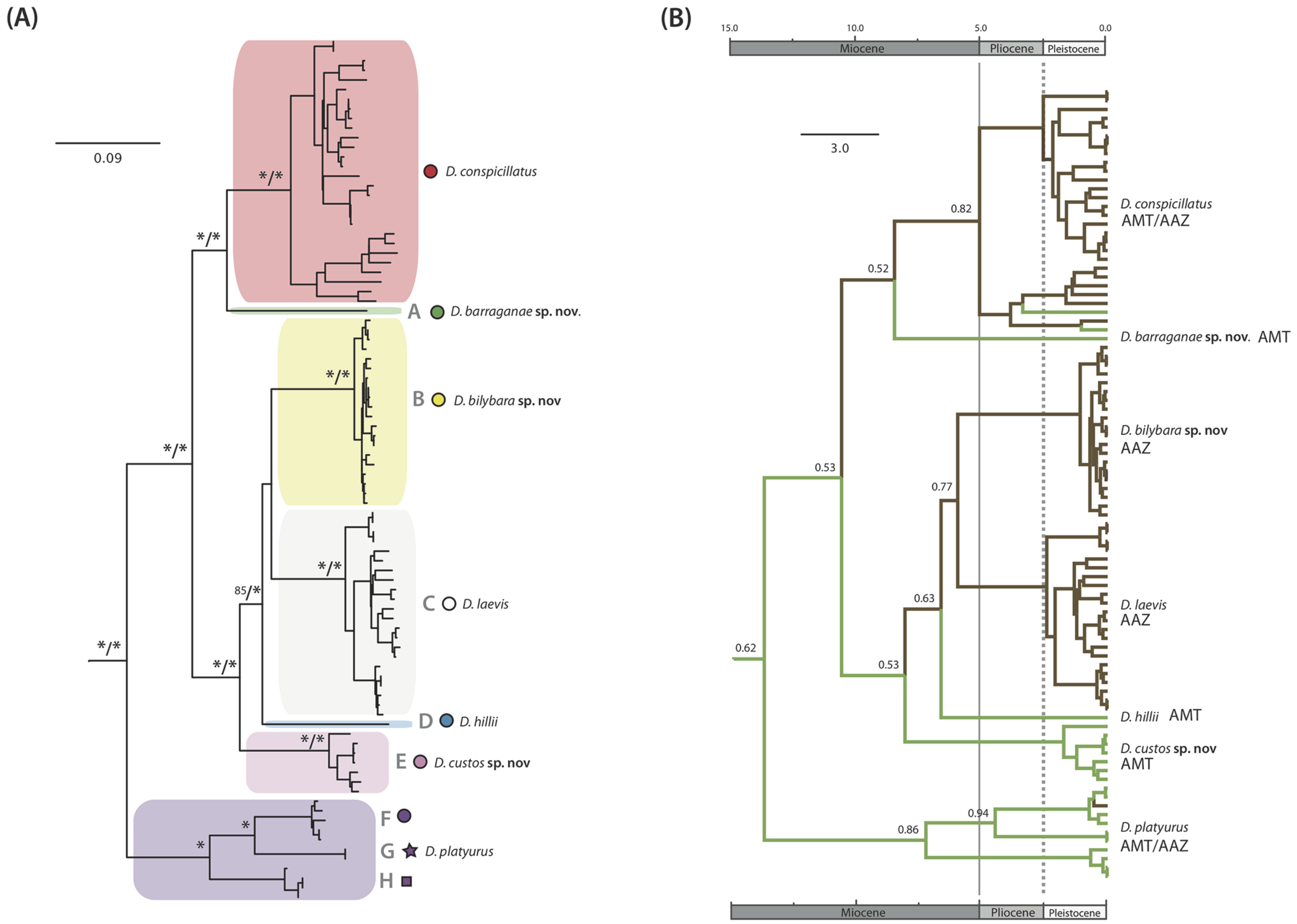

Fig 2. Phylogenetic summaries. (A) Maximum likelihood phylogeny based on the mtDNA gene *nd2* estimated using RaxML for all major lineages within the *D. conspicillatus* complex. Lineage names A-H follow Oliver et al. 2009. Clades are colour-coded to match distributions in Figs. 1 and 3. Nodes with ML support above 95 and Bayesian support (BEAST) above 99 (respectively) are indicated with an asterisk (*). (B) Chronogram and ancestral biome states for the seven species in the revised *D. conspicillatus* group estimated using BEAST and calibrated with a 3% pairwise mean rate of molecular evolution. Green lineages are from outside the central Australian arid zone (defined by a moisture index of less than 0.4), brown lineages are from inside the Australian arid zone, and the probability (i.e. percentage of reconstructions that feature the observed state) of the inferred ancestral habitat is indicated for major nodes.

doi:10.1371/journal.pone.0111895.g002

A–E) are well supported as sister to the most divergent clade of the complex which contains lineages F–H from eastern Australia.

Divergence dates and biome evolution

Topology and support values for the phylogeny of the *D. conspicillatus* complex co-estimated by BEAST were congruent to those from RAxML (Fig. 2A–B). Age estimates derived from application of a mean pairwise sequence divergence rate of 3% per million years suggests the deepest divergences within the complex (including the majority of the lineages and major clades discussed above) occurred in the late Miocene (~ 5–10mya). Where dense sampling was avaliable the accumulation of diversity within most candidate species is estimated to have occurred during the Pleistocene, with the exception of the clade comprising lineages F–H which includes a number of deep and relatively poorly sampled lineages distributed across Queensland.

Distributional data based on morphotyped samples indicates that of the nine major lineages in the *D. conspicillatus* complex, five (A, D, E, G and H) are absent from the central arid zone (and are mostly restricted to the AMT), two (*D. conspicillatus sensu stricto* and F) occur in the both AMT and AAZ, and two (lineages B and C) are restricted to the AAZ (although the range of the former is centred on the comparatively mesic Pilbara [see discussion below]) (Fig. 3). Support for most ancestral state reconstructions was relatively weak, but our results suggest that monsoonal environments are ancestral and also provide strong evidence that there have been multiple transitions between the arid and monsoonal areas (Fig. 2B).

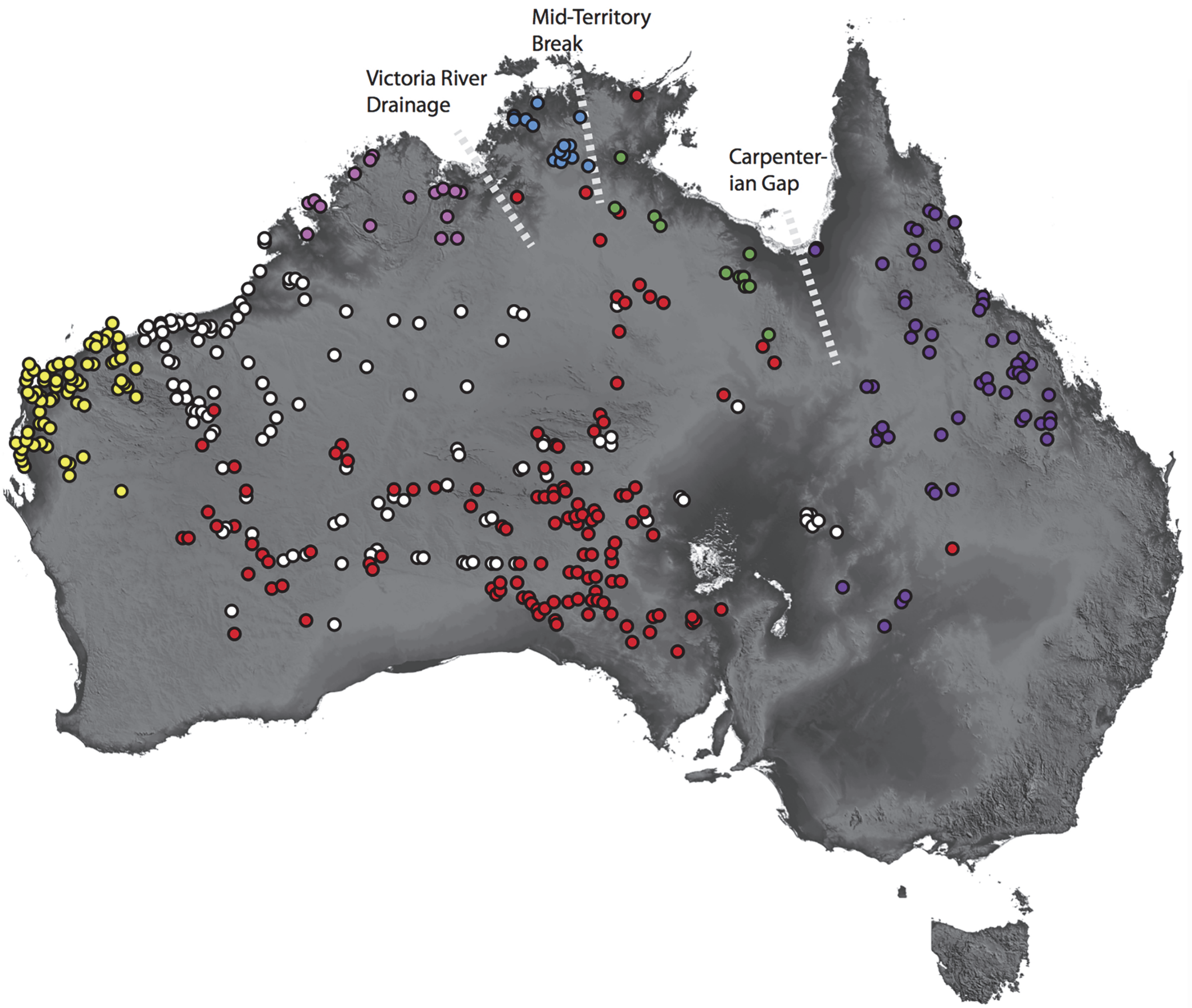

Fig 3. Distribution of species in the *Diplodactylus conspicillatus* complex based on morphological analyses of holdings in Australian museums. Pink = *D. custos* **sp. nov**., Blue = *D. hillii*, Green = *D. barraganae* **sp. nov**., Purple = *D. platyurus*, Red = *D. conspicillatus*, White = *D. laevis*, Yellow = *D. bilybara* **sp. nov**. Dashed lines indicate approximate locations of biogeographic breaks mentioned in the text.

doi:10.1371/journal.pone.0111895.g003

Population genetics

Genetic sampling for a number of lineages was sparse, and only *D. conspicillatus sensu stricto* (n = 28), lineage B (n = 60) and lineage C (n = 62) were well sampled with good geographic spread across their distributions (S1B Table). *Diplodactylus conspicillatus sensu stricto* included two divergent sublineages (>10% divergence) with distributions in arid and monsoonal areas of Queensland and the Northern Territory, and in arid South Australia and Western Australia, respectively. These two sublineages also showed evidence of further structure (especially the north sublineage which showed mean genetic divergence of 6.7%). Lineage B showed low structure across its distribution in the western/southern Pilbara and Carnarvon Basin (mean 1.3%), while lineage C was characterised by moderate mitochondrial haplotypic diversity (mean 3.9%), especially in the western edge of its range in the northeastern Pilbara and surrounding regions.

Our analyses of population size change using summary statistics indicate that lineages B and C from the AAZ both had significant and large negative values for Fu's *Fs* (−33.0 and −14.3, respectively)—consistent with a signature of contiguous range expansion [42]. Tajima's *D* measurements were significantly negative only for lineage B. The other widespread arid zone lineage *D. conspicillatus sensu stricto* had small negative values (-2.55), however these measurements were again not significant. There was no evidence of deviation from neutral expectations in the largely AMT lineages E or F–H (considered together), which can be interpreted demographically as stable populations in mutation-drift equilibrium [42] (S1C Table). As sampling for all of these northern lineages was very sparse these results should be interpreted with caution.

Morphology

Morphological analyses of genotyped specimens revealed a suite of characters that diagnosed most of the major lineages identified (see Table 2): the presence or absence of a) a well developed canthal stripe (Fig. 4A–B), b) an enlarged lst supralabial (Fig. 4C–D), c) enlarged plate-like scales on the mid-dorsum and nape (Fig. 5 A–D), d) clearly defined transverse rows of enlarged scales across the original tail, and e) original tail with a pointed, attenuated tip (Fig. 6A–F). Using these characters we were readily able to assign specimens to most of the lineages identified by the molecular analyses, and determine the identity of museum specimens for which molecular data were unavaliable. The exception to this general pattern was the three eastern lineages (F, G, H), which could be readily diagnosed from all other lineages in lacking a distinctively enlarged first supralabial scale and in having no (or a poorly developed) canthal stripe, but aside from evidence of size differentiation did not show such consistent diagnostic morphological features from each other.

Discussion

Species diversity

Delimiting species boundaries involves integration of independent data sources to identify distinct evolutionary lineages [46], ideally including information from mulitple nDNA loci, morphology, geography, ecology and reproduction [47]. The limitations of using mtDNA alone to infer species boundaries and historical phylogeography are widely recognised [48]. However, six of the nine major mitochondrial lineages we identified (*D. conspicillatus sensu stricto* and lineages A–E) are deeply divergent from each other and in addition can be readily diagnosed by a suite of morphological characters (distinctive features of scalation on the dorsum and original tails). Thus two lines of evidence support the hypothesis that these represent evolutionarily

Table 2. Summary of key meristic and mensural data for species in the *D.conspicillatus* complex.

Character	*D. conspicillatus*	*D. hillii*	*D. laevis*	*D. platyurus*	*D. barraganae sp. nov.*	*D. bilybara sp. nov.*	*D. custos sp. nov.*
**SVL**	54.4 ± 4.0	46.3 ± 5.5	54.9 ± 4.7	48.7 ± 5.2	41.8 ± 6.8	48.8 ± 5.2	50.6 ± 5.8
	(47.1–62.7, n = 32)	(25.1–52.3, n = 19)	(42.4–64.9, n = 30)	(40.6–60.2, n = 36)	(25.1–49.5, n = 17)	(39.2–60.8, n = 28)	(42.2–58.2, n = 13)
**AG (% SVL)**	50.4 ± 0.0	48.5 ± 0.0	51.8 ± 0.0	48.8 ± 0.0	49.9 ± 0.0	49.6 ± 0.0	49.3 ± 0.0
	(45.7–57.4, n = 32)	(43.9–56.2, n = 19)	(46.0–57.3, n = 30)	(42.4–56.4, n = 32)	(45.1–55.8, n = 17)	(44.6–54.2, n = 27)	(45.7–52.8, n = 10)
**Forelimb (% SVL)**	30.7 ± 0.0	31.7 ± 0.0	29.5 ± 0.0	32.43 ± 0.0	32.8 ± 0.0	31.1 ± 0.0	31.1 ± 0.0
	(27.7–34.0, n = 32)	(29.2–36.0, n = 19)	(25.1–33.9, n = 30)	(28.1–37.3, n = 34)	(29.8–35.0, n = 17)	(24.9–35.9, n = 28)	(26.8–35.6, n = 13)
**Hindlimb (% SVL)**	33.5 ± 0.0	34.0 ± 0.0	32.4 ± 0.0	35.8 ± 0.0	36.0 ± 0.0	33.7 ± 0.0	33.5 ± 0.0
	(28.7–37.3, n = 32)	30.4–37.9, n = 18)	(27.8–39.0, n = 30)	(30.6–40.8, n = 35)	(33.0–38.8, n = 17)	(27.5–38.8, n = 28)	(28.1–39.8, n = 13)
**Tail (% SVL)**	39.6 ± 0.0	34.4 ± 0.0	45.7 ± 0.0	35.7 ± 0.0	36.7 ± 0.0	43.9 ± 0.0	42.1 ± 0.0
	(32.3–44.3, n = 28)	(30.6–39.3, n = 19)	(36.7–52.3, n = 26)	(28.4–42.6, n = 30)	(30.1–43.3, n = 14)	(33.9–48.3, n = 22)	(34.9–51.3, n = 11)
**HW (% HL)**	80.9 ± 0.1	74.5 ± 0.1	90.9 ± 0.1	81.5 ± 0.0	81.1 ± 0.1	87.1 ± 0.0	81.5 ± 0.1
	(68.5–91.6, n = 32)	64.2–85.2, n = 18)	(79.4–98.7, n = 29)	(73.3–89.4, n = 33)	(73.1–92.9, n = 17)	(79.9–92.1, n = 28)	(73.7–89.1, n = 12)
**HD (% HL)**	50.2 ± 0.0	47.9 ± 0.0	54.0 ± 0.0	47.1 ± 0.1	47.7 ± 0.0	49.8 ± 0.0	47.3 ± 0.0
	(43.6–55.5, n = 32)	(43.8–54.4, n = 19)	(47.8–64.0, n = 30)	(36.0–54.4, n = 33)	(40.1–53.4, n = 17)	(43.7–55.7, n = 28)	(42.5–52.5, n = 12)
**Snout (% HL)**	46.6 ± 0.0	47.0 ± 0.0	46.0 ± 0.0	45.0 ± 0.0	46.5 ± 0.0	45.3 ± 0.0	44.5 ± 0.0
	(42.8–52.0, n = 32)	(44.6–50.5, n = 19)	(42.7–49.7, n = 30)	(42.0–47.6, n = 37)	(42.7–53.4, n = 17	(43.3–47.9, n = 28)	(38.8–48.3, n = 12)
**EE (% HL)**	28.6 ± 0.0	25.9 ± 0.0	29.6 ± 0.0	26.8 ± 0.0	27.0 ± 0.0	28.2 ± 0.0	27.6 ± 0.0
	(25.4–31.5, n = 32)	(21.8–30.4, n = 19)	(24.2–34.6, n = 30)	(23.4–32.8, n = 33)	(24.8–30.4, n = 17)	(24.5–33.5, n = 28)	(24.3–30.4, n = 12)
**Rostral contact**	5.2 ± 0.5	5.0 ± 0.0	5.1 ± 0.3	8.7 ± 1.7	5.1 ± 0.4	5.3 ± 0.5	5.6 ± 0.9
	(5–7, n = 32)	(5, n = 19)	(5–6, n = 30)	(5–13, n = 35)	(5–6, n = 17)	(5–6, n = 28)	(5–8, n = 14)
**Mental contact**	11.2 ± 1.0	10.1 ± 1.1	12.1 ± 1.5	10.6 ± 1.5	11.1 ± 1.1	11.3 ± 1.2	12.3 ± 1.5
	(9–13, n = 32)	(8–12, n = 19)	(10–15, n = 30)	(8–15, n = 36)	(10–14, n = 17)	(9–14, n = 28)	(10–15, n = 16)
**Supralabials**	15.6 ± 2.2	18.4 ± 2.2	15.6 ± 2.2	15.5 ± 1.6	17.4 ± 1.4	16.8 ± 1.9	15.9 ± 1.1
	(12–20, n = 32)	(14–24, n = 18)	(12–20, n = 32)	(11–19, n = 37)	(15–19, n = 17)	(13–20, n = 27)	(14–18, n = 15)
**Infralabials**	14.9 ± 2.3	17.4 ± 2.5	15.7 ± 2.2	15.8 ± 1.7	16.2 ± 1.0	17.0 ± 2.7	16.9 ± 2.7
	(10–19, n = 32)	(13–21, n = 16)	(11–20, n = 30)	(12–20, n = 37)	(13–18, n = 17)	(12–21, n = 27)	(13–21, n = 14)
**lamellae finger 4**	11.1 ± 0.9	11.8 ± 1.0	11.5 ± 1.0	10.9 ± 1.1	10.9 ± 1.4	11.5 ± 1.7	11.0 ± 1.6
	(10–13, n = 32)	(10–14, n = 19)	(10–13, n = 29)	(9–13, n = 35)	(8–14, n = 17	(8–15, n = 28)	(9–16, n = 15)
**Lamellae toe 4**	12.7 ± 1.2	13.1 ± 0.9	13.6 ± 1.1	11.9 ± 1.2	12.2 ± 1.4	13.2 ± 1.5	11.9 ± 1.2
	(11–16, n = 32)	(12–15, n = 19)	(11–16, n = 29)	(9–15, n = 36)	(10–15, n = 17)	(10–17, n = 28	(10–15, n = 15)
**TW/TL**	46.7 ± 0.0	54.6 ± 0.1	44.9 ± 0.1	60.8 ± 0.1	54.6 ± 0.1	46.7 ± 0.0	46.0 ± 0.1
	(39.7–56.4, n = 28)	(40.4–72.5, n = 18)	(34.4–58.5, n = 26)	(44.3–78.8, n = 30)	(46.8–63.8, n = 14)	(41.2–58.1, n = 22)	(35.0–54.7, n = 10)
**Scale rows on tail**	32.7 ± 2.5	25.6 ± 2.3	43.0 ± 2.5	25.1 ± 3.2	34.9 ± 2.4	40.1 ± 4.7	33.4 ± 3.9
	(28–39, n = 28)	(23–30, n = 19)	(37–50, n = 28)	(20–34, n = 33)	(31–39, n = 14)	(31–49, n = 23)	(28–41, n = 12)
**Scales across tail**	12.7 ± 0.9	11.8 ± 1.5	14.2 ± 1.3	13.2 ± 1.5	13.9 ± 1.4	13.2 ± 1.3	12.9 ± 0.9
	(12–15, n = 28)	(10–15, n = 18)	(12–19, n = 28)	(10–18, n = 33	(12–16, n = 14)	(10–15, n = 24)	(12–14, n = 12)
**Canthal stripe bold, clearly defined**	Yes	Yes	Yes	No	Yes	Yes	Yes
**1st supralabial large (contacting nasal)**	Yes	Yes	Yes	No	Yes	Yes	Yes
**Tail shape**	Spade-like	Spade-like	Attenuated tip	spade-like	spade-like	Attenuated tip	Attenuated tip
**Tail scales in rows**	Yes	No	Yes	Yes	Yes	Yes	Yes
**Mid dorsal scales enlarged**	Yes	No	Yes	Yes	No	Yes	Yes
**Scales on nape enlarged**	No	No	Yes	No	No	No	No

doi:10.1371/journal.pone.0111895.t002

distinct and diagnosable lineages (species). Three further mitochondrial lineages identified previously (F–H) [30] form a strongly supported clade that can be readily diagnosed from all other members of this complex by their distinctive labial scalation, but are more difficult to diagnose from each other, and are represented by few samples in our analyses. *Diplodactylus conspicillatus sensu stricto* also includes two moderately divergent sublineages (Fig. 2) that were flagged but not named by Oliver et al. [30]. Genetic divergences between these sublineages are

lower than between the recognised candidate taxa, sampling for one is again sparse, and we did not find diagnostic morphological characters. More detailed sampling and additional nDNA data sources are required to resolve the taxonomic status of these remaining mitochondrial lineages; and for the time being we note their potential significance, but do not recognise any as distinct species.

Formal diagnoses and descriptions of the seven species we recognise within the *D. conspicillatus* complex are provided in the systematics section at the end of this paper, however for the remainder of this discussion we consider each of these seven species as separate entities and use our revised binomial arrangement (see Figs. 1–3 for a summary of phylogenetic and distributional information).

Geographic structuring and bioregions

This study has confirmed that species diversity within the *D. conspicillatus* complex in both the AAZ and AMT is much higher than previously recognised. While significant sampling gaps remain (especially in northern Australia), based on combined genetic datasets and morphological assessments, we were able to infer the broad geographic distributions of most taxa. Of the seven species, three are endemic to the AMT, two are endemic to AAZ and two occur in both biomes. Our systematic analysis of this previously undetected diversity therefore provides oppurtunities to contrast patterns of diversity within biomes, and examine the potential timing and nature of transitions between them.

The distributions of the species endemic to the AMT broadly correspond to seperate regions of endemism; specifically the Kimberley, Top End and Gulf [16,17, 49], and are separated by putative biogeographic barriers [49,50] (Fig. 3). *Diplodactylus custos* **sp. nov**. is endemic to the Kimberley region. The moderate genetic diversity of this species contrasts with very high genetic diversity of some saxicoline gecko lineages endemic to the same region [6,16,23] but is similar to Kimberley endemic toadlet lineages more strongly associated with savanna woodlands [49]. Based on current sampling, the eastern extent of the range of *D. custos* **sp. nov**. appears to broadly correspond with the putative Victoria River drainage barrier [50,51]. Genetic sampling for the remaining two AMT endemics was threadbare. However, based on diagnostic morphological data, *D. hillii* is only known from the western Top End (east of the Arnhemland Escarpment). A disjunction in this region (the Mid-Territory Break) has been detected in *Uperolia* toadlets [49] and may be related to variations in geology and topography around the Arnhem Escarpment. The distribution of *D. barraganae* **sp. nov**. along the Gulf of Carpentaria also mirrors that of a number of other lizard and frog clades [16,49]. Another putative biogeographic barrier, the Carpenteria Gap [17,49,52], separates *D. barraganae* **sp. nov**. from the easternmost species *D. platyurus*. In this region the clay plains in the hinterland of the Gulf of Carpentaria may form an important divide between the open woodlands of the Top End and Cape York Peninsula [17].

Diplodactylus platyurus has a distribution centred on Queensland, ranging from subhumid areas in the east and north and extending into the periphery of the AAZ in the west. This species contains two deeply divergent lineages from the AMT (Fig. 2, lineages G and H), while samples from a wide region along the eastern periphery of AAZ (lineage F) cluster together in a third lineage. The distribution of lineage F corresponds with the periodically flooded Channel country in western Queensland, a region that provides a set of microhabitats that are not typical of the AAZ, and is home to a suite of taxa that are absent from less watered areas to the west [16,53,54].

Diplodactylus conspicillatus also occurs in the AMT and AAZ, although the vast majority of its range is in the latter biome. This species includes divergent sublineages distributed to the

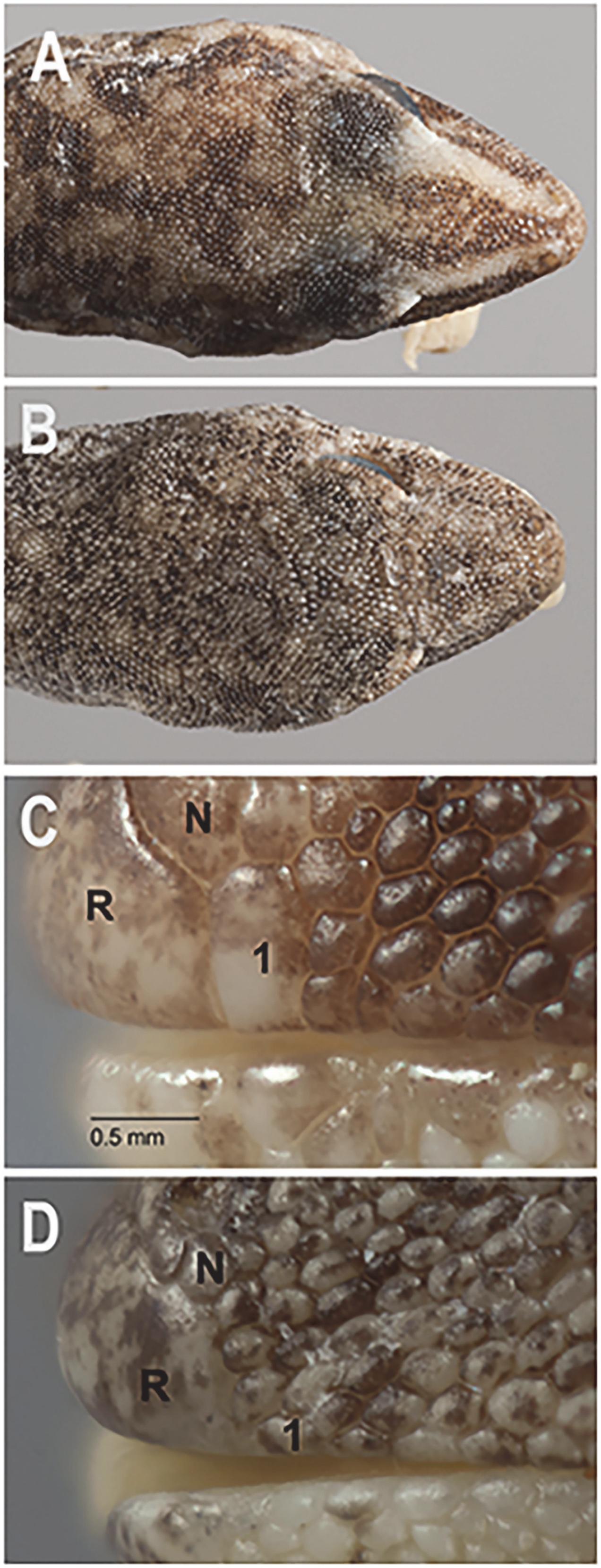
A
B
C
N
R
1
0.5 mm
D
N
R
1

Fig 4. Variation in snout colouration and scalation. (A) Canthal stripe present (*D. conspicillatus* SAMA R32133); (B) Canthal stripe absent or very weakly developed (*D. platyurus* AMS R158426). Condition A applies to all members of the *D. conpicillatus* group except *D. platyurus*. (C) 1st supralabial (1) greatly enlarged and contacting nasal scale (N) (*D. conspicillatus* SAMA R42589); (D) 1st supralabial (1) not enlarged and widely separated from ventral edge of nasal scale (N) (*D. platyurus* AMS R 158426). Condition C is found in all members of the *D. conspicillatus* group except *D. platyurus*. Note in images C & D that R = rostral scale (Images: A & B Jeff Wright, QM; C & D Geoff Thompson, QM).

doi:10.1371/journal.pone.0111895.g004

north and west of the Lake Eyre Basin, respectively, and does not show a strong signal of range expansion (although sampling for the north lineage was sparse). These data suggest that this clade has persisted and diversified within or close to the edge of the AAZ since the late Miocene. The potential roles of the vast lower Lake Eyre Basin and highly mobile sand dunes of the Simpson Desert in shaping phylogeographic patterns within this region warrant further investigation [55,56]. The distribution of these two major sublineages also broadly corresponds with the transition from the slightly higher and more reliable summer rainfall deserts in the north, to the drier and more winter rainfall deserts to the south [57,58]; these gradients of seasonality and precipitation may provide climatic axes over which taxa could diversify within the AAZ.

Only two species (*Diplodactylus bilybara* **sp. nov**. and *D. laevis*) have distributions entirely confined to the AAZ, and both show a strong signal of population expansion and relatively shallow intraspecific genetic diversity over most of their ranges. Low genetic diversity has been detected in a number of widespread arid zone taxa, and is thought to reflect relatively recent and major demographic shifts through severe glacial cycles of the Plio-Pleistocene [7,9,14,18,59]. The distribution of the two AAZ endemic taxa in this gecko complex is also outwardly contrasting; *Diplodactylus bilybara* **sp. nov**. is restricted to the southern and coastal west Pilbara and Carnarvon regions, while *Diplodactylus laevis* has a vast distribution across central Australia. However, mitochondrial diversity within the latter species is concentrated along the the westernmost portion of its range, close to the range of the former [24]. This distribution of phylogenetic diversity supports previous work suggesting that the Pilbara and nearby areas have been an important zone of persistence and diversification at the western periphery of the arid zone [6,7,15].

Contrasting diversification in the AMT and AAZ

The overall timeframe and pattern of divergences in the *D. conspicillatus* complex implies that intensifying aridity since the late Miocene has played a central but at times contrasting role in shaping diversification in the AMT and AAZ [9]. Lineages in older and shrinking mesic zones (such as the AMT) are restricted to relatively small and largely allopatric patches of habitat, a distribution indicative of long-term persistence, but with increasing attenuation and potentially non-adaptive diversfication [6,8,9]. In contrast, the vast sandy plains of the central AAZ appear to have a more dynamic recent history of ecological adaptation, colonisations and large scale range shifts, but less in the way of intra-regional diversification and speciation [9,14].

Bayesian reconstruction of biome shifts within the *D. conspicillatus* complex suggests that mesic biomes are ancestral. While support for many ancestral state reconstructions in the tree is low, this overall pattern is consistent with the widely held idea that the Australian arid biota is largely derived from peripheral and more mesic biomes [9,14,60]. Furthermore, as intimated above, our simple binary classification of arid vs not arid is also probably overly simplistic; while peripheral regions such as the Pilbara (*D. bilybara* **sp. nov**.) and much of the Channel country (*D. platyurus* lineage F) are technically within the AAZ, compelling arguments can be made as to why they could be viewed as mesic refugia [24,53,61]. Under this interpretation only two widespread species (*D. conspicillatus* and *D. laevis*) would be considered successful

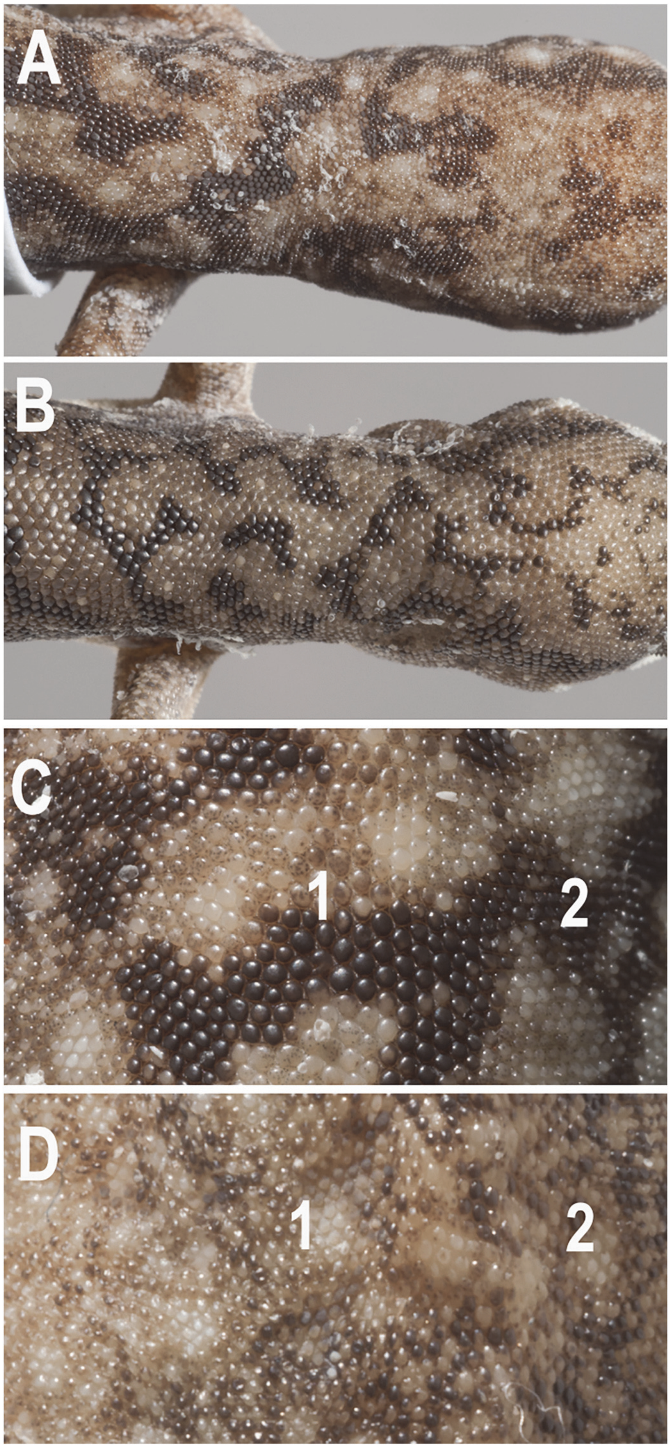

Fig 5. Variation in dorsal scalation. Arrangement of scales on neck and back of head—(A) scales small, only slightly larger those on side of neck (*D*. *conspicillatus* SAMA R42569); B) *D*. *laevis* (SAMA R56481) scales on nape and back of head large and plate-like and continuous with enlarged dorsal scales on trunk. Condition A applies to all members of the *D*. *conspicillatus* group except *D*. *laevis*. (C) Plate-like vertebral scales (1), appreciably larger than those of dorsolateral area (2) (*D*. *conspicillatus* SAMA R42569); (D) Dorsal scales granular, those of vertebral area (1) not appreciably larger than those of the dorsolateral area (2) (*D*. *hillii* NTM R27363). Condition D only occurs in *D*. *hillii* and *D*. *barraganae* **sp. nov**. (Images: A–C, Jeff Wright QM; D, Peter Waddington QM).

doi:10.1371/journal.pone.0111895.g005

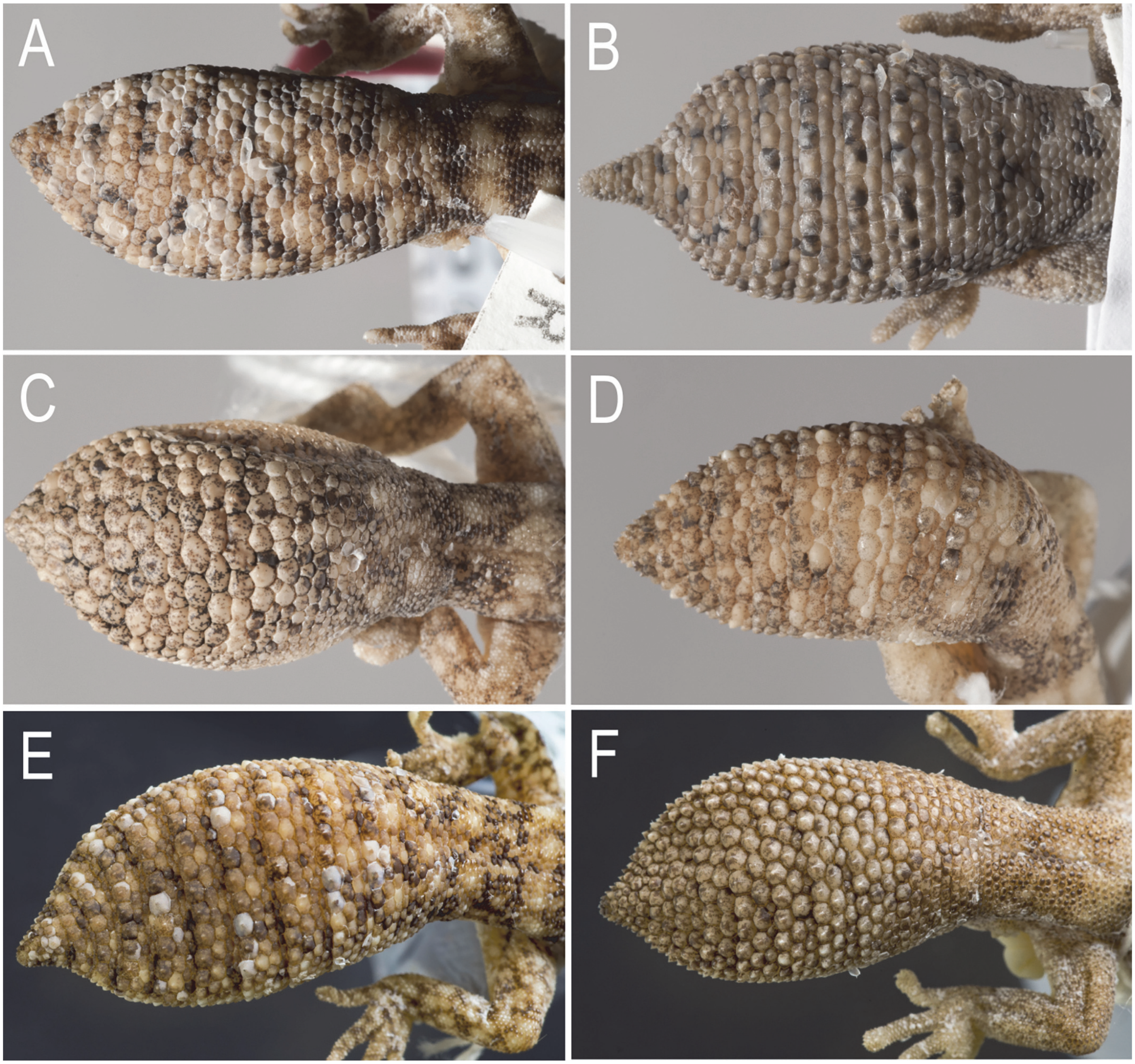

Fig 6. Scalation and shape of original tails. (A) *D. conspicillatus* (SAMA R32133) tail spade-like and lacking attenuated tip; dorsal scale arrangement transverse, includes rows of both large and small scales. (B) *D. laevis* (SAMA R56481) tail ends in distinct attenuated tip; dorsal scale arrangement transverse, includes rows of both large and small scales (photographs—Jeff Wright, QM). (C) *D. hillii* (NTM R24364) tip spade-like; dorsal scales relatively large and not arranged in clear transverse rows. (D) *D. barraganae* (NTM R21395) tip spade-like; dorsal scales arranged in transverse rows which may include rows of both large and small scales. (E) *Diplodactylus bilybara* (SAMR22819) short to moderate, acute attenuated extension at tip; alternating transverse rows of large and smaller dorsal scales (F) *D. custos* (WAMR164780) tip moderately attenuated; dorsal scales arranged in transverse rows of relatively uniform size (Images: Jeff Wright, QM).

doi:10.1371/journal.pone.0111895.g006

colonists of the AAZ, and the diversity in this zone would be rendered more clearly depaurate, recent and derived than that of the AMT.

Our phylogeny also strongly indicates there have been repeated, independent transitions between the AMT and the AAZ; detectable at both interspecific (*D. barraganae* **sp. nov**. and *D.*

conspicillatus) and intraspecific levels (*D. conspicillatus* and *D. platyurus*). In an analysis of the plant biota of the Southern Hemisphere (including Australia), Crisp et al. [60] found that transitions between biomes were relatively rare in general, and transitions into arid biomes from monsoonal (savanna) environments were particularly rare. However, at least in Australia, the AMT remains the least studied of the major biomes [17,62] and this pattern may to some extent have reflected a lack of data. Even in this study, our sampling from the AMT is also sparse, and additional material will likely refine understanding. However, this and other broadscale analyses increasingly suggest that the history and evolution of many lineages in the the AMT and AAZ has been intimately linked since at least the late Miocene [14,16].

A final notable pattern is that the two most widespread arid zone taxa (*D. conspicillatus* and *D. laevis*) have broadly overlapping distributions in the southern and eastern arid zone (Figs. 1 & 3); the only instance of widespread sympatry within the *D. conspicillatus* complex. Relatively closely related congeners with overlapping distributions in the AAZ have been found in other widely distributed Australian lizard radiations [12,21,63]—sympatric diversity in these closely related lizard taxa may be further evidence of a relatively dynamic recent history of range expansion and ecological diversification in the vast but young arid biome [9].

Hyper-diverse species complexes and evolutionary biology

Nearly 100 new and widely accepted Australian squamate species have been described since 2000; indeed 2007 was the most 'productive' year on record for Australian reptile taxonomy (30 well-characterised species) [64]. While some of this new biodiversity represents singletons or other novelties uncovered by fieldwork, many 'new' species have been detected within morphologically cohesive nominal 'species' that actually comprise a larger number of unrecognised taxa (five or more). In the Australian context such complexes are particularly well documented in geckos [7,8,14,16,25,61,65,66], but have also been detected in blindsnakes [67], skinks [68–70], Donnellan pers com], and dragons [27]. While in some cases these complexes appear to comprise genuinely morphologically cryptic taxa, in others (such as the *D. conspicillatus* group) careful work oftens reveals a suite of diagnositic morphological characters.

There is little sign that the rate of discovery is slowing down, and if anything, it may increase in the short-term as sampling across northern Australia becomes more comprehensive, and researchers assemble increasingly large genomic datasets and develop new analytical methods [23,71]. Even our assessment of the *D. conspicillatus* group is likely to be an underestimate; there is further deep genetic diversity in *D. conspicillatus* and *D. platyurus*, many areas remain poorly sampled, and there are two morphologically distinct specimens from Cape York in north-Queensland for which no genetic data is available (see below). Thus, as with so many Australian lizard groups, further analyses will likely show that species diversity still remains underestimated.

Many arguments for the importance of continued efforts to properly understand this biodiversity for conservation and management purposes have been outlined in a compelling fashion elsewhere [22,72]. However we would like to conclude by further re-emphasising that systematic work on these complexes also has a tendency to reveal interesting macro-evolutionary patterns. For example, using the complexes of Australian lizards listed earlier in this section as examples; systematic work has revealed parthenogenesis [56], ancient vicarience and long-term persistence [8,61], rapid radiation [70], morphological conservatism or parallelism [27,67,68] and provided insight into the comparative history of biomes or regions of endemism ([14,24], this study). Further work to resolve other species complexes will continue to provide a framework for broader insights into macroevolutionary processes.

Systematics

Nomenclatural Synopsis

Diplodactylus conspicillatus was described from specimens collected by Mr P. M. Byrne at Charlotte Waters in the southern Northern Territory (NT). In subsequent taxonomic work three additional, closely allied taxa have been named: *Diplodactylus hillii* Longman [73] from Port Darwin, N.T.; *Gymnodactylus laevis* Sternfeld [74] from Hermannsburg Mission, N.T. and *Diplodactylus platyurus* Parker [75] from Torrens Creek, Queensland (QLD).

Diplodactylus hillii (as *D. hilli*) was placed in the synonymy of *D. conspicillatus* by Kluge [76]. In this work Kluge only examined type material held in Australian museums and no consideration was given to the taxonomic status of *G. laevis* or *D. platyurus*. However, when Kluge revisited *D. conspicillatus* for his revision of the genus [77], both *G. laevis* and *D. platyurus* were also listed with *D. hillii* in the synonymy of *D. conspicillatus* (although the *G. laevis* type material is not listed amongst the specimens examined). Two of these synonyms (*D. hillii* and *D. platyurus*) were resurrected from the synonymy of *D. conspicillatus* by Wells and Wellington [78] but, as no justification was given, this action was widely ignored. Kluge's *D. conspicillatus* synonymy was followed by Cogger [29] who examined the type specimens of all the listed synonyms.

Based on a combination of morphology and genetics the available names can readily be assigned to the various taxa under consideration here. Key diagnostic characters are discussed in detail in the species accounts.

Species group diagnosis

Our concept of the *D. conspicillatus* group includes only species that are part of a strongly supported clade of related forms that have previously been synonymised or confounded with the nominate species. This is contra Kluge [77] and Storr et al. [79], who included the nominate species and some or all of *D. kenneallyi*, *D. pulcher* and *D. savagei*; the phylogenetic relationships of which, based on available data, remain unclear. However, they do not show any evidence of a strong or close affinity to the *D. conspicillatus* group [25].

All species in the *Diplodactylus conspicillatus* group can be distinguished from their congeners by the following combination of characters: all or most supralabials small and granular, at most only one enlarged anterior (1st) supralabial; terminal lamellae on fingers at most only slightly wider than digit; other prominent enlarged subdigital lamallae absent; tail short, as wide or wider than body, depressed with heterogenous scalation, usually bearing large plate-like scales and/or conical tubercules arranged in transverse rows; and dorsal colouration extremely variable, but never consisting of large clearly defined bands or blotches. Comparisons in the following species accounts are restricted to taxa in the *D. conspicillatus* species group only.

The order of authorships for the three new species herein do not follow that of the paper as a whole.

Diplodactylus conspicillatus **Lucas & Frost 1897**

Variable Fat-tailed gecko

Figs. 4A, 4C, 5A, 5C, 6A, 7A & B, 8

Material Examined. NMV D7535, Charlotte Waters (25° 55' S, 134° 55' E) NT, **lectotype**; NTM R24076–77, Arafura Swamp, Arnhem Land (12° 32' 42" S, 134° 54' 24" E) NT; SAMA R38819, 1km E of Three Ways (19° 26' S, 134° 13' E) NT; NTM R9525, Frenena (19° 26' S, 135° 24' E) NT; QM J92288, S of Mt Isa (21° 08' 43" S, 139° 15' 41" E) QLD; NTM R22130, Toko

Ra., Tobermorey Stn (22° 44' S, 137° 56' E) NT; WAM R110767, Jimblebar East (23° 23' 46" S, 120° 18' 36" E) WA; WAM R110770, Jimblebar East (23° 26' 26" S, 120° 20' E) WA; WAM R166300, 16.8km ENE Blackstone (25° 56' 07" S, 128° 26' 16" E) WA; SAMA R50114, 2.3km WSW of Sentinel Hill (26° 05' 17" S, 132° 25' 41" E) SA; SAMA R52781, 11.7km SSW of Mount Sarah homestead (27° 01' 32" S, 135° 13' 16" E) SA; SAMA R44870, 6km WSW of Womikata Bore homeland (26° 06' 33" S, 132° 05' 57" E) SA; SAMA R46981, 5.6km SSE Mosquito Camp Dam, New Crown Stn (26° 09' 28" S, 134° 30' 49" E) SA; SAMA R51587, 36.9km ESE of Amata (26° 16' 58" S, 131° 29' 12" E) SA; SAMA R44864, 8km NE of Mt Woodroffe (26° 17' 10" S, 131° 48' 20" E) SA; SAMA R38849, 6km W Namatjira/Larapinta Drive junction, Namatjira drive (26° 46' S, 133° 27' E) NT; SAMA R51514, 3.3km SW of Indulkana (26° 59' 18" "S, 133° 16' 58" E) SA; SAMA R41859, 5km S of Blue Hills Bore (27° 10' 39" S, 132° 52' 06" E) SA; AMS R125042, Cunnamulla (28° 04' S, 145° 41' E) QLD; SAMA R42569, SAMA R42574, 169km NE of Emu, GI 51 (28° 14' S, 133° 20' E) SA; SAMA R42589, 20km W (road) of base camp 2 (28° 15' S, 133° 08' E) SA; WAM R85850, 39km E Laverton (28° 28' S, 122° 50' E) WA; AMS R58090, 35.9km WSW of Ely Hill (28° 28' 51" S, 134° 03' 41" E) SA; SAMA 26581, 25km SSW Mabel Ck homestead (29° 10' S, 134° 15' 30" E) SA; SAMA 26227, 30km SW Mabel Ck homestead (29° 10' 30" S", 134° 08' E) SA; SAMA R59454, 62.6km NW Maralinga (29° 46' 14" S, 131° 06' 29" E) SA; SAMA R61024, 218.2km WNW Tarcoola (29° 50' 42" S, 132° 31' 59" E) SA; SAMR59401, 12.6km WNW Maralinga (30° 05' 52" S, 131° 28' 06" E) SA; SAMA R32133, 10km SSW Maralinga (30° 15' 01" S, 131° 32' 45" E) SA; SAMA R20884, Olympic Dam area Roxby Downs (30° 23' S, 136° 53' E) SA; SAMA R45246, Salt Ck crossing, E of Lake Gairdner (31° 33' 20" S, 136° 21' 45" E) SA.

Diagnosis. A large member of the *D. conspicillatus* group (max SVL 62 mm) with a bold canthal stripe and a greatly enlarged first supralabial (contacting ventral edge of nasal scale. Mid-dorsal scales on trunk plate-like and markedly larger than smaller dorsolateral scales. Scales on nape granular and only slightly larger than granules on side of neck. Original tail spade-like and lacking an acute attenuated extension at tip. Scales on dorsal surface of tail arranged in transverse rows (which usually include rows of both large and small scales). Pattern generally spotted and often with numerous dark blotches that contrast strongly with base colour (Fig. 7A–B).

Description. SVL mm 47.05–62.71 (n = 32, mean = 54.40, SD = 4.04). Proportions as % SVL: AG 45.72–57.43 (n = 32, mean = 50.36, SD = 0.03); T 32.30–44.27 (n = 28, mean = 39.63, SD = 0.03); HL 16.38–19.90 (n = 31, mean = 18.53, SD = 0.01). **Head:** moderate and not strongly differentiated from neck; snout longer than diameter of eye. HW 68.5–91.6% HL (n = 32, mean = 80.93, SD = 0.05); HD 43.6–55.5% HL (n = 32, mean = 50.2, SD = 0.04); S 42.8–52. 0% HL (n = 32, mean = 46.63, SD = 0.02); EE 25.4–31.5% HL (n = 32, mean = 28.6, SD = 0.02). Covered in small granular scales; rostral shield large and lacking a medial groove, hexagonal with 5–7 scales in contact with its posterior margin (n = 32, mean = 5.19, mode = 5, SD = 0.48); mental shield hemispherical but sometimes with a slight process extending medially from its posterior margin, 9–13 scales contacting posterior edge (n = 32, mean = 11.16, mode = 12, SD = 1.04); supralabials 12–20 (n = 32, mean = 15.63, mode = 15, SD = 2.16) with the first enlarged and contacting ventral edge of nasal scale (Fig. 4C), the remaining series are small and not differentiated from the adjacent loreal scales; infralabials 10–19 (n = 32, mean = 14.91, mode = 15, SD = 2.32), all small and undifferentiated from adjacent chin scales; eye large, pupil vertical with crenulated margin; ear small and usually horizontally elliptic. **Neck:** broad with small granular scales on dorsal surface that are only slightly larger than the adjacent scales on the lateral surfaces (Fig. 5A). **Trunk:** moderate and somewhat stout; scales generally granular but a broad zone of larger, plate-like scales is present along the back and these contrast in size with the smaller granules on the flanks (Fig. 5C); granules small on ventral surface but increase

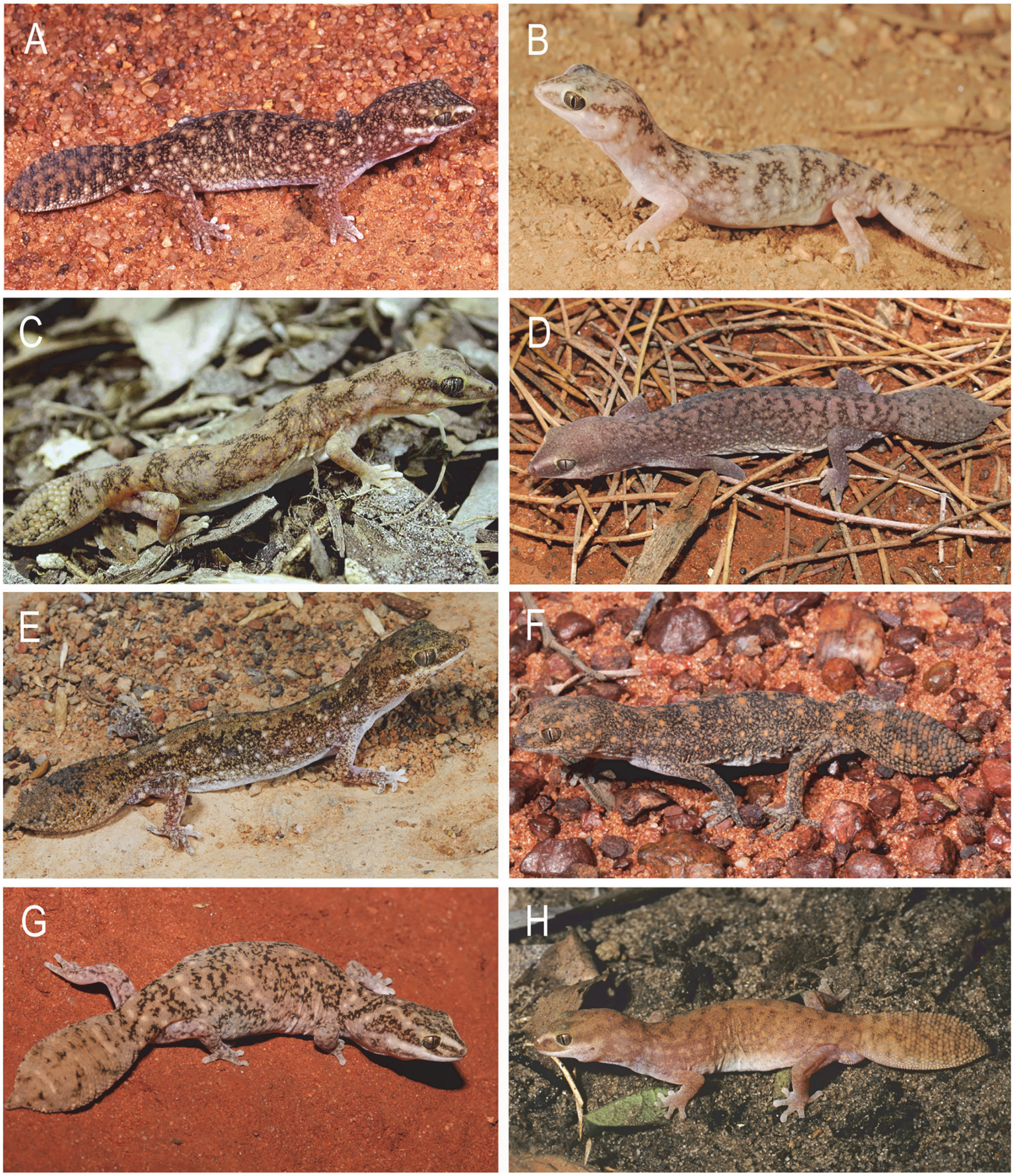

Fig 7. Species of the *D. conspicillatus* complex in life. (A) *Diplodactylus conspicillatus* from 10km north of Barkley Hwy on Ranken to Alexander Station Road, north-eastern Northern Territory (Image: Ross Sadlier); (B) *Diplodactylus conspicillatus* Alice Springs, Northern Territory (Image: Eric Vanderduys); (C) *D. hillii*, Dorat Road, Northern Territory (Image: Paul Horner); (D) *Diplodactylus laevis* in life from Morgan Range, Western Australia (Image: Mark Hutchinson); (E) *Diplodactylus platyurus*, Brooklyn Station, north Queensland (Image: Eric Vanderduys); (F) *Diplodactylus platyurus* Myendetta Stn, Charleville, Queensland (Image: Steve Wilson); (G) *Diplodactylus bilybara* **sp. nov**. Onslow, Western Australia (Image: Ryan Ellis); (H) *Diplodactylus custos* **sp. nov**. Gibb River Road turnoff via Wyndham, Western Australia (Image: Steve Wilson). There are currently no images available of *D. barraganae* **sp. nov**. in life.

doi:10.1371/journal.pone.0111895.g007

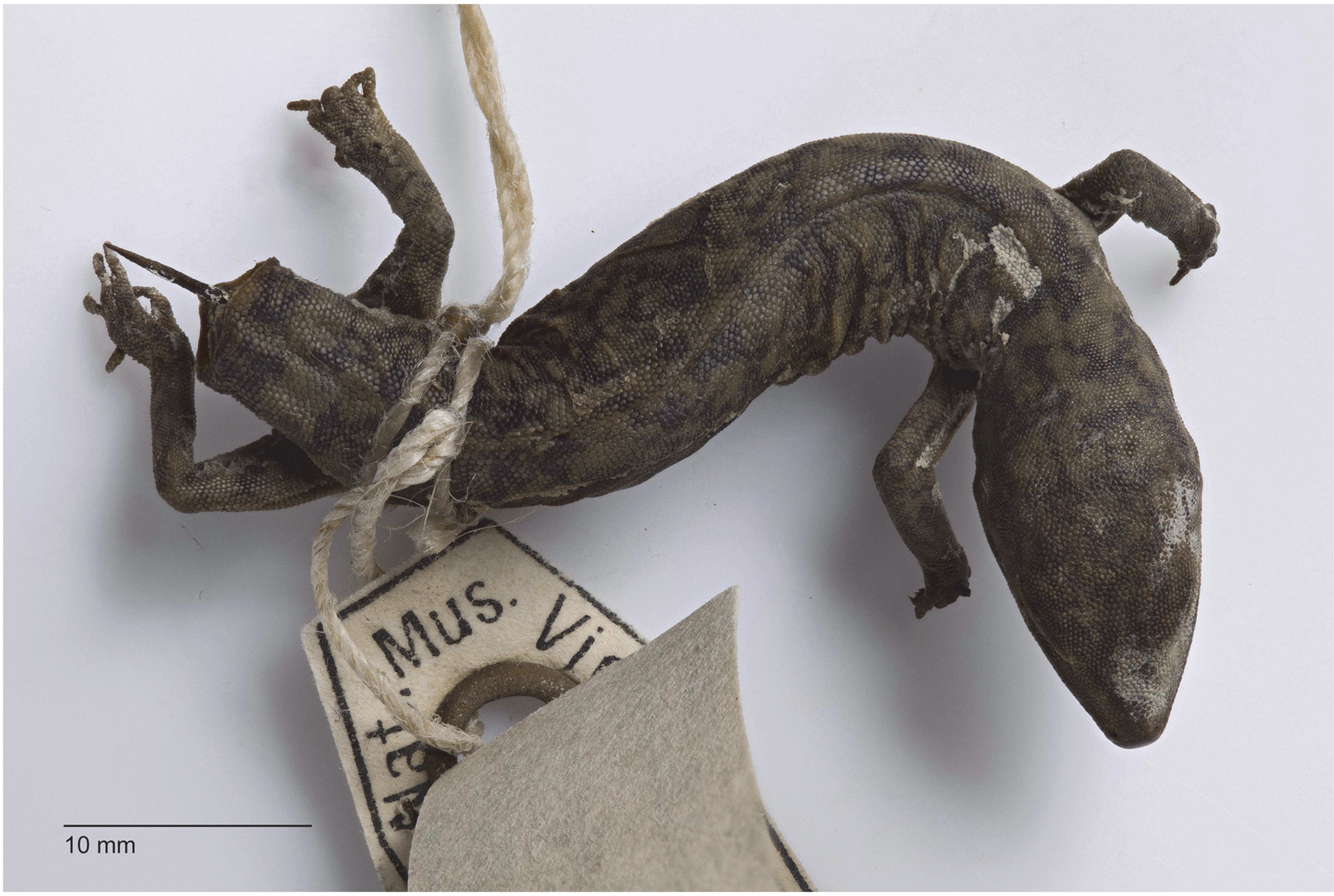

Fig 8. Lectotype of *D. conspicillatus* (NMV D7535). Charlotte Waters, Northern Territory. (Image—Katie Smith, NMV).

doi:10.1371/journal.pone.0111895.g008

in size on pectoral region; preanal pores absent; a small cluster of postanal tubercles present in both sexes but larger and more prominent in males **Limbs:** moderate; forelimb 27.67–34% SVL (n = 32, mean = 30.74, SD = 0.02), hindlimb 28.67–37.28% SVL (n = 32, mean = 33.50, SD = 0.02); digits short and squat, lacking any distal expansion; subdigital lamellae granular (not a clearly defined series except for small distal pair), 10–13 beneath fourth finger (n = 32, mean = 11.13, mode = 11, SD = 0.93), 11–16 beneath fourth toe (n = 32, mean = 12.72, mode = 12, SD = 1.22). **Original tail:** short, wide 39.7–56.4% tail length (n = 28, mean = 46.7, SD = 0.04), spade-like and bluntly pointed (lacking acute attenuated tip: Fig. 6A); scales large and plate-like, arranged in clear transverse pattern that usually incorporates rows of both large and small scales (Fig. 6A); larger scales with a short bluntly to sharply-tipped medial tubercle; 28–39 (n = 28, mean = 32.71, mode = 35, SD = 2.49) medial scale rows on tail from fracture plane (1st autotomy septum) to tip; 12–15 (n = 28, mean = 12.68, mode = 12, SD = 0.92) rows of scales across original tail (large row closest to maximum width); ventral scales considerably smaller than dorsal scales. **Regrown tail:** with rounded distal end and more uniform scalation that is not arranged in clear transverse rows.

Pattern (in spirit). Variable. Most specimens tan to mid-brown and heavily chequered with small dark blotches that may coalesce to produce a reticulated appearance (lighter individuals more uniform; mid-brown, finely peppered with darker markings and bearing pale spots on dorsal and lateral surfaces). Pale spots generally present, most prominent on flanks. In some

specimens there is reduced pigmentation on the vertebral zone producing as a ragged-edged vertebral stripe (one specimen, WAM R110770, has a well-defined dark vertebral stripe bordered on either side by a pale paravertebral stripe). Head generally with darker crown but paler towards periphery. A prominent, pale canthal stripe present, extending from anterior edge of orbit to tip of snout and producing a distinctive 'v' shaped marking which contrasts with the darker dorsal and lateral head markings. A broad dark zone on side of face extends posteriorly beyond eye to temporal region. A pale zone below eye extends to ear. Limbs mottled or spotted and inner digits of forelimb with reduced pigmentation. Ventral surfaces off-white, immaculate.

Comparisons. *Diplodactylus conspicillatus* is readily distinguished from *D. platyurus* in possessing an enlarged first supralabial that contacts the ventral edge of the nasal scale (*vs* 1st supralabial small and not differentiated from the rest of the supralabial row). It is distinguished from *D. barraganae* **sp. nov**. and *D. hillii* in having enlarged, plate-like mid-dorsal scales that are conspicuously larger than the dorsolateral scales (*vs* mid-dorsals small and granular, only slightly larger than dorsolateral scales). It is separated from the remaining three species in this complex (*D. laevis*, *D. bilybara* **sp. nov**. and *D. custos* **sp. nov**. by the shape of its original tail which is spade-like and lacks an acute attenuated tip (*vs* original tail bearing a short attenuated tip).

Distribution and Ecology. Very widely distributed throughout much of the arid zone; extending west to the eastern edge of the Pilbara and Western Australian Goldfields, east to Cunnamulla in south central Queensland, and south to the northern edge of the Nullarbor Plain in South Australia (Fig. 3). There are also scattered records from the Australian Monsoonal tropics in the Northern Territory, including two specimens from a high rainfall zone in north-eastern Arnhemland. Throughout this broad region this species inhabits a very wide range of habitats ranging from sparsely vegetated Gibber plains to open woodlands, but is generally associated with harder stony, clay and compacted sandy substrates.

Comments. Examination of the lectotype (NMV D7535: Fig. 8) shows it to be a poorly preserved specimen that looks to be slightly dessicated, is lacking a tail but has a rusted pin protuding from the tail base. Despite its poor condition, it clearly exhibits large plate-like scales on the vertebral region of its back accompanied by small, granular scales on the nape. The only other morphotype that occurs in the vicinity of the Northern Territory/South Australian border (*Diplodactylus laevis*) has large plate-like scales on both the vertebral region and the nape and is clearly not conspecific with NMV D7535.

The wide range of this species and observed deep phylogenetic structure suggests additional taxonomic investigations are necessary.

***Diplodactylus hillii* Longman 1915**

D. conspicillatus (in part; Kluge 1963)

D. conspicillatus (in part; Cogger, H.G. in Cogger *et. al.*, 1983)

'conspicillatus' D (Oliver et al. 2009)

Northern Fat-tailed gecko

Figs. 5D, 6C, 7C, 9

Material Examined. QM J1994, Port Darwin (13° 21' S, 130° 42' E) NT, **holotype**; NTM R17871, Arnhem Hwy, 5km E Corroboree Taven (12° 45' S, 131° 29' E) NT; NTM R9933, Reynolds River, Litchfield NP (13° 16' S, 130° 41' E) NT; NTM R17894, Kakadu NP, headwaters of Katherine River (13° 16' 12" S, 133° 00' 36" E) NT; NTM R20552, Stuart Hwy, Near Robin Falls (13° 21' S, 131° 08' E) NT; NTM R27363, Dorat Rd, Daly River Region (13° 30' S, 131°

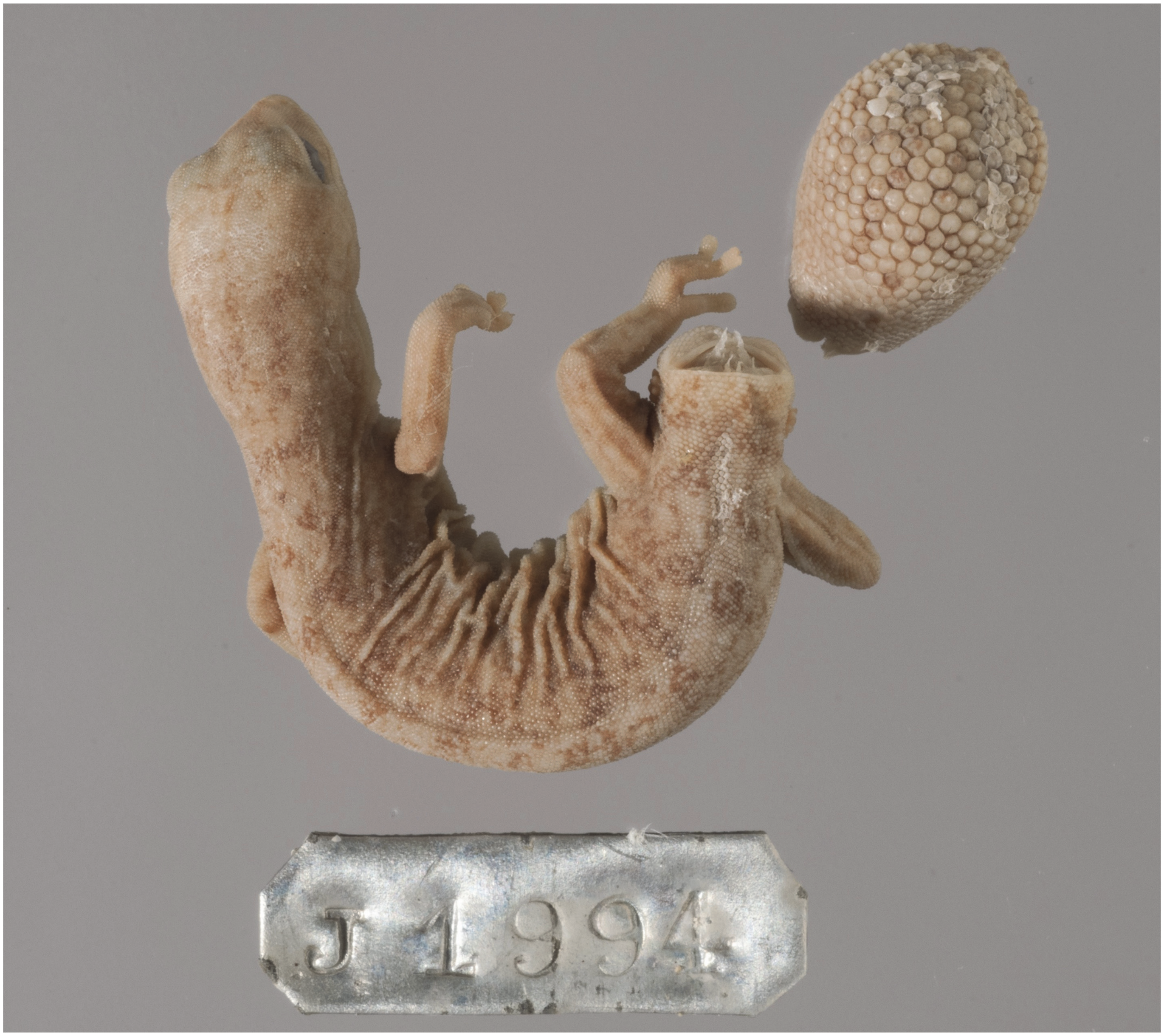

Fig 9. Holotype of *D. hillii* (QMJ 1994). Port Darwin, Northern Territory (Image—Jeff Wright, QM).

doi:10.1371/journal.pone.0111895.g009

17' E) NT; NTM R25027, Douglas Stn, Daly River Region (13° 31' 13" S, 131° 16' 09' E) NT; NTM R11690, Katherine Gorge NP (14° 18' 48" S, 132° 32' 54" E) NT; NTM R0802, Katherine, 10 Miles N (14° 22' S, 132° 19' E) NT; NTM R0152–53, Katherine, 2 miles N (14° 27' S, 132° 16' E) NT; NTM R3772–73, Manbuloo Stn (14° 31' S, 132° 12' E) NT; NTM R6300, Katherine, 40Km South (14° 39' S, 132° 26' E) NT; NTM R4514, S of Katherine, Stuart Hwy (14° 40' S, 132° 40' E) NT; NTM R0364–65, 5 miles N Katherine (14° 44' S, 132° 04' E) NT; NTM R24364, King River, Katherine (14° 46' 29" S, 132° 15' 10" E) NT; NTM R23310, Elsey NP (14° 54' 54" S, 133° 13' 45" E) NT.

Diagnosis. A moderate-sized member of the *D. conspicillatus* group (max SVL 52 mm) with a bold canthal stripe and greatly enlarged first supralabial (contacting ventral edge of nasal

scale). Mid-dorsal scales on trunk small and only slightly larger than dorsolateral scales. Original tail spade-like and lacking an acute attenuated extension at tip. Scales on dorsal surface of original tail all large and not arranged in clearly defined transverse rows (pine cone-like appearance).

Description. SVL mm 25.09–52.34 (n = 19, mean = 46.31, SD = 5.52). Proportions as % SVL: AG 43.87–56.20 (n = 19, mean = 48.50, SD = 0.03); T 30.59–39.32 (n = 19, mean = 34.42, SD = 0.03); HL 17.44–24.19 (n = 19, mean = 19.93, SD = 0.01). **Head:** moderate and not strongly differentiated from neck; snout longer than diameter of eye. HW 64.19–85.17% HL (n = 18, mean = 74.47, SD = 0.05); HD 43.8–54.4% HL (n = 19, mean = 47.9, SD = 0.03); S 44.62–50.47% HL (n = 19, mean = 47.0, SD = 0.01); EE 21.79–30.39% HL (n = 19, mean = 25.93, SD = 0.02); covered in small granular scales; rostral shield large and lacking a medial groove, hexagonal with 5 scales in contact with its posterior margin (n = 19); mental shield hemispherical, usually with a moderate process extending medially from its posterior margin, 8–12 scales contacting posterior edge (n = 19, mean = 10.11, mode = 11, SD = 1.10); supralabial scales 14–24 (n = 18, mean = 18.39, mode = 20, SD = 2.20) with the first enlarged and contacting ventral edge of nasal scale, the remaining series are small and not differentiated from the adjacent loreal scales; infralabial scales 13–21 (n = 16, mean = 17.38, mode = 19, SD = 2.50), all small and undifferentiated from adjacent chin scales; eye large, pupil vertical with crenulated margin; ear small, round to horizontally elliptic. **Neck:** broad with small granular scales on dorsal surface that are only slightly larger than the adjacent scales on the lateral surfaces. **Trunk:** moderate and somewhat stout; mid-dorsal scales only slightly larger than dorolateral scales (Fig. 5D); granules small on ventral surface but increase in size on pectoral region; preanal pores absent; a small cluster of postanal tubercles present in both sexes but larger and more prominent in males. **Limbs:** moderate; forelimb 29.19–36.03% SVL (n = 19, mean = 31.67, SD = 0.02); hindlimb 30.36–37.94% SVL (n = 18, mean = 34, SD = 0.02); digits moderate with no or only slight distal expansion; subdigital lamellae granular (not a clearly defined series except for small distal pair); 10–14 lamellae beneath fourth finger (n = 19, mean = 11.79, mode = 11, SD = 0.98); 12–15 lamellae beneath fourth toe (n = 19, mean = 13.11, mode = 13, SD = 0.94) **Original tail:** short, wide 40.35–72.49% tail length (n = 18, mean = 54.62, SD = 0.07); spade-like and bluntly pointed (lacking an acute attenuated tip; Fig. 6C); scales large and plate-like, not arranged in clearly defined transverse bands (scales more or less of uniform size; Fig. 6C) with short blunt to sharp medial tubercle; 23–30 (n = 19, mean = 25.58, mode = 25, SD = 2.27) medial scale rows on tail from fracture plane (1st autotomy septum) to tip; 10–15 (n = 18, mean = 11.83, mode = 12, SD = 1.50) rows of scales across original tail (large row at maximum width); ventral scales considerably smaller than dorsal scales. **Regrown tail:** not assessed but likely to be as for other species.

Measurements and scale counts of holotype. QM J 1994 (male, Fig. 9). SVL = 47.42mm, AG = 22.88mm, L1 = 13.84mm, L2 = 15.46mm, HL = 9.28mm, HD = 4.63mm, HW = 6.97mm, S = 4.42mm, EE = 2.43mm, TL = 14.52mm, TW = 9.35mm, scales contacting posterior edge of rostral = 5, scales contacting posterior edge of mental = 9, lamellae beneath 4th finger = 12, lamellae beneath 4th toe = 14, medial scale rows on tail from fracture plane (1st autotomy septum) to tip = 29, rows of scales across original tail 14, supralabials = 18, infralabials = 19.

Pattern (in spirit). Variable. Most specimens tan to mid-brown and heavily marked with dark, irregular bands that form a broad reticulum on upper lateral / paravertebral zone and may extend to lower flanks. Vertebral zone with a ragged dark edge; generally free of pattern but sometimes the dark flank pattern may bridge this zone or the vertebral line may carry a row of small dark blotches (some individuals with a finer, lighter reticulum over entire dorsal surface which is marked with numerous small pale spots). Head with a pale crown that is continuous with the vertebral zone. A pale canthal stripe present, extending from anterior edge of

orbit to tip of snout and producing a distinctive 'v' shaped marking which has dark edging. A poorly defined pale zone below eye extends to the ear. Limbs finely spotted. Inner digits with reduced pigmentation. Original tail with little pattern or with darker bars similar to those on flanks. Ventral surfaces off-white, immaculate.

Comparisons. *Diplodactylus hillii* is readily distinguished from *D. platyurus* in possessing an enlarged first supralabial that contacts the ventral edge of the nasal scale (*vs* 1st supralabial small and not differentiated from the rest of the supralabial row). It is distinguished from *D. conspicillatus*, *D. laevis*, *D. bilybara* **sp. nov**. and *D. custos* **sp. nov**. in having small mid-dorsal scales that are only slightly larger than the dorsolateral scales (*vs* mid-dorsal scales conspicuously larger than the smaller dorsolateral scales). It is further separated from *D. laevis*, *D. bilybara* **sp. nov**. and *D. custos* **sp. nov**. in lacking an acute attenuated tip to the original tail (*vs* attenuated tip present). *Diplodactylus hillii* most resembles *D. barraganae* **sp. nov**. with which it shares small mid-dorsal scales and a blunt, spade-like original tail. These two species differ most in the configuration of the scales on the original tail (enlarged scales not in clearly defined transverse rows and mostly subequal for *D. hillii vs* clearly defined trows of both large and small scales for *D. barraganae* **sp. nov**.).

Distribution and Ecology. Found in eastern and central "Top End", from close to Darwin south as far as Elsey National Park (Fig. 3). Its habitat preferences within this area have not been determined.

Comments. The holotype of *D. hillii*, (QMJ 1994; Fig. 9), was examined and exhibits a unique scale configuration found only in *D. conspicillatus sensu lato* populations from the N.T. occurring above 15° S (i.e. scales on dorsal surface of original tail all large and not arranged in clearly defined transverse rows).

***Diplodactylus laevis* (Sternfield, 1924)**

Gymnodactylus laevis Sternfield, 1924

D. conspicillatus (in part; Kluge 1967)

D. conspicillatus (in part; Cogger, H.G. in Cogger *et. al*., 1983)

'conspicillatus' C (Oliver et al. 2009)

Desert Fat-tailed gecko

Figs. 5B, 6B, 7D, 10

Material Examined. SMF8242, Hermannsburg Mission NT, **lectotype**; WAM R168509, Coulomb Point (17° 27' 44" S, 122° 09' 09" E) WA; NTM R32478–79, Tanami (19° 54' S, 130° 41' E) NT; NTM R32472, Lake Surprise (20° 0 6' S, 131° 00' 28" E) NT; WAM R161640, 12.5km NNE Goldsworthy (20° 14' 31" S, 119° 34' 28" E) WA; NTM R15137, NTM R15157, NTM R15191–92, Sangster's Bore, 12km SW (20° 52' S, 130° 16' E) NT; NTM R18018, Lake Mackay (22° 29' S, 129° 04' E) NT; WAM R166303, 3.2km N Pungkulpirri Waterhole (24° 37' 43" S, 128° 45' 20" E) WA; SAMA R36126, SAMA R36129, SAMA R36149, SAMA R36151, Yulara town site (25° 14' S, 131° 01' E) NT; SAMA R29928, 22km along Mulga Pk Rd SSE Curtin Springs homestead (25° 30' S, 131° 49' E) NT; SAMA R29936, 24km along Mulga Pk Rd SSE Curtin Springs homestead (25° 31' S, 131° 49' E) NT; SAMA R49965, 46.6km E of Purni Bore (26° 19' 18" S, 136° 33' 40" E) SA; SAMA R63874, 100.9km N Innamincka (26° 50' 16" S, 140° 40' 57" E) SA; SAMA R63894, 76.5km N Innamincka (27° 03' 03" S, 140° 40' 21" E) SA; SAMA R56481, 7.4km ESE (106 degrees) Mount Hoare (27° 04' 37" S, 129° 46' 22" E) SA; SAMA R56495 (27° 05' 03" S, 129° 44' 20" E) SA; SAMA R49077, SAMA R 49081, 1.7km NE of Candradecka Dam (27° 12' 04" S, 140° 52' 42" E) SA; WAM R172202, Great Victoria Desert

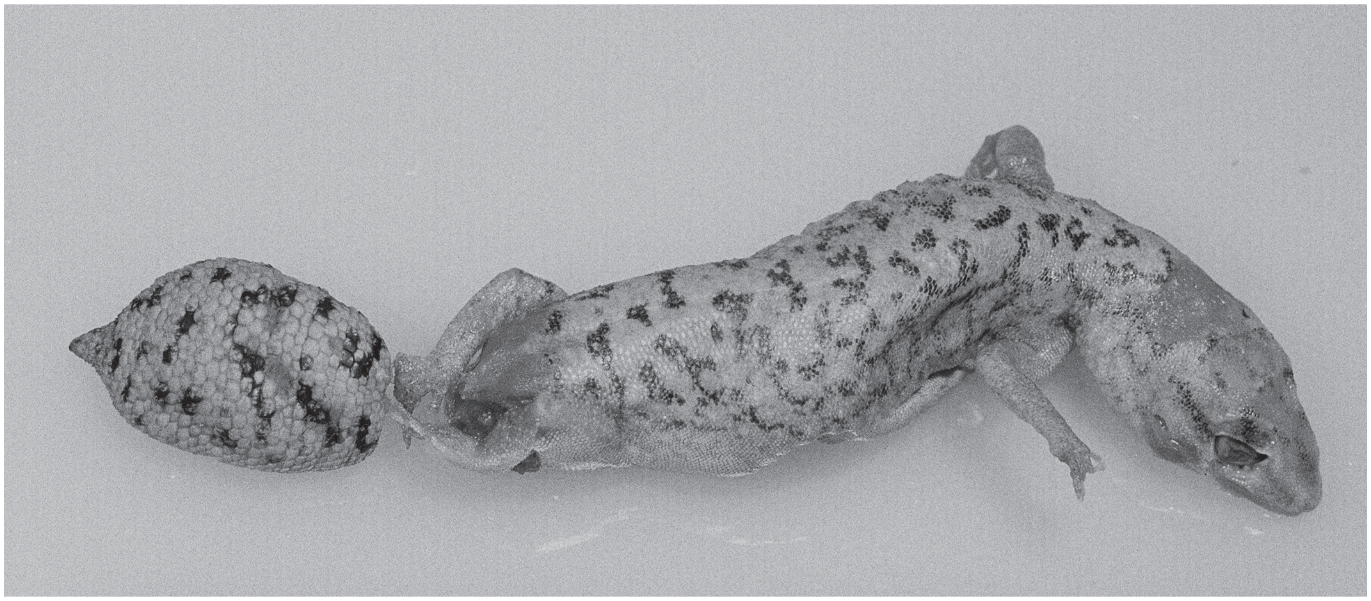

Fig 10. Lectotype of *Gymnodactylus laevis* (SMF8242). Hermannsburg Mission, Northern Territory. (Image: Dr Hal Cogger). This specimen was removed from the gut of a *Varanus gouldii* and is partially digested and in poor condition.

doi:10.1371/journal.pone.0111895.g010

(28° 20' 01" S, 127° 23' 50" E) WA; SAMA R62236, 184km SSW Wartaru (28° 30' 28" S, 129° 00' 17' E) SA; SAMA R62383, 164.7km SSE Wartaru (28° 31' 55" S, 129° 57' 27" E) SA; SAMA R62397, 164.7km SSE Wartaru (28° 32' 01" S, 129° 54' 43" E) SA; SAMA R62346, 166.7km SSE Wartaru (28° 32' 33" S, 130° 04' 18" E) SA; SAMA R57170, 11.2km E Vokes Hill Corner (28° 33' 42" S, 130° 47' 40" E) SA.

Diagnosis. A large member of the *D. conspicillatus* group (max SVL 65 mm) with a bold canthal stripe and a greatly enlarged first supralabial (contacting ventral edge of nasal scale). Mid-dorsal scales on trunk plate-like and markedly larger than smaller dorsolateral scales. Scales on nape and top of head also plate-like and whilst sometimes smaller than those on back, still considerably larger than the small granules on side of neck. Original tail sharply-pointed and terminating with an acute attenuated extension at tip. Scales on dorsal surface of tail arranged in transverse rows (which include rows of both large and small scales). Pattern generally reticulated.

Description. SVL mm 42.39–64.85 (n = 30, mean = 54.89, SD = 4.72). Proportions as % SVL: AG 46–57.31 (n = 30, mean = 51.82, SD = 0.03); T 36.73–52.26 (n = 26, mean = 45.72, SD = 0.04); HL 15.31–19.45 (n = 30, mean = 17.30, SD = 0.01). **Head:** moderate and not strongly differentiated from neck; snout longer than diameter of eye. HW 79.40–98.7% HL (n = 29, mean = 90. 90, SD = 0.05); HD 47.8–64% HL (n = 30, mean = 54.0, SD = 0.04); S 42.68–49.7% HL (n = 30, mean = 46, SD = 0.02); EE 24.2–34.6% HL (n = 30, mean = 29.64, SD = 0.03). Dorsal surface covered with enlarged scales that are continuous with the enlarged, plate-like dorsal scales on the trunk; rostral shield large and lacking a medial groove, hexagonal with 5–6 scales in contact with its posterior margin (n = 30, mean = 5.07, mode = 5, SD = 0.25); mental shield hemispherical but sometimes with a slight process extending medially from its posterior margin, 10–15 (n = 30, mean = 12.13, mode = 11, SD = 1.50) scales contacting posterior edge; supralabial scales 14–20 (n = 30, mean = 16.37, mode = 15, SD = 1.45) with the first enlarged and contacting ventral edge of nasal scale, the remaining series are small and not differentiated from the adjacent loreal scales; Infralabial scales 11–20 (n = 30, mean = 15.7, mode = 17, SD =

2.18), all small and undifferentiated from adjacent chin scales; eye large, pupil vertical with crenulated margin; ear small, round to horizontally elliptic. **Neck:** broad with enlarged scales on dorsal surface which are substantially larger than adjacent scales on the lateral surfaces (Fig. 5B). **Trunk:** moderate and somewhat stout; scales generally granular but a broad zone of larger, plate-like scales is present along mid-dorsum and these contrast in size with the smaller dorsolateral scales; granules small on ventral surface but increase in size on pectoral region; preanal pores absent; a small cluster of postanal tubercles present in both sexes but larger and more prominent in males **Limbs:** moderate; forelimb 25.08–33.9% SVL (n = 30, mean = 29.52, SD = 0.02); hindlimb 27.82–39% SVL (n = 30, mean = 32.17, SD = 0.03); digits short and squat, lacking any distal expansion; subdigital lamellae granular (not a clearly defined series except for small distal pair); 10–13 beneath fourth finger (n = 29, mean = 11.52, mode = 11, SD = 0.95); 11–16 beneath fourth toe (n = 29, mean = 13.59, mode = 13, SD = 1.12); **Original tail:** short, wide 34.4–58.5% tail length (n = 26, mean = 44.9, SD = 0.06), with an acute attenuated extension at tip (Fig. 6B); scales large and plate-like, arranged in clear transverse pattern that usually incorporates rows of both large and small scales (Fig. 6B); larger scales with short bluntly to sharply-tipped medial tubercle; 37–50 (n = 28, mean = 43.04, mode = 42, SD = 2.49) medial scale rows on tail from fracture plane (1st autotomy septum) to tip; 12–19 (n = 28, mean = 14.18, mode = 14, SD = 1.33) rows of scales across original tail (large row at maximum width); ventral scales considerably smaller than dorsal scales. **Regrown tail:** with rounded distal end and more uniform scalation that is not arranged in clear transverse rows.

Pattern (in spirit). Variable. Most specimens tan to mid-brown with a darker reticulated pattern of fine to moderate wavy lines that extend over the entire dorsum. Many specimens exhibit fine pale spotting that is most evident on the flanks. Head, as for body with dark reticulations on crown. A pale canthal stripe present, extending from anterior edge of orbit to tip of snout and producing a distinctive 'v' shaped marking which has dark edging. A broad dark zone on side of face extends posteriorly beyond eye to temporal region. A poorly to well-defined pale zone below eye extends to the ear. Limbs weakly mottled or spotted and inner digits with reduced pigmentation. Tail marked with small dark flecks. Ventral surfaces off-white, immaculate.

Comparisons. *Diplodactylus laevis* is readily distinguished from *D. platyurus* in possessing an enlarged first supralabial that contacts the ventral edge of the nasal scale (*vs* 1st supralabial small and not differentiated from the rest of the supralabial row). It is distinguished from *D. conspicillatus*, *D. laevis*, *D. bilybara* **sp. nov**. and *D. custos* **sp. nov**. in having enlarged, plate-like scales on the nape and top of head that are appreciably larger than those on the sides of the neck (*vs* scales on nape granular and not appreciably larger than those on sides of neck). It is most readily distinguished from *Diplodactylus hillii* and *D. barraganae* **sp. nov**. by the shape of its original tail which bears an acute attenuated extension at the tip (*vs* tail blunt, spade-like without an attenuated tip) and further distinguished from these species by its mid-dorsal scales (mid-dorsals enlarged and plate-like, conspicuously larger than the dorsolateral scales in *D. laevis vs* mid-dorsal scales small, only slightly larger than the dorsolaterals).

Distribution and Ecology. Widely distributed over much of the Australian arid zone, occurring from the Dampier Peninsula, Pilbara and Great Victoria Desert in the west, through much of north-western South Australia and the southern half of the Northern Territory, with an apparently isolated eastern population in the Channel Country around north-eastern South Australia (Fig. 3).

Comments. A black and white photographic image of the lectotype of *Gymnodactylus laevis* (SMF8242; Fig. 10) was kindly provided by Dr Harold Cogger. The specimen is damaged (partially digested) having been removed from the gut of a *Varanus gouldii* specimen from Hermannsburg Mission, NT. Despite its poor condition, it is possible to determine from the image

that the specimen has an enlarged 1st supralabial, enlarged scales on its mid-dorsum, nape and head, an acute attenuated extension at the tip of its original tail and some indication of a reticulated dorsal pattern. Cogger notes from his examination of the specimen that 'colour pattern is light brown or creamish with a series of irregular dark brown spots and patches forming a vague reticulum' (Cogger, unpublished data). This suite of characters fit the specimens examined above, whose distribution encompasses the central Australian region from which SMF8242 was collected. It remains unclear why Mertens [80] chose a partially digested specimen as the lectotype.

***Diplodactylus platyurus* Parker 1926**

D. conspicillatus (in part; Kluge 1967)

D. conspicillatus (in part; Cogger, H.G. in Cogger *et. al.*, 1983)

'conspicillatus' F–H (Oliver et al. 2009)

Eastern Fat-tailed gecko

Figs. 4B, 4D, 7E–F, 11

Material Examined. BMNH 1946.8.11.38, Torrens Ck (21°25'S, 145°14'E) QLD, **holotype;** QM J71803, 15km N Mt Carbine towards Laura (16° 30' S, 145° 03' E) QLD; QM J91172, near Mt Carbine township (16° 31' 51" S, 145° 06' 18" E) QLD; QM J69713, Fossilbrook, Burlington Stn (17° 48' 30" S, 144° 23' 30" E) QLD; QM J58912, Normanton, 8.2km W of, on Cloncurry Rd (17° 44' S, 141° 02' E) QLD; QM J58919, Normanton, 16.0km West of, on the Cloncurry Rd

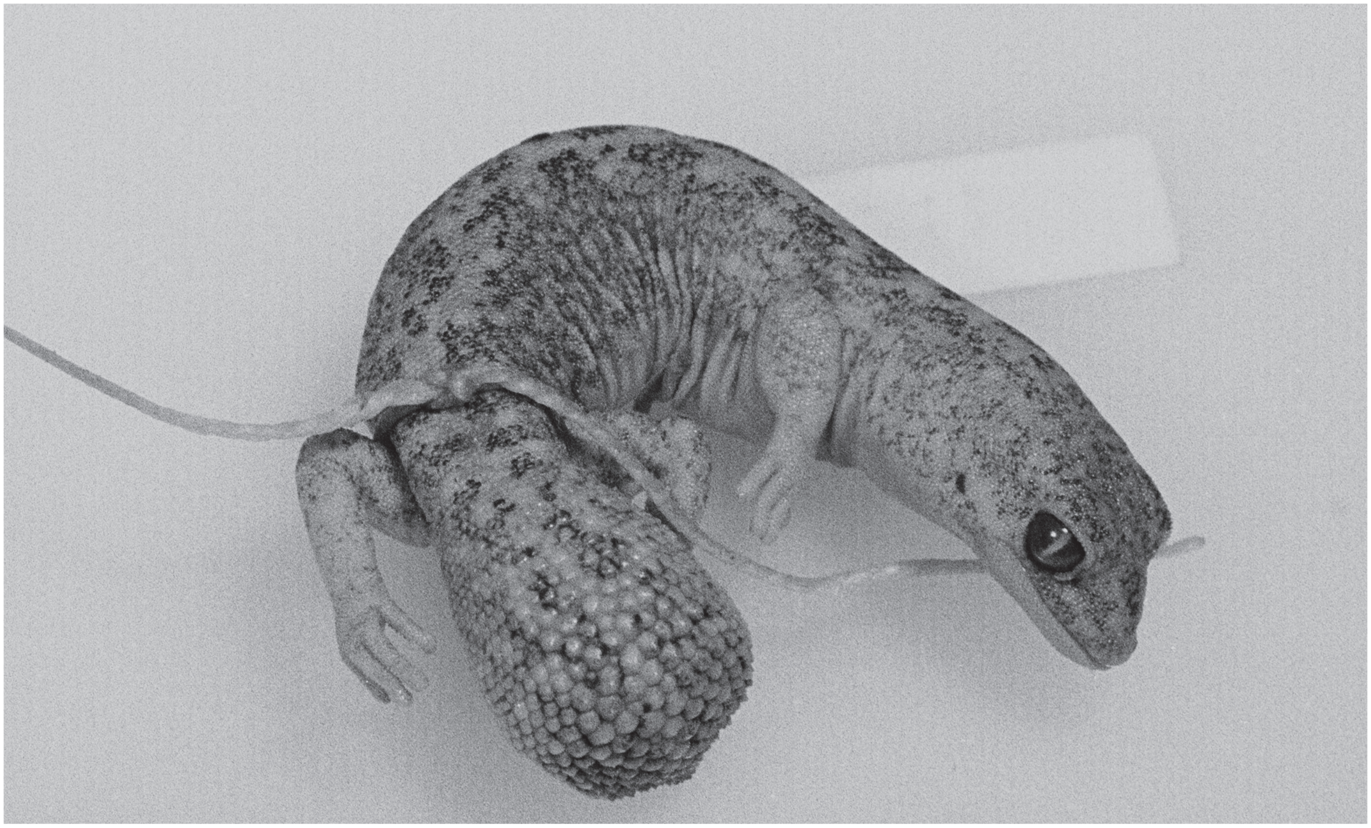

Fig 11. Holotype of *D. platyurus* (BMNH 1946.8.11.38). Torrens Ck, Queensland. (Image: Dr Hal Cogger).

doi:10.1371/journal.pone.0111895.g011

(17° 48' S, 141° 01' E) QLD; QM J77374, Florey St, Wulguru, Townsville (19° 20' S, 146° 49' E) QLD; QM J92286, Mingela Road near Townsville ~(19°53'S, 146°38'E) QLD; SAMA R63337, Mingela (19° 52' 12" S, 146° 37' 48" E) QLD; QM J82303, Blackbraes NP (19° 23' 31" S, 144° 09' 01" E) QLD; QM J80633, Blackbraes NP (19° 23' 57" S, 144° 09' 03" E) QLD; QM J63337, Porcupine Gorge NP (20° 23' S, 144° 26'E) QLD; QM J47527, Torrens Ck, 12km NNE (20° 39' S, 145° 05'E) QLD; QM J54321, Hughenden, 9.6km NE (20° 47' 48" S, 144° 20' E) QLD; QM J44369, Dalrymple town reserve 107, Freehold Portion (20° 57' S, 147° 05'E) QLD; QM J81437, Morrinya NP (21° 23' S, 144° 58' E) QLD; QM J81752, Lenton Downs (21° 33' 23" S, 148° 08' 24" E) QLD; QM J68966, BHP S Walker Ck Coal Mine, 40km W Nebo (21° 44' 36" S, 148° 24' 50" E) QLD; QM J81306, 27.5km N Moranbah (21° 46' S, 148° 00' E) QLD; QM J69765, Moranbah, 5km S (22° 02' S, 148° 03' E) QLD; SAMA R63336, Winton, (22° 27' S, 142° 57' E) QLD; QM J92287, Winton (22° 28' 42'S, 142° 53' 31"E) QLD; QM J63083, Blair Athol Coal Mine (22° 42' S, 147° 33' S) QLD; QM J78200, Junee SF9 (22° 48' 24" S, 149° 59'56' E) QLD; QM J45804, Bluff, 1.8km E (23° 35' S, 149° 06' E) QLD; QM J83120, Blackwater, 21.3km SW (23° 43' 50" S, 148° 44' 37" E) QLD; QM J47655, Blackdown Tableland NP, The Gap (23° 48' S, 149° 08'E) QLD; QM J90778, Noonbah homestead, 3.4km NNE (24° 04' 51" S, 143° 11' 54" E) QLD; AMS R60250, 37km N of Blackall on Landsborough QLD (24° 08' S, 145° 21' E) QLD; QM J56888, Waterloo site 1 (24° 16' S, 143° 13' E) QLD; QM J89191, Tyrone, approx 70km NW of Charleville—3km S of old north Tyrone homestead(25° 58' 55" S, 145° 44' 17" E) QLD; QM J35697, Ambathala NRS, 1km S Ra Tank (26° 01' 30" S, 145° 04' 30" E) QLD; QM J74874, Mariala Nature Ref. Site, No. 3 hut (26° 04' 48" S, 145° 06' E) QLD; QM J79909, Mariala (26° 05' 30" S, 145° 04' 15" E) QLD; AMS R158426, Sturt NP, Silver City Hwy., Wittabrinna Ck. Crossing (29° 22' 38" S, 142° 02' 08" E) NSW; AMS R132996, Wanaaring, 4km W of Wanaaring at Turnoff To Wilcannia (29° 42' S, 144° 07' E) NSW; AMS R165698, Nocoleche Nature Reserve, 11km West of Wanaaring—Wilcannia Rd (29° 52' 08" S, 144° 00' 34" E) NSW; AMS R162733, Lake Peery NP (30° 43' 28" S, 143° 29' 15" E) NSW.

Diagnosis. A large member of the *D. conspicillatus* group (max SVL 60 mm) lacking a well-defined canthal stripe and without a greatly enlarged first supralabial (first supralabial not in contact with ventral edge of nasal scale). Dorsal scales on trunk plate-like and markedly larger than smaller dorsolateral scales. Scales on nape granular and only slightly larger than granules on side of neck. Scales on dorsal surface of tail arranged in transverse rows (often of uniform size but can include rows of both large and small scales). Pattern generally with dark, heavily spotted flanks and a series of pale vertebral blotches or a continuous pale vertebral zone.

Description. SVL mm 40.55–60.21 (n = 36, mean = 48.71, SD = 5.20). Proportions as % SVL: AG 42.39–56.37 (n = 32, mean = 48.83, SD = 0.03); T 28.38–42.56 (n = 30, mean = 35.67, SD = 0.03); HL 17.38–22.41 (n = 36, mean = 19.74, SD = 0.01). **Head:** moderate and not strongly differentiated from neck; snout longer than diameter of eye. HW 73.3–89.41% HL (n = 33, mean = 81.53, SD = 0.04); HD 36–54.44% HL (n = 33, mean = 47.1, SD = 0.05); S 42.02–47.61% HL (n = 37, mean = 45.01, SD = 0.01); EE 23.42–32.78% HL (n = 33, mean = 26.84, SD = 0.02); covered in small granular scales; rostral shield large and lacking a medial groove, hexagonal with 5–13 scales in contact with its posterior margin (n = 35, mean = 8.69, mode = 9, SD = 1.68); mental shield hemispherical, usually with a moderate process extending medially from its posterior margin, 8–15 scales contacting posterior edge (n = 36, mean = 10.58, mode = 11, SD = 1.54); supralabial scales 11–19 (n = 37, mean = 15.51, mode = 15, SD = 1.61), the first not enlarged and subequal with the rest of the supralabial row which are not differentiated from the adjacent loreal scales (Fig. 4D); infralabial scales 12–20 (n = 37, mean = 15.76, mode = 16, SD = 1.69), all small and undifferentiated from adjacent chin scales; eye large, pupil vertical with crenulated margin; ear small, round to horizontally elliptic. **Neck:** broad with small granular scales on dorsal surface that are only slightly larger than the adjacent scales on

the lateral surfaces. **Trunk:** moderate and somewhat stout; scales of dorsum plate-like and markedly larger than smaller granules on flanks; granules small on ventral surface but increase in size on pectoral region; preanal pores absent; a small cluster of postanal tubercles present in both sexes but larger and more prominent in males. **Limbs:** moderate; forelimb 28.11–37.26% SVL (n = 34, mean = 32.43, SD = 0.02); hindlimb 30.61–40.76% SVL (n = 35, mean = 35.80, SD = 0.03); digits moderate with no or only slight distal expansion; subdigital lamellae granular (not a clearly defined series except for small distal pair); 9–13 lamellae beneath fourth finger (n = 35, mean = 10.94, mode = 10, SD = 1.11); 9–15 lamellae beneath fourth toe (n = 36, mean = 11.86, mode = 12, SD = 1.20). **Original tail:** short, wide 44.34–78.82% tail length (n = 30, mean = 60.81, SD = 0.08); spade-like and bluntly pointed (lacking an acute attenuated tip); scales arranged in clear transverse bands which incorporate rows of both large and small scales (or consist of scales that are more or less uniform in size), each with a short blunt to sharp medial tubercle; 20–34 (n = 33, mean = 25.06, mode = 26, SD = 3.16) medial scale rows on tail from fracture plane (1st autotomy septum) to tip; 10–18 (n = 33, mean = 13.24, mode = 13, SD = 1.50) rows of scales across original tail (large row at maximum width); ventral scales considerably smaller than dorsal scales. **Regrown tail:** with rounded distal end and more uniform scalation that is not arranged in clear transverse rows.

Pattern (in spirit). Variable. Most specimens tan to mid-brown with varying degrees of spotting; most prominent on flanks. Dorsum with an overlay of fine, dark reticulations or a more solid dark pattern. Vertebral zone with reduced pigment but often broken by transverse bars, isolating a series irregular pale blotches along back. In some specimens the vertebral zone is largely unpatterned and has a wavy edge where it borders the darker paravertebral zone. Head, as for dorsal ground colour with scattered dark flecks or blotches. Canthal stripe absent or very weak without sharply defined edges and not contrasting strongly with other facial markings. Limbs with fine reticulations, inner digits of forelimb with reduced pigmentation. Ventral surfaces off-white, immaculate.

Comparisons. *D. platyurus* is readily distinguished from *D. conspicillatus*, *D. laevis*, *D. hillii*, *D. bilybara* **sp. nov.,** *D. custos* **sp. nov**. and *D. barraganae* **sp. nov**. by the condition of the 1st supralabial (small and not differentiated from the rest of the supralabial row in *D. platyurus vs* greatly enlarged and contacting ventral edge of nasal scale) and by the absence of a well-defined canthal stripe (*vs* canthal stripe well-developed).

Distribution and Ecology. Occurs over much of eastern and central Queensland, from the Normanton and around Cairns in the north, south to around Rockhampton in the east, and throughout much of the channel country to west of the Great Dividing Range, extending south as far as north-west New South Wales and north-east South Australia (Fig. 3). Occurs in subhumid to arid woodland habitats on a range of sand and clay based substrates (A. Emmott pers. com).

Comments. A black and white photographic image of the holotype of *Diplodactylus platyurus* (BMNH 1946.8.11.38; Fig. 11) was kindly provided by Dr Harold Cogger. The specimen, from Torrens Ck, Qld (21°25'S, 145°14'E) has an undifferentiated supralabial row (i.e. no enlarged supralabials) a character that is only found in the most easterly populations of the *D. conspicillatus* group occurring in Queensland and NSW. A specimen from Torrens Ck, QM J47527 (the type locality), displaying this character is included in the material examined.

The taxonomic assignment of two specimens from the Edward River region on western Cape York Peninsula (QM J58251 Melon Yard, Strathgordon H, 14°43'12"S, 142°18'E and QM J81110 Edward River, 14°24'36"S, 142°09'36"E) remains unresolved. Whilst this population is geographically most proximate to *D. platyurus*, these specimens have an enlarged 1st supralabial and may represent an additional taxon not included in our limited genetic sampling.

Diplodactylus barraganae **Couper, Oliver & Pepper sp. nov**.

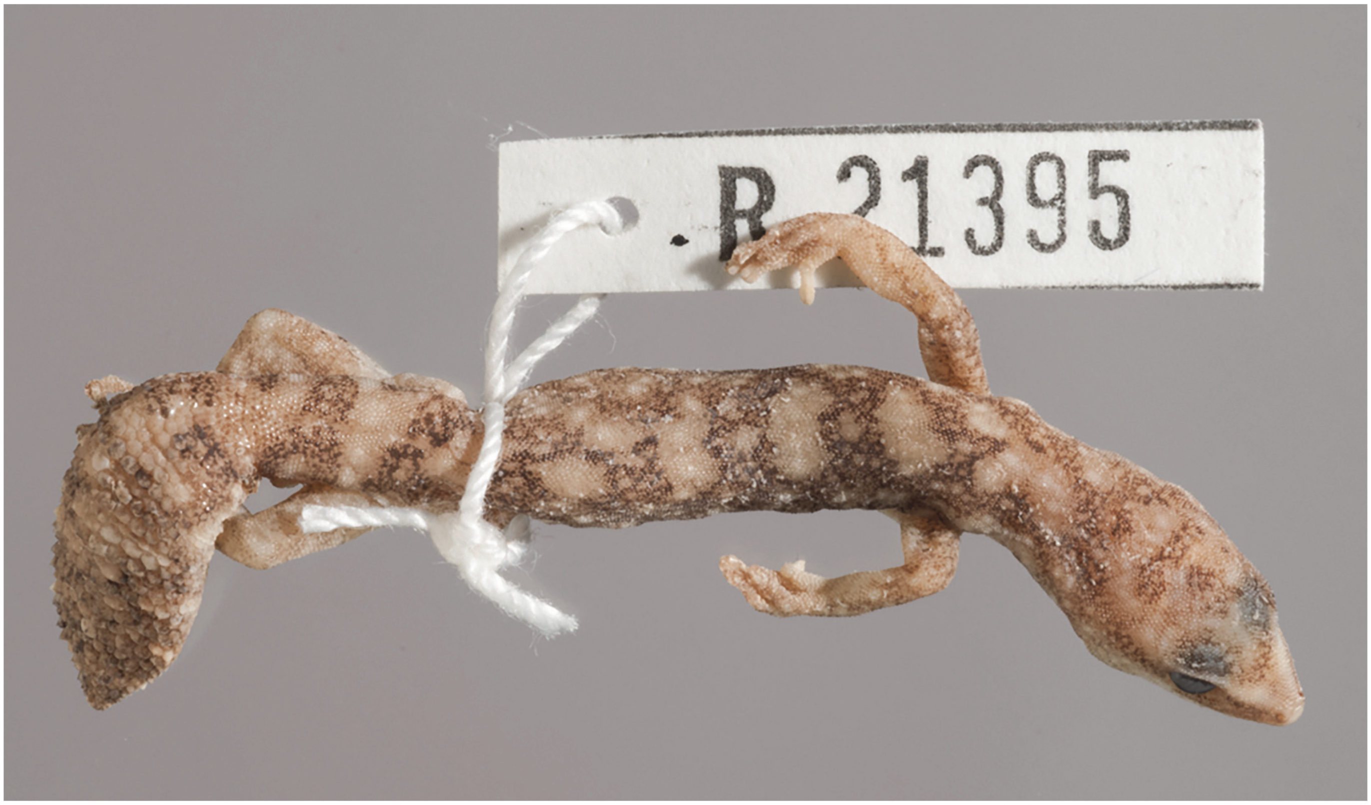

Fig 12. Holotype of *D. barraganae* sp. nov. (NTM R21395). Musselbrook Reserve, Border Waterhole, Northern Territory/Queensland border. (Image: Jeff Wright).

doi:10.1371/journal.pone.0111895.g012

urn:lsid:zoobank.org:act:6DEE15D3-30FA-4C8F-89AF-9FB06DB43480

'conspicillatus' A (Oliver et al. 2009)

Gulf Fat-tailed gecko

Figs. 6D, 12

Holotype. NTM R21395, Musselbrook Reserve, Border Waterhole (18° 36' 30" S, 137° 59' 18" E) NT/QLD border.

Paratypes. NTM R21886, NTM R21892, Sherwin Ck / Roper River Junction (14° 40' S, 134° 22' E) NT; NTM R21088, Carpentaria Hwy, 100km E Stuart Hwy (16° 25' 35" S, 134° 10' 48" E) NT; NTM R20606, Cape Crawford Area (16° 42' 07" S, 135° 31' 04" E) NT; NTM R20605, Cape Crawford Area (16° 53' 42" S, 135° 40' 31" E) NT; QM J11035–37, Doomadgee Mission Stn (17° 55' 48" S, 138° 49' 12" E) QLD; QM J51987, Lawn Hill NP (18° 42' 30" S, 138° 28' 30" E) QLD; QM J75143, Lawn Hill (18° 42' 30" S, 138° 28' 48" E) QLD; QM J52723, Lawn Hill Stn, Century Project Site (18° 45' S, 138° 35" E) QLD; AMS R162275, Riversleigh World Heritage Area (19° 00' 11" S, 138° 40' 03" E) QLD; QM J85474, Riversleigh (19° 00' 47' S, 138° 40' 06" E) QLD; QM J49251, Gregory R, nr 'Rackham's Roost', Riversleigh Stn (19° 02' S, 138° 45' E) QLD; AMS R17974 –, 75, Mount Isa (20° 44' S, 139° 29' E) QLD.

Etymology. Named for María Elena Barragán (Fundacion Herpetologica Gustavo Orces. Quito, Ecuador) in recognition of her contributions to reptile conservation and public education.

Diagnosis. A small member of the *D. conspicillatus* group (max SVL 49 mm) with a bold canthal stripe and greatly enlarged first supralabial (contacting ventral edge of nasal scale). Mid-dorsal scales on trunk small and only slightly larger than the dorsolaterals. Original tail spade-like and lacking an acute attenuated extension at tip. Scales on dorsal surface of tail arranged in transverse rows (which include rows of both large and small scales). Pattern not strongly contrasting, usually some indication of a pale, jagged-edged vertebral zone.

Description. SVL mm 25.05–49.47 (n = 17, mean = 41.75, SD = 6.80). Proportions as % SVL: AG 45.12–55.81 (n = 17, mean = 49.85, SD = 0.03); T 30.12–43.32 (n = 14, mean = 36.70, SD = 0.03); HL 17.83–22.12 (n = 17, mean = 20.11, SD = 0.01). **Head:** moderate and not strongly differentiated from neck; snout longer than diameter of eye. HW 73.1–92.91% HL (n = 17, mean = 81.1, SD = 0.06); HD 40.1–53.4% HL (n = 17, mean = 47.7, SD = 0.04); S 42.66–53.43% HL (n = 17, mean = 46.54, SD = 0.02); EE 24.8–30.43% HL (n = 17, mean = 27, SD = 0.02). Covered in small granular scales; rostral shield large and lacking a medial groove, hexagonal with 5–6 scales in contact with its posterior margin (n = 17, mean = 5.12, mode = 5, SD = 0.40); mental shield hemispherical, usually with a moderate process extending medially from its posterior margin, 10–14 scales contacting posterior edge (n = 17, mean = 11.41, mode = 11, SD = 1.10); supralabial scales 15–19 (n = 17, mean = 17.35, mode = 19, SD = 1.42) with the first enlarged and contacting ventral edge of nasal scale, the remaining series are small and not differentiated from the adjacent loreal scales; Infralabial scales 13–18 (n = 17, mean = 16.24, mode = 16, SD = 1.01), all small and undifferentiated from adjacent chin scales; eye large, pupil vertical with crenulated margin; ear small, round to horizontally or vertically elliptic. **Neck:** broad with small granular scales on dorsal surface that are only slightly larger than the adjacent scales on the lateral surfaces. **Trunk:** moderate and somewhat stout; scales of dorsum small, only slightly larger than dorsolateral scales; granules small on ventral surface but increase in size on pectoral region; preanal pores absent; a small cluster of postanal tubercles present in both sexes but larger and more prominent in males **Limbs:** moderate; forelimb 29.83–35% SVL (n = 17, mean = 32.80, SD = 0.02); hindlimb 33.04–38.76% SVL (n = 17, mean = 36.03, SD = 0.02); digits moderate with slight distal expansion; subdigital lamellae granular (not a clearly defined series except for small distal pair); 8–14 beneath fourth finger (n = 17, mean = 10.94, mode = 11, SD = 1.37); 10–15 beneath fourth toe (n = 17, mean = 12.18, mode = 13, SD = 1.35); **Original tail:** short, wide 46.81–63.84% tail length (n = 9, mean = 54.58, SD = 0.07), spade-like and bluntly pointed (lacking an acute attenuated extension at tip; Fig. 6D); scales large and plate-like, arranged in clear transverse pattern that usually incorporates rows of both large and small scales (Fig. 6D); larger scales with short bluntly to sharply- tipped medial tubercle; 31–39 (n = 14, mean = 34.92, mode = 36, SD = 2.43) medial scale rows on tail from fracture plane (1st autotomy septum) to tip; 12–16 (n = 14, mean = 13.93, mode = 15, SD = 1.44) rows of scales across original tail (large row at maximum width); ventral scales considerably smaller than dorsal scales. **Regrown tail:** with rounded distal end and more uniform scalation that is not arranged in clear transverse rows.

Measurements and scale counts of holotype. NTM R21395 (male, Fig. 12) SVL = 40.58mm, AG = 18.31mm, L1 = 13.09mm, L2 = 13.94mm, HL = 8.25mm, HD = 3.33mm, HW = 6.38mm, S = 3.71mm, EE = 2.14mm, TL = 14.77mm, TW = 7.2mm, scales contacting posterior edge of rostral = 6, scales contacting posterior edge of mental = 11, lamellae beneath 4th finger = 10, lamellae beneath 4th toe = 11, medial scale rows on tail from fracture plane (1st autotomy septum) to tip = 36, rows of scales across original tail 16, supralabials = 16, infralabials = 16.

Pattern (in spirit). Tan to mid-brown, suffused with darker pigment on back and flanks. Pattern incorporates diffuse spotting and obscure reticulations and a pale, continuous or broken, vertebral zone. Head with numerous dark scales that often form a fine netted pattern. A

moderately well-developed pale canthal stripe present, extending from anterior edge of orbit to tip of snout and producing a distinctive 'v' shaped marking. A diffuse dark zone on side of face extends posteriorly beyond eye to temporal region. Limbs obscurely marked with vague spotting or netted pattern and inner digits of fore and hindlimb with reduced pigmentation. Ventral surfaces off- white, immaculate.

Comparisons. *Diplodactylus barraganae* **sp. nov**. is readily distinguished from *D. platyurus* in possessing an enlarged first supralabial that contacts the ventral edge of the nasal scale (*vs* 1st supralabial small and not differentiated from the rest of the supralabial row). It is distinguished from *D. conspicillatus*, *D. laevis*, *D. bilybara* **sp. nov**. and *D. custos* **sp. nov**. in having small mid-dorsal scales that are only slightly larger than the dorsolaterals (*vs* mid-dorsals enlarged and plate-like, conspicuously larger than the dorsolaterals) and further distinguished from *D. laevis*, *D. bilybara* **sp. nov**. and *D. custos* **sp. nov**. by the shape of the original tail (tail blunt, spade-like without an acute attenuated extension at tip in *D. barraganae* **sp. nov**. *vs* tail with an acute attenuated extension at tip).

Distribution and Ecology. Occurs over a broad band along the southern edge of the Gulf of Carpentaria, from the Roper River region in the northwest, east and south as far as Mt Isa (Fig. 3). The holotype was collected in 'open woodland on red sandy soil' (P. Horner pers. com.).

Diplodactylus bilybara **Couper, Pepper & Oliver sp. nov**.

urn:lsid:zoobank.org:act:0405E99F-8082-4DA3-851B-6872D674A414

'conspicillatus' B (Oliver et al. 2009)

Western Fat-tailed Gecko

Figs. 6E, 7G, 13

Holotype. WAM R174500 (formerly SAMA R22820), 21km S Barradale (22° 55' S, 114° 46'E) WA.

Paratypes. WAM R132531-32, Burrup Peninsula (20° 40' 36" S, 116° 45' 08" E) WA; WAM R132529, Burrup Peninsula (20° 40' 49" S, 116° 44' 37" E) WA; WAM R110058, 3.5km S Karratha (20° 46' 04" S, 116° 50' 31" E) WA; WAM R110027, 6km S Karratha (20° 47' 40" S, 116° 51' 24" E) WA; WAM R165155, 8.5km WSW Yanyare River mouth (20° 50' 40" S 116° 22' 02" E) WA; WAM R159892, WAM R159894, WAM R159940, WAMR159947, 10km S Mallina homestead (20° 58' 10" S, 118° 02' 54" E) WA; WAM R165177, 9.5km ESE Marda Pool (21° 03' 47" S, 116° 14' 00" E) WA; WAM R110182, WAM R110218, WAM R110220, 12.5km SW Millstream (21° 40' 37" S, 116° 58' 30" E) WA; WAM R134523, 8km N Exmouth (21° 52' 12" S, 114° 07' 01" E) WA; WAM R110148, 8km S Coolawanyah (21° 52' 55" S, 117° 47' 40" E) WA; WAM R163018, 7km SSE Mount Minnie (22° 10' 10" S, 115° 33' 39" E) WA; WAM R162059, 19.5km SSW Mount Amy (22° 25' 09" S, 115° 50' 16" E) WA; WAM R162052-53 21km SSE Mount Amy (22° 26' 05" S, 115° 55' 49" E) WA; WAM R158331, WAM R159932, Giralia homestead (22° 41' 38" S, 114° 23' 28" E) WA; SAMA R22905, 6km S Barradale (22° 52' S, 114° 52' E) WA; SAMA R22818, 11km S Barradale (22° 52' 30" S, 114° 50' E) WA; SAMA R22819, 9km S Barradale (22° 53' S,114° 52' E) WA; SAMA R22821 21km S Barradale (22° 55' S, 114° 46'E) WA; AMS R165713, Jack Hills (26° 03' 24" S, 117° 12' 58" E) WA.

Etymology. Refers to the Pilbara region where this species occurs. The name Pilbara is said to be derived from the Aboriginal word *bilybara*, meaning 'dry' in the languages of the Nyamal and Banyjima people.

Diagnosis. A large member of the *D. conspicillatus* group (max SVL 63 mm) with a well-defined canthal stripe and a greatly enlarged first supralabial (first supralabial contacts ventral

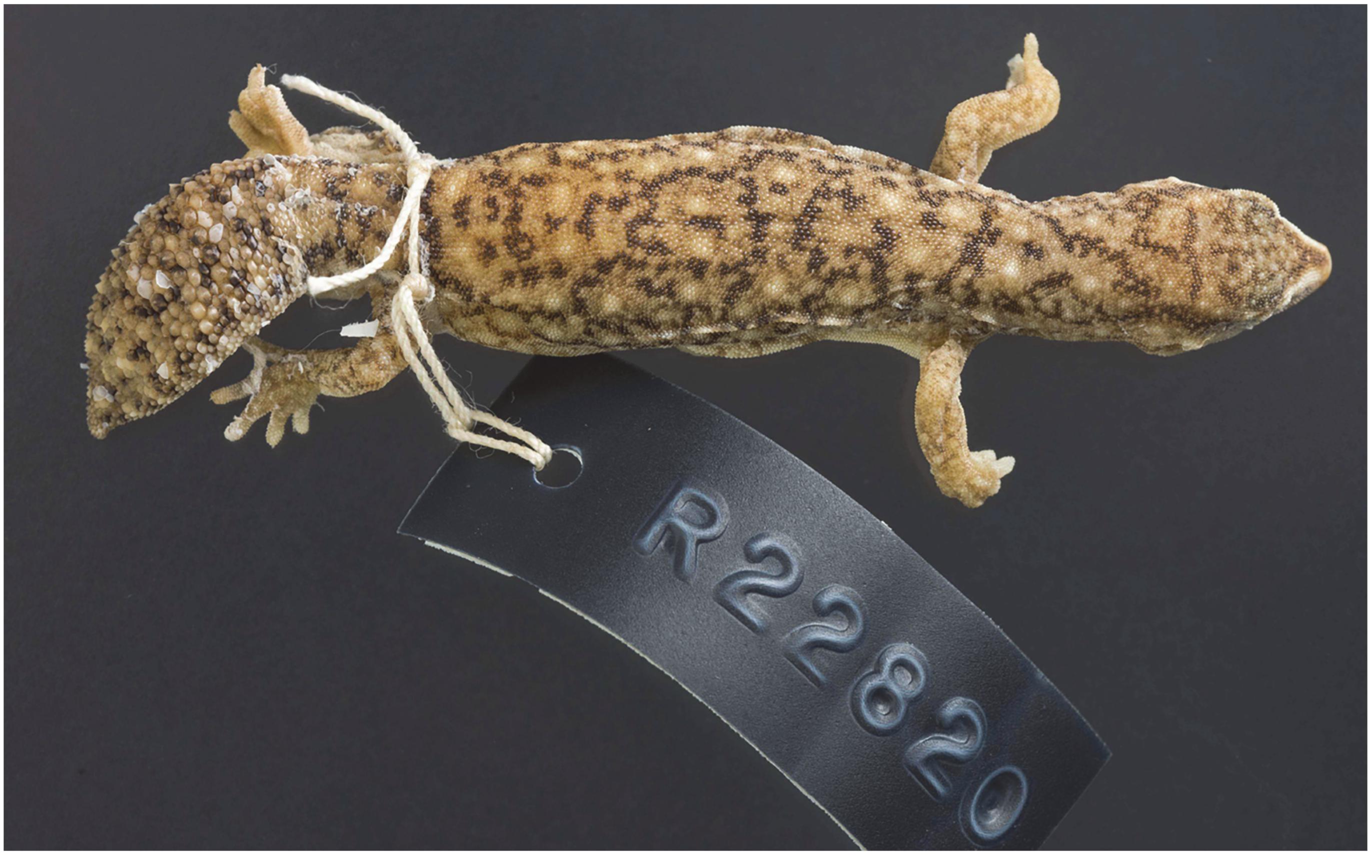

Fig 13. Holotype of *Diplodactylus bilybara* sp. nov. (WAM R174500). 21km south of Barradale, Western Australia. (Image: Peter Waddington, QM).

doi:10.1371/journal.pone.0111895.g013

edge of nasal scale). Dorsal scales on trunk plate-like and markedly larger than smaller dorso-laterals. Scales on nape granular and only slightly larger than granules on side of neck. Original tail with a short to moderate, acute attenuated extension at tip; scales on dorsal surface of tail arranged in transverse rows (often in a pattern of one large row followed by two small rows; scales in the small rows ∼ ¼ the size of the scales in the adjacent large rows. Pattern variable; reticulated or with obscure transverse bands and generally incorporating numerous small pale spots. Dark pigment on crown and snout contrast markedly with pale canthal stripe and lower jaw colour which extends posteriorly as a pale bar towards the ear opening.

Description. SVL mm 39.2–60.8 (n = 28, mean = 48.83, SD = 5.20). Proportions as % SVL: AG 44.60–54.16 (n = 27, mean = 49.64, SD = 0.03); T 33.88–48.34 (n = 22, mean = 43.86, SD = 0.03); HL 15.9–21.04 (n = 28, mean = 18.57, SD = 0.01). **Head:** moderate and not strongly differentiated from neck; snout longer than diameter of eye. HW 79.89–92.08% HL (n = 28, mean = 87.05, SD = 0.03); HD 43.7–55.7% HL (n = 28, mean = 49.8, SD = 0.02); S 43.31–47.87% HL (n = 28, mean = 45.26, SD = 0.01); EE 24.45–33.54% HL (n = 28, mean = 28.23, SD = 0.02). Covered in small granular scales; rostral shield large and lacking a medial groove, hexagonal 5–6 scales contacting posterior edge of rostral scale (n = 28, mean = 5.29, mode = 5, SD = 0.46); mental scale usually without (or with only a slight) process on medial posterior margin, 9–14 scales contacting posterior edge (n = 28, mean = 11.29, mode = 12, SD = 1.21); supralabial scales 13–20 (n = 27, mean = 16.78, mode = 17, SD = 1.87), with the first enlarged and contacting ventral edge of nasal scale, the remaining series are small and not differentiated from the adjacent loreal scales; infralabial scales 12–21 (n = 27, mean = 16.96, mode = 16, SD = 2.70), all

small and undifferentiated from adjacent chin scales; eye large, pupil vertical with crenulated margin; ear small, round to horizontally elliptic. **Neck:** broad with small granular scales on dorsal surface that are only slightly larger than the adjacent scales on the lateral surfaces. **Trunk:** moderate and somewhat stout; scales of mid-dorsum plate-like and markedly larger than dorsolateral scales; granules small on ventral surface but increase in size on pectoral region; preanal pores absent; a small cluster of postanal tubercles present in both sexes but larger and more prominent in males. **Limbs:** moderate; forelimb 24.87–35.85% SVL (n = 28, mean = 31.13, SD = 0.02); hindlimb 27.46–38.83% SVL (n = 28, mean = 33.73, SD = 0.02); digits moderate with no or only slight distal expansion; subdigital lamellae granular (not a clearly defined series except for small distal pair which tend to be long and narrow); 8–15 lamellae beneath fourth finger (n = 28, mean = 11.46, mode = 13, SD = 1.69);10–17 lamellae beneath fourth toe (n = 28, mean = 13.18, mode = 13, SD = 1.52). **Original tail:** short, wide 41.16–58.1% tail length (n = 22, mean = 46.66, SD = 0.04), with a short to moderate, acute attenuated extension at tip (Fig. 6E); scales arranged in clear transverse bands which incorporate rows of both large and small scales (often in a pattern of one large row followed by two small rows, of which the scales in the small rows are much less than ½ the size of the scales in the large rows (Fig. 6E); each large scale bears a short blunt to sharp medial tubercle); 31–49 (n = 23, mean = 40.09, mode = 41, SD = 4.73) medial scale rows on tail from fracture plane (1st autotomy septum) to tip; 10–15 (n = 24, mean = 13.21, mode = 14, SD = 1.25) rows of scales across original tail (large row at maximum width); ventral scales considerably smaller than dorsal scales. **Regrown tail:** with rounded distal end and more uniform scalation that is not arranged in clear transverse rows.

Measurements and scale counts of holotype. WAM R174500 (male, Fig. 13). SVL = 47.45mm, AG = 23.65mm, L1 = 13.45mm, L2 = 115.97mm, HL = 8.92mm, HD = 4.97mm, HW = 7.91mm, S = 4.27mm, EE = 2.45mm, TL = ~16.89mm (tail bent sideways during preservation), TW = 9.8mm, scales contacting posterior edge of rostral = 6, scales contacting posterior edge of mental = 10, lamellae beneath 4th finger = 13, lamellae beneath 4th toe = 14, medial scale rows on tail from fracture plane (1st autotomy septum) to tip = 37, rows of scales across original tail 14, supralabials = 14, infralabials = 20.

Pattern. Variable. Generally reddish- brown or grey. Most specimens with a series of irregular, dark wavy bands across back that usually extend across the vertebral zone (only one specimen, WAM R110027 has an unbroken, paler vertebral zone). There is usually some degree of fine spotting on back and flanks and in some specimens the spots extend across the dorsum in transverse rows. The delineation between the base colour and darker dorsal patterns ranges from moderate to sharply contrasting. Head generally with dark crown. A prominent, pale canthal stripe present, extending from anterior edge of orbit to tip of snout and producing a distinctive 'v' shaped marking which contrasts with the darker dorsal and lateral head markings. A broad dark zone on side of face extends posteriorly beyond eye to temporal region. A pale zone below eye extends to ear. Limbs mottled or spotted and inner digits of forelimb with reduced pigmentation. Ventral surfaces off-white, immaculate.

Comparison. *Diplodactylus bilybara* **sp. nov**. is readily distinguished from *D. platyurus* in possessing an enlarged first supralabial that contacts the ventral edge of the nasal scale (*vs* 1st supralabial small and not differentiated from the rest of the supralabial row). It is distinguished from *D. conspicillatus*, *D. hillii* and *D. barraganae* **sp. nov**. by the shape of its original tail (tail with short to moderate, acute attenuated extension at tip in *D. bilybara* **sp. nov**. *vs* tail blunt, spade-like without an attenuated tip). It is distinguished from *D. laevis* by the condition of the scales on the nape and top of head (scales granular and not appreciably larger than those on sides of neck in *D. bilybara* **sp. nov**. *vs* scales plate-like, appreciably larger than those on the sides of the neck). *D. bilybara* **sp. nov**. is most like *D. custos* **sp. nov**. but differs from this species in the following respects: distal half of original tail with alternating rows of large and small

scales (generally 1 large row followed by 2 small rows)—scales in the small rows ∼ ¼ the size of the scales in the adjacent large rows *vs* tail scalation generally more uniform; if smaller scale rows present, these rarely form a double row and the small scales are ∼ ½ the size of the scales in the adjacent large rows for *D. custos* **sp. nov.**; dark pigment on crown and snout contrast markedly with pale canthal stripe and lower jaw colour which extends posteriorly towards the ear as a pale bar *vs* dark pigment on crown and snout generally not contrasting sharply with pale canthal stripe and lower jaw colour for *D. custos* **sp. nov.**, trunk heavily pigmented and pattern usually incorporating numerous small pale spots *vs* body pattern often diffuse and generally without numerous pale spots, usually with wavy, dark transverse bands across back for *D. custos* **sp. nov.**.

Distribution and Ecology. Occurs in the Carnarvon, west Pilbara and west Gascoyne regions along the central west coast of Western Australia (Fig. 3). It is most abundant on less rocky habitats such as *Triodia* sandplains and Mulga woodlands on sandy loam substrates (B. Maryan pers. com.).

Diplodactylus custos **Couper, Oliver & Pepper sp. nov**.

urn:lsid:zoobank.org:act:303FDF6F-03BA-45A5-B2A9-10F86E98A799

'conspicillatus' E (Oliver et al. 2009)

Kimberley Fat-tailed gecko

Figs. 6F, 7H, 14

Holotype. WAM R164780, The Grotto (15° 43' 04" S, 128° 15' 35" E) WA.

Paratypes. WAM R77417, Port Warrender (14° 34' S, 125° 48' 15" E) WA; WAM R78243, Mitchell Plateau (14° 44' S, 125° 44' E) WA; WAM R172916, Doongan Stn (15° 13' 44.5" S, 125° 12' 30.4" E) WA; WAM R132713, 30km SSE Wyndham (15° 42' 43" S, 128° 15' 56" E) WA; SAMA R63942, The Grotto (15° 43' 04" S, 128° 15' 35" E) WA; WAM R162453, 20km W Kununurra (15° 45' 59" S, 128° 40' 18" S) WA;WAM R85120-21, Kununurra (15° 46' S, 128°

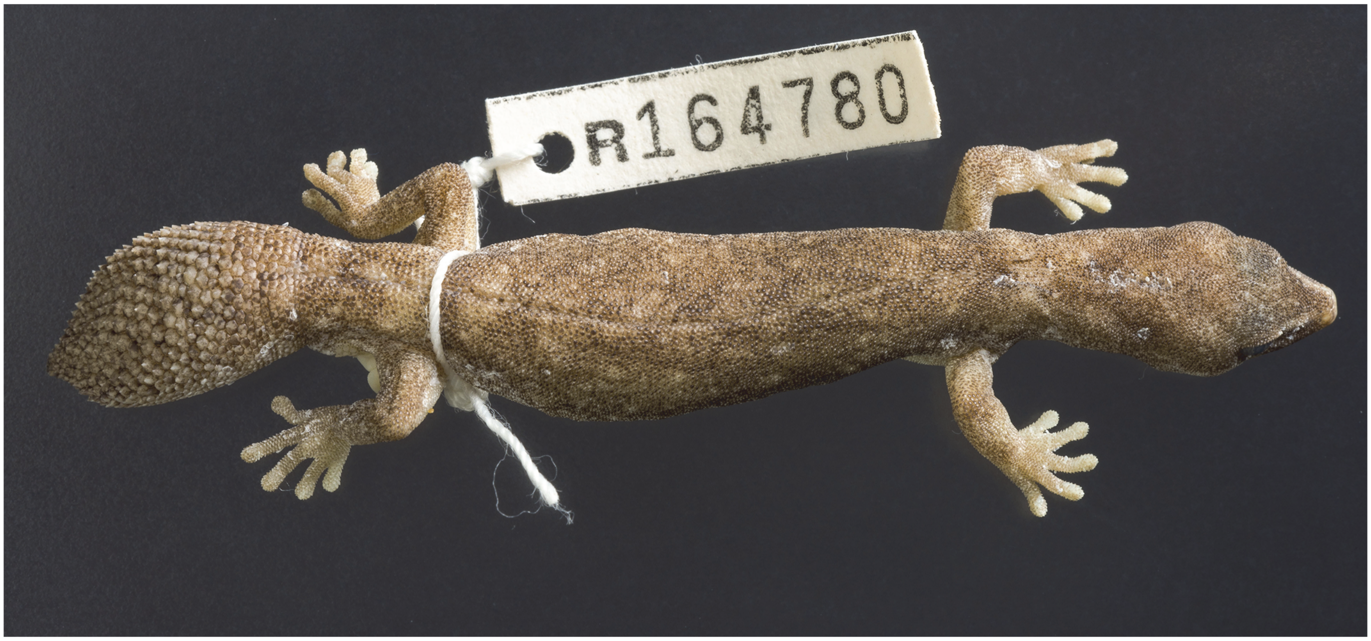

Fig 14. Holotype of *D. custos* sp. nov. (WAM R164780). The Grotto, Western Australia. (Image: Peter Waddington, QM).

doi:10.1371/journal.pone.0111895.g014

44' E) WA; WAM R119666, Cockburn Ra. (15° 50' S, 128° 02' E) WA; WAM R172853, Ellenbrae Stn (15° 59' 02" S, 127° 03' 14" E) WA; WAM R145042, Koolan Island (16° 07' 54" S, 123° 45' 29" E) WA; WAM R11255, Wotjulum (16° 11' S, 123° 37' E) WA; WAM R172675, Talbot Bay (16° 20' 07" S, 124° 03' 10" E) WA; WAM R70374, near Lissadell homestead (16° 40' S, 128° 23' 13" E) WA; WAM R103420, WAM R103448, Bungle Bungle NP (17° 24' S, 128° 45' E) WA.

Etymology. From the latin for guard, with reference to the Australian Wildlife Conservancy (AWC) and their ambitious and effective conservation and research programs in the Kimberley (where this species is endemic) and elsewhere in Australia. Used as a noun in apposition.

Diagnosis. A large member of the *D. conspicillatus* group (max SVL 61 mm) with a well-defined canthal stripe and a greatly enlarged first supralabial (first supralabial contacts ventral edge of nasal scale). Mid-dorsal scales on trunk plate-like and markedly larger than smaller dorsolaterals. Scales on nape granular and only slightly larger than granules on side of neck. Original tail with a short, acute attenuated extension at tip (Fig. 6F); scales on dorsal surface arranged in transverse rows generally of uniform size but if smaller scale rows are present, these rarely form a double row and the small scales are ~ ½ the size of the scales in the adjacent large rows (Fig. 6F). Dark pigment on crown and snout generally not contrasting sharply with pale canthal stripe and lower jaw colour. Body pattern often diffuse and generally without numerous pale spots; may incorporate wavy, dark transverse bands.

Description. SVL mm 42.17–58.23 (n = 13, mean = 50.58, SD = 5.75). Proportions as % SVL: AG 45.7–52.75 (n = 10, mean = 49.32, SD = 0.02); T 34.91–51.34 (n = 11, mean = 42.13, SD = 0.04); HL 16.34–21.15 (n = 13, mean = 18.85, SD = 0.02). **Head:** moderate and not strongly differentiated from neck; snout longer than diameter of eye. HW 73.73–89.14% HL (n = 12, mean = 81.50, SD = 0.05); HD 42.5–52.5% HL (n = 12, mean = 47.3, SD = 0.03); S 38.78–48.32% HL (n = 12, mean = 44.51, SD = 0.03); EE 24.26–30.40% HL (n = 12, mean = 27.62, SD = 0.02). Covered in small granular scales; rostral shield large and lacking a medial groove, hexagonal with 5–8 scales contacting posterior edge of rostral scale (n = 14, mean = 5.57, mode = 5, SD = 0.94); mental scale sometimes with a slight process on medial posterior margin; 10–15 scales contacting posterior edge (n = 16, mean = 12.25, mode = 12, SD = 1.50); supralabial scales 14–18 (n = 15, mean = 15.93, mode = 16, SD = 1.12) with the first enlarged and contacting ventral edge of nasal scale, the remaining series are small and not differentiated from the adjacent loreal scales; infralabial scales 13–21 (n = 14, mean = 16.86, mode = 16, SD = 2.71) all small and undifferentiated from adjacent chin scales; eye large, pupil vertical with crenulated margin; ear small, round to horizontally elliptic. **Neck:** broad with small granular scales on dorsal surface that are only slightly larger than the adjacent scales on the lateral surfaces. **Trunk:** moderate and somewhat stout; scales of mid-dorsum plate-like and markedly larger than smaller dorsolateral scales; granules small on ventral surface but increase in size on pectoral region; preanal pores absent; a small cluster of postanal tubercles present in both sexes but larger and more prominent in males. **Limbs:** moderate; forelimb 26.79–35.62% SVL (n = 13, mean = 31.06, SD = 0.03); hindlimb 28.13–39.82% SVL (n = 13, mean = 33.5, SD = 0.04); digits moderate with slight distal expansion; subdigital lamellae granular and not a clearly defined series (except for small distal pair which tend to be broadly oval and displaced laterally by claw); 9–16 lamellae beneath fourth finger (n = 15, mean = 11, mode = 11, SD = 1.60); 10–15 lamellae beneath fourth toe (n = 15, mean = 11.93, mode = 12, SD = 1.16). **Original tail:** short, wide 35–54.72% tail length (n = 10, mean = 46.0, SD = 0.05) with a short, acute attenuated extension on tip (Fig. 6F); Original tail with scales arranged in clear transverse rows which are largely of uniform size (where rows of smaller scales occur, they are ≥ ½ the size of the scales in the larger scale rows) each scale bearing a bluntly-tipped medial tubercle (Fig. 6F); 28–41 (n = 12, mean = 33.42, mode = 32, SD = 3.90) medial scale rows on tail from fracture plane (1st autotomy

septum) to tip; 12–14 (n = 12, mean = 12.92, mode = 12, SD = 0.90) rows of scales across original tail (large row at maximum width); ventral scales considerably smaller than dorsal scales. **Regrown tail:** with rounded distal end and more uniform scalation that is not arranged in clear transverse rows.

Measurements and scale counts of holotype. WAM R164780 (male, Fig. 14). SVL = 56.36mm, AG = 27.73mm, L1 = 15.41mm, L2 = 17.61mm, HL = 9.21mm, HD = 4.5mm, HW = 8.21mm, S = 4.45mm, EE = 2.8mm; TL = 22.74mm, TW = 10.45mm, scales contacting posterior edge of rostral = 5, scales contacting posterior edge of mental = 13, lamellae beneath 4th finger = 12, lamellae beneath 4th toe = 15, medial scale rows on tail from fracture plane (1st autotomy septum) to tip = 30, rows of scales across original tail 12, supralabials = 16, infralabials = 20.

Pattern. Variable. Tan to grey with darker overlay. Flanks and dorsum not strongly contrasting with ground colour and with or without pale spotting. Vertebral zone broken by dark, obscure to well-formed transverse bars. A pale canthal stripe present, extending from anterior edge of orbit to tip of snout and producing a distinctive 'v' shaped marking that does not contrast sharply with other facial markings. A dark zone on side of face extends posteriorly beyond eye to temporal region. Limbs mottled or spotted and inner digits of forelimb with reduced pigmentation. Ventral surfaces off-white, immaculate.

Comparison. *Diplodactylus custos* **sp. nov**. is readily distinguished from *D. platyurus* in possessing an enlarged first supralabial that contacts the ventral edge of the nasal scale (*vs* 1st supralabial small and not differentiated from the rest of the supralabial row). It is distinguished from *D. conspicillatus*, *D. hillii* and *D. barraganae* **sp. nov**. by the shape of its original tail (tail with short attenuated tip in *D. custos* **sp. nov**. *vs* tail blunt, spade-like without an attenuated tip). It is distinguished from *D. laevis* by the condition of the scales on the nape and top of head (scales granular and not appreciably larger than those on sides of neck in *D.custos* **sp. nov**. *vs* scales plate-like, appreciably larger than those on the sides of the neck). *D. custos* **sp. nov**. is most like *D. bilybara* **sp. nov**. but differs from this species in the following respects: scalation of original tail reasonably uniform; if smaller scale rows present, these rarely form a double row and the small scales are ∼ ½ the size of the scales in the adjacent large rows *vs* distal half of original tail with alternating rows of large and small scales (generally 1 large row followed by 2 small rows)—scales in the small rows ∼ ¼ the size of the scales in the adjacent large rows for *D. bilybara* **sp. nov**.; dark pigment on crown and snout generally not contrasting sharply with pale canthal stripe and lower jaw colour *vs* dark pigment on crown and snout contrast markedly with pale canthal stripe and lower jaw colour which extends posteriorly towards the ear as a pale bar in *D. bilybara* **sp. nov**.; body pattern often diffuse and generally without numerous pale spots, usually with wavy, dark transverse bands across back *vs* trunk heavily pigmented and pattern usually incorporating numerous small pale spots for *D. bilybara* **sp. nov**. Additionally, *D. custos* **sp. nov**. usually has a shorter and less pronounced acute attenuated extension on the original tail tip than *D. bilybara* **sp. nov**.

Distribution and Ecology. Known from widespread but scattered localities from across the Kimberley region of north-western Australia, ranging from Kununurra in the north-west, south to Purnululu National Park, to around Derby in the south-west, with additional records from the Yampi Peninsula and high rainfall zone of the north-west Kimberley. Has been recorded from Koolan Island off the west Kimberley, the only insular record of a member of the *D. conspicillatus* complex (Fig. 3).

A single specimen from Ellenbrae Station in the central Kimberley was collected from open *Eucalyptus* woodland on the top of a stony rise with heavy clay soils, while specimens from around Kununurra were collected from rocky hillsides vegetated with open grassy woodland (P. Oliver pers obs).

Key to the *Diplodactylus conspicillatus* species group

1) 1st supralabial enlarged, contacting ventral edge of nasal scale (Fig. 4C) and prominent, pale canthal stripe present (Fig. 4A)—**2**
   1st supralabial small, subequal to the rest of the supralabial row (Fig. 4D) and no prominent canthal stripe (Fig. 4B)—***platyurus* (eastern Australia, Qld & NSW)**

2) Mid-dorsal scales small and only a little larger than the dorsolateral scales (Fig. 5D)—**3**
   Mid-dorsal scales conspicuously larger than the dorsolateral scales (Fig. 5C)—**4**

3) Original tail bearing alternating transverse rows of different sized scales and rows of larger scales each with a bluntly spinose, central tubercle. More than 30 scales along dorsal midline of tail from fracture plane to tip.(Fig. 6D)—***barraganae* (NW Qld & NE NT)**
   Original tail without clearly defined transverse rows of different sized scales; scales large and slightly spinose (pine cone-like appearance). Fewer than 30 scales on dorsal midline of tail, from fracture plane to tip.(Fig. 6C)—***hillii* (N NT)**

4) Scales on nape granular, not appreciably larger than those on side of neck (Fig. 5A)—**5**
   Scales on nape and top of head plate-like and appreciably larger than those on side of neck (Fig. 5B). Original tail terminating with an acute attenuated extension at tip (Fig. 6B)—***laevis***

5) Original tail terminating with a short to moderate, attenuated extension at tip (Figs 6E & F)—**6**
   Original tail spade-like, lacking an acute attenuated extension at tip (Fig. 6A)—***conspicillatus***

6) Distal half of tail with alternating rows of large and small scales (generally 1 large row followed by 2 small rows); scales in the small rows ~ ¼ the size of the scales in the adjacent large rows (Fig. 6E). Dark pigment on crown and snout contrast markedly with pale canthal stripe and lower jaw colour which extends posteriorly towards the ear as a pale bar (Fig. 7G). Trunk heavily pigmented and pattern usually incorporating numerous small pale spots—***bilybara* (Pilbara, WA)**
   Tail scalation generally more uniform; if smaller scale rows present, these rarely form a double row and the small scales are ~ ½ the size of the scales in the adjacent large rows (Fig. 6F). Dark pigment on crown and snout generally not contrasting sharply with pale canthal stripe and lower jaw colour (Fig. 7H). Body pattern often diffuse and generally without numerous pale spots; may incorporate wavy, dark transverse bands—***custos* (Kimberley, WA)**

Supporting Information

S1 Fig. Maximum Likelihood Phylogeny for complete dampling of the *Diplodactylus conspicillatus* complex and outgroups. Estimated from mitochondrial ND2 data using RAxML with Maximum Likelihood support Boostrap supports shown for key nodes.
(PDF)

S1 Table. A. Measures of inter-specific genetic diversity and divergence. B. Intra-specific measures of genetic diversity and divergence. C. Diversity and demographic summary statistics.
(DOCX)

S1 Appendix. Additional material examined.
(DOCX)

Acknowledgments

We thank Andrew Amey, Gavin Dally, Paul Doughty, Paul Horner, Carolyn Kovach, Mark Hutchinson, Steve Richards, Ross Sadlier, Katie Smith, Claire Stevenson, Linette Umbrello and Dane Trembath and for their assistance accessing specimens and data. Mike Lee provided extensive assistance with running ancestral state analysis. Hal Cogger, Ryan Ellis, Paul Horner, Mark Hutchinson, Brad Maryan, Ross Sadlier, Steve Wilson and Eric Vanderduys generously provided in life or type photos.

Author Contributions

Conceived and designed the experiments: PO PC MP. Performed the experiments: PO PC MP. Analyzed the data: PO PC MP. Contributed reagents/materials/analysis tools: PO PC MP. Wrote the paper: PO PC MP.

References

1. Ortiz-Jaureguizar E, Cladera GA (2006) Paleoenvironmental evolution of southern South American during the Cenozoic. J. Arid Environ. 66: 498–532.
2. Richardson JE, Weitz FM, Fay MF, Cronk QCB, Linder HP, et al. (2001) Rapid and recent origin of species richness in the Cape flora of South Africa. Nature 412: 181–183. PMID: 11449273
3. Cowling RM, Proches S, Partridge TC (2009) Explaining the uniqueness of the Cape Flora: incorporating geomorphic evolution as a factor for explaining its diversification. Mol. Phylogenet. Evol. 51: 64–74. doi: 10.1016/j.ympev.2008.05.034 PMID: 18691908
4. Martin HA (2006) Cenozoic climatic change and the development of the arid vegetation in Australia. J. Arid Environ. 66: 533–563.
5. Archer M, Hand SJ, Godthelp H (1991) 'Australia's Lost World: Prehistoric Animals of Riversleigh.' (Indiana University Press: Bloomington)
6. Pepper M, Fujita MK, Moritz C, Keogh JS (2011) Paleoclimate change drove diversification among isolated mountain refugia in the Australian arid zone. Mol. Ecol. 20: 1529–1545. doi: 10.1111/j.1365-294X.2011.05036.x PMID: 21371147
7. Pepper M, Doughty P, Hutchinson MN, Keogh JS (2011) Ancient drainages divide cryptic species in Australia's arid zone: Morphological and multi-gene evidence for four new species of Beaked geckos (*Rhynchoedura*). Mol. Phylogenet. Evol. 61: 810–822. doi: 10.1016/j.ympev.2011.08.012 PMID: 21884806
8. Oliver PM, Adams M, Doughty P (2010) Molecular evidence for ten species and Oligo-Miocene vicariance within a nominal Australian gecko species (*Crenadactylus ocellatus*, Diplodactylidae). BMC Evol. Biol. 10: 386. doi: 10.1186/1471-2148-10-386 PMID: 21156080
9. Byrne M, Yeates DK, Joseph L, Kearney M, Bowler J, et al. (2008) Birth of a biome: insights into the assembly and maintenance of the Australia arid zone biota. Mol. Ecol. 17: 4398–4417. doi: 10.1111/j.1365-294X.2008.03899.x PMID: 18761619
10. Byrne M, Steane D, Loseph L, Yeates D, Jordan GJ, et al. (2011) Decline of a biome: Evolution, contraction, fragmentation, extinction and invasion of the Australian mesic zone biota. J. Biogeog. 38: 1635–1656.
11. Chapple DG, Keogh JS (2004) Parallel adaptive radiations in arid and temperate Australia: molecular phylogeography and systematics of the *Egernia whitii* (Lacertilia: Scincidae) species group. Biol. J. Linn. Soc. 83: 157–173.
12. Oliver PM, Bauer AM (2011) Molecular systematics of the Australian gecko genus *Nephrurus*: plesiomorphic grades and progressive biome shifts through the Miocene. Mol. Phylogenet. Evol. 59: 664–674. doi: 10.1016/j.ympev.2011.03.018 PMID: 21421065
13. Toon A, Mather PB, Baker AM, Durrant KL, Hughes JM (2007) Pleistocene refugia in an arid landscape: analysis of a widely distributed Australian passerine. Mol. Ecol. 16: 2525–41. PMID: 17561911
14. Fujita MK, McGuire JA, Donnellan SC, Moritz CM (2010) Diversification at the arid-monsoonal interface: Australia-wide biogeography of the Bynoe's gecko (*Heteronotia binoei*; Gekkonidae). Evolution 64: 2293–2314. doi: 10.1111/j.1558-5646.2010.00993.x PMID: 20298463
15. Marin J, Donnellan SC, Blair Hedges SB, Doughty P, Hutchinson MN, et al. (2013) Tracing the history and biogeography of the Australian blindsnake radiation. J. Biogeogr. 40: 928–937.

16. Oliver PM, Smith KL, Laver RL, Doughty P, Adams M (2014) Contrasting patterns of persistence and diversification in vicars of a widespread Australian lizard lineage (the *Oedura marmorata* complex). J. Biogeogr. *In press*.

17. Bowman DMJS, Brown GK, Braby MF, Brown JR, Cook LG, et al. (2010) Biogeography of the Australian monsoon tropics. J. Biogeogr. 37: 201–216.

18. Kuch U, Keogh JS, Weigel J, Smith LA, Mebs D (2005) Phylogeography of Australia's king brown snake (Pseudechis australis) reveals Pliocene divergence and Pleistocene dispersal of a top predator. Naturwissenschaften 92: 121–127. PMID: 15688185

19. Pianka ER (1986) Ecology and Natural History of Desert Lizards. Princeton University Press.

20. Powney GD, Grenyer R, Orme CDL, Owens PF, Meiri S (2010) Hot, dry and different: Australian lizard richness is unlike that of mammals, amphibians and birds. Global Ecol.Biogeogr. 19: 386–396.

21. Rabosky DL, Donnellan SC, Talaba AL, Lovette IJ (2007) Exceptional among-lineage variation in diversification rates during the radiation of Australia's most diverse vertebrate clade. Proc. Roy. Soc. B. 274: 2915–23. PMID: 17878143

22. Donnellan S, Adams M, Hutchinson M, Baverstock PR (1993) The identification of cryptic species in the Australian herpetofauna: a high research priority. In Herpetology in Australia: a diverse discipline (eds Lunney D. & Ayres D.), pp. 121–126. Sydney, Australia: Surrey Beatty & Sons.

23. Oliver PM, Doughty P, Palmer R (2012) Hidden biodiversity in rare northern Australian vertebrates: the case of the clawless geckos (*Crenadactylus*, Diplodactylidae) of the Kimberley. Wildlife Research 39: 429–435.

24. Pepper M, Doughty P, Keogh JS (2013) Geodiversity & endemism in the iconic Australian Pilbara region: A review of landscape evolution and biotic response in an ancient refugium. J. Biogeogr. 40: 1225–1239.

25. Oliver PM, Hutchinson MN, Cooper SJB (2007) Phylogenetic relationships in the lizard genus *Diplodactylus* Gray and resurrection of *Lucasium* Wermuth (Gekkota, Diplodactylidae). Aust. J. Zool. 55: 197–210.

26. Melville J, Ritchie E, Chapple SNJ, Glor RE, Schulte JA II (2011) Evolutionary origins and diversification of dragon lizards in Australia's tropical savannah woodlands. Mol. Phylogenet. Evol. 58: 257–270. doi: 10.1016/j.ympev.2010.11.025 PMID: 21145401

27. Smith K, Harmon LJ, Shoo LP, Melville J (2011) Evidence of constrained phenotypic evolution in a cryptic species complex of agamid lizards. Evolution, 65:976–992. doi: 10.1111/j.1558-5646.2010.01211.x PMID: 21166790

28. Lucas AHS, Frost C (1897) Descriptions of two new species of lizards from central Australia. Proceedings of the Royal Society of Victoria (ns): 54–56.

29. Cogger HG, Cameron EE, Cogger HM (1983) Zoological Catalogue of Australia 1 Amphibia and Reptilia. Australian Government Publishing Service, Canberra.

30. Oliver P, Doughty P, Hutchinson M Lee MSY, Adams A (2009) The taxonomic impediment in vertebrates: DNA sequence, allozyme and chromosomal data double estimates of species diversity in a lineage of Australian lizards (*Diplodactylus*, Gekkota). Proc. Roy. Soc. B: 276,2001–2007.

31. Pepper M, Doughty P, Keogh JS (2006) Molecular phylogeny and phylogeography of the Australian *Diplodactylus stenodactylus* (Gekkota; Reptilia) species-group based on mitochondrial and nuclear genes reveals an ancient split between Pilbara and non-Pilbara *D*. *stenodactylus*. Mol. Phylogenet. Evol. 41: 539–555. PMID: 16843684

32. Macey JR, Larson A, Ananjeva NB, Fang Z, Papenfuss TJ (1997) Two novel gene orders and the role of light- strand replication in rearrangement of the vertebrate mitochondrial genome. Mol. Biol. Evol. 14: 91–104. PMID: 9000757

33. Read K, Keogh JS, Scott IAW, Roberts JD, Doughty P (2001) Molecular phylogeny of the Australian frog genera *Crinia*, *Geocrinia* and allied taxa (Anura: Myobatrachidae). Mol. Phylogenet. Evol. 21: 294–308. PMID: 11697923

34. Edgar RC (2004) MUSCLE: multiple sequence alignment with high accuracy and high throughput. Nuc. Acids. Res. 32: 1792–1797. PMID: 15034147

35. Rambaut A (1996) Se–Al. Sequence alignment editor, Version 1.0 alpha 1 Department of Zoology. University of Oxford, Oxford.

36. Lanfear R, Calcott B, Ho SYW, Guindon S (2012) PartitionFinder: combined selection of partitioning schemes and substitution models for phylogenetic analyses. Mol. Biol. Evol. 29: 1695–1701. doi: 10.1093/molbev/mss020 PMID: 22319168

37. Stamatakis A (2006) RaxML-VI-HPC: Maximum Likelihood-based Phylogenetic Analyses with thousands of taxa and mixed models. Bioinformatics 22: 2688–2690. PMID: 16928733

38. Drummond AJ, Rambaut A (2007) BEAST: Bayesian evolutionary analysis by sampling trees. BMC Evol. Biol. 7: 214. PMID: 17996036
39. Rambaut A, Drummond AJ (2007) Tracer v1.4, Available: http://beast.bio. edu.ac.uk/Tracer.
40. Lewis PO (2001) A likelihood approach to estimating phylogeny from discrete morphological character data. Syst. Biol. 50: 913–925. PMID: 12116640
41. Tajima F (1989) Statistical method for testing the neutral mutation hypothesis by DNA polymorphism. Genetics 123: 585–595. PMID: 2513255
42. Fu YX (1997) Statistical tests of neutrality of mutations against population growth, hitchhiking and background selection. Genetics 147: 915–925. PMID: 9335623
43. Ramos-Onsins SE, Rozas J (2002) Statistical properties of new neutrality tests against population growth. Mol. Biol. Evol. 19: 2092–2100. PMID: 12446801
44. Librado P, Rozas J (2009) DnaSP v5: a software for comprehensive analysis of DNA polymorphism data. Bioinformatics 25:1451–1452. doi: 10.1093/bioinformatics/btp187 PMID: 19346325
45. Excoffier L, Laval G, Schneider S (2005) Arlequin (version 3.0): an integrated softwarepackage for population genetics data analysis. Evol. Bioinformatics Online 1: 47–50. PMID: 19325852
46. De Queiroz K (2007) Species concepts and species delimitation. Syst. Biol. 56: 879–886. PMID: 18027281
47. Padial JM, Miralles A, De la Riva I, Vences M (2010) The integrative future of taxonomy. Frontiers in Zoology 7:16. doi: 10.1186/1742-9994-7-16 PMID: 20500846
48. Ballard JWO, Whitlock MC (2004) The incomplete natural history of mitochondria. Molecular Ecology 13:729–744. PMID: 15012752
49. Catullo R, Lanfear R, Doughty P, Keogh JS (2014) The biogeographical boundaries of northern Australia: evidence from ecological niche models and a multi-locus phylogeny of Toadlets (*Uperoleia*: Myobatrachidae). J. Biogeogr. 41: 659–672. doi: 10.1016/j.jgg.2014.08.008 PMID: 25527107
50. Eldridge MDB, Potter S, Cooper SJB (2012) Biogeographic barriers in north-western Australia: an overview and standardization of nomenclature. Aust. J. Zool. 59: 270–272. doi:10.1071/ZO12012
51. Rollins LA, Svedin N, Pryke SR, Griffith SC (2012) The role of the Ord Arid Inrusion in the historical and contempory division of long-tailed finch subspecies in northern Australia. Ecol. Evol. 2: 1208–1219. doi: 10.1002/ece3.259 PMID: 22833795
52. MacDonald JD (1969) Notes on the taxonomy of *Neositta*. Emu 69:169–174.
53. Allen GR, Feinberg MN (1998) Descriptions of a new genus and four new species of freshwater catfishes (Plotosidae) from Australia. Aqua—Journal of Ichthyol. Aqua. Biol. 3: 9–18.
54. Jaensch R (2009) Floodplain wetlands and waterbirds of the Channel Country. South Australian Arid Lands Natural Resources Management Board, Adelaide.
55. Alley NF (1998) Cainozoic stratigraphy, palaeoenvironments and geological evolution of the Lake Eyre Basin. Palaeogeogr, Palaeoclimat. Palaeoecol. 144: 239–26.
56. Fujioka T, Chappell J, Fifield LK, Rhodes EJ (2009) Australian desert dune fields initiated with Pliocene-Pleistocene global climatic shift. Geology 37: 51–54.
57. Williams KJ, Ferrier S, Rosauer D, Yeates D, Manion G, et al. (2010) Harnessing Continent-Wide Biodiversity Datasets for Prioritising National Conservation Investment. A report prepared for the Department of Sustainability, Environment, Water, Population and Communities. CSIRO Ecosystem Sciences, Canberra. ISBN 978 0 643 10342 9.
58. Atlas of living Australia. Available: http://www.ala.org.au/. Accessed 2014 May 5.
59. Adams M, Foster R, Hutchinson MN, Hutchinson RG, Donnellan SC (2003) The Australian scincid lizard *Menetia greyii*: a new instance of widespread vertebrate parthenogenesis. Evolution 57: 2619–2627. PMID: 14686536
60. Crisp MD, Arroyo MTK, Cook LG, Gandolfo MA, Jordan GJ, et al. (2009) Phylogenetic biome conservation on a global scale. Nature 458: 754–758. doi: 10.1038/nature07764 PMID: 19219025
61. Pepper M, Doughty P, Fujita MK, Moritz C, Keogh S (2013) Speciation on the Rocks: Integrated Systematics of the *Heteronotia spelea* Species Complex (Gekkota; Reptilia) from Western and Central Australia. PLOS One. DOI: 10.1371/journal.pone.0078110
62. Crisp MD, Cook LG, Steane D (2004) Radiation of the Australian flora: what can comparisons of molecular phylogenies across multiple taxa tell us about the evolution of diversity in present-day communities? Phil. Trans. Roy. Soc. B 359: 1551–1571. PMID: 15519972
63. Jennings WB, Pianka ER, Donnellan S (2003) Systematics of the lizard family Pygopodidae with implications for the diversification of Australia temperate biotas. Syst. Biol. 52: 757–780. PMID: 14668116

64. Uetz P, Hosek J (2013) The Reptile Database. Available: http://www.reptile-database.org. Accessed 2013 Nov 10.

65. Shea G, Couper P, Worthington Wilmer J, Amey A (2011) Revision of the genus *Cyrtodactylus* Gray, 1827 (Squamata: Gekkonidae) in Australia. Zootaxa 3146: 1–63. doi: 10.1109/IEMBS.2011.6090858 PMID: 22255007

66. Sistrom M, Donnellan S, Hutchinson M (2013) Delimiting species in recent radiations with low levels of morphological divergence: a case study in Australian *Gehyra* geckos. Mol. Phylogenet. Evol. 68: 135–143. doi: 10.1016/j.ympev.2013.03.007 PMID: 23507430

67. Marin J, Donnellan SC, Hedges SB, Puillandre N, Aplin KP, et al. (2013b) Hidden species diversity Of Australian burrowing snakes (*Ramphotyphlops*). Biol. J. Linn. Soc. 110: 427–441.

68. Horner P, Adams M (2007) A molecular systematic assessment of species boundaries in Australian *Cryptoblepharus* (Reptilia: Squamata: Scincidae)—a case study for the combined use of allozymes and morphology to explore cryptic biodiversity. The Beagle, Rec. Mus. Art. Gall. North. Terr., Supp. 3: 1–19.

69. Smith LA, Adams M (2007) Revision of the *Lerista muelleri* species-group (Lacertilia: Scincidae) in Western Australia, with a redescription of *L. muelleri* (Fischer, 1881) and the description of nine new species. Rec. West. Austr. Mus. 23: 309–358.

70. Dolman G, Hugall AF (2008) Combined mitochondrial and nuclear data enhance resolution of a rapid radiation of Australian rainbow skinks (Scincidae: *Carlia*). Mol. Phylogenet. Evol. 49: 782–794. doi: 10.1016/j.ympev.2008.09.021 PMID: 18930831

71. Fujita MK, Leaché AD, Burbrink FT, McGuire JA, Moritz C (2012) Coalescent-based species delimitation in an integrative taxonomy. Trends Ecol. Evol. 27: 480–488. doi: 10.1016/j.tree.2012.04.012 PMID: 22633974

72. Bickford D, Lohman DJ, Sodhi NS, Ng PK, Meier R, et al. (2007) Cryptic species as a window on diversity and conservation. Trends Ecol. Evol. 22: 148–155. PMID: 17129636

73. Longman HA (1915) Reptiles from Queensland and the Northern Territory. Mem. Queens. Mus. 3: 30–34.

74. Sternfield R (1924) Beiträge zur Herpetologie Inner-Australiens. Abh. Senckenb. Naturf. Ges. 38: 221–251.

75. Parker HW (1926) New reptiles and a new frog from Queensland. Ann. Mag. Nat Hist. 17: 665–670.

76. Kluge AG (1963) The systematic status of certain Australian and New Guinean gekkonid lizards. Mem. Queens. Mus. 14: 77–86.

77. Kluge AG (1967) Systematics, phylogeny, and zoogeography of the lizard genus *Diplodactylus* Gray (Gekkonidae). Aust. J. Zool. 15: 1007–1108.

78. Wells RW, Wellington RC (1984) A Synopsis of the Class Reptilia in Australia. Aust. J. Herp. 73–129.

79. Storr GM, Smith LA, Johnstone RE (1990) *Lizards of Western Australia III*: *geckos and pygopods*. Western Australian Museum Press: Perth.

80. Mertens R (1967) Die herpetologische Sektion des Natur-Museums und Forschungs-Institutes Senckenberg in Frankfurt am Main nebst einem Verzeichnis ihrer Typen. Senckenbergiana Biologica 48: 1–106. PMID: 11633150
